# Supplementary material for: Deciphering the mechanism of the Ni-photocatalyzed C‒O cross-coupling reaction using a tridentate pyridinophane ligand
Source: Nat Commun. 2022 Mar 14;13:1313. doi: 10.1038/s41467-022-28948-8 (PMC8921334; doi:10.1038/s41467-022-28948-8)
Supplement: Supplementary file 1 — Supplementary Information [file 41467_2022_28948_MOESM1_ESM.pdf]

Supplementary Information for

Deciphering the mechanism of the Ni-photocatalyzed C–O cross-coupling reaction using a tridentate pyridinophane ligand

Hanah Na and Liviu M. Mirica*

Department of Chemistry, University of Illinois at Urbana-Champaign, Urbana, Illinois, 61801

**E-mail: mirica@illinois.edu*

Table of Contents

1. Supplementary Methods S2
   1.1 General specifications S2
   1.2. Synthesis S3
   1.3 NMR spectra S10
   1.4 Cyclic voltammograms of Ni complexes S20
   1.5 UV-vis absorption spectra S23
   1.6 Nickel catalyzed C–O coupling experiments S26
   1.7 Light on/off experiments S29
   1.8 Quantum yield measurements S31
   1.9 ReactIR experiments S34
   1.10 Comproportionation studies S37
   1.11 Electron paramagnetic resonance (EPR) experiments S42
   1.12 Reactivity study of $^{iPr}N3Ni^{III}$ species S57
   1.13 X-ray structure Characterization S60
   1.14 Space filling model of 1a and 1b S68
   1.15 X-ray photoelectron spectroscopy (XPS) experiments S69
   1.16 DFT calculations S70
2. Supplementary Discussion S98
   2.1 Thermodynamic feasibility examination of the photocatalytic process S98
3. Supplementary References S100

1. Supplementary Methods

1.1 General specifications

Materials. Reactions were performed in ambient conditions unless otherwise stated. Solvents were purified prior to use by passing through a column of activated alumina using an M Braun solvent purification system. All starting materials and reagents, unless otherwise specified, were obtained from commercial sources and used without further purification.

Physical Methods. ^{1}H and ^{13}C NMR spectra were recorded using a Bruker 400 MHz or 500 MHz spectrometer. UV-vis absorption spectra were recorded in CH_2Cl_2 in 1 cm quartz cuvettes using a Varian Cary 60 spectrophotometer. EPR spectra were recorded on a Bruker 10" EMXPlus X-band Continuous Wave EPR spectrometer at 77 K. EPR spectra simulation and analysis were performed using Bruker WINEPR SimFonia program, version 1.25. The spin state of Ni^{III} complex was determined using ^{1}H NMR method of Evans at –40 °C.[1,2] Elemental analysis was carried out by the Microanalysis Laboratory at University of Illinois at Urbana-Champaign (UIUC) using an Exeter Analytical -Model CE440 CHN Analyzer. Cyclic voltammetry (CV) experiments were performed with a CHI 660D Electrochemical Analyzer using a three-electrode system in a nitrogen-filled glove box. A 3mm diameter glassy-carbon electrode, Pt wire, and silver wire were used as working electrode, counter electrode, and pseudoreference electrode, respectively. Measurements were carried out in acetonitrile or dichloromethane solution with 0.1 M $TBAPF_6$ as a supporting electrolyte at a scan rate of 0.1 V/s. Ferrocene was used as an internal standard, and potentials were referenced to the ferrocene/ferrocenium couple. The X-ray Photoelectron Spectroscopy (XPS) experiments were performed at the Materials Research Laboratory at UIUC using Kratos Axis ULTRA X-ray Photoelectron Spectrometer. Photocatalysis experiments were carried out using one Kessil lamp model PR160 blue LED (λ_{max} = 456 nm, max 50W) or purple LED (λ_{max} = 390 nm, max 52W). *In-situ* IR spectroscopy was performed on a Mettler Toledo ReactIR 15 instrument with a MCT detector and a 6 mm × 1.5 mm AgX DiComp (diamond) fiber conduit probe.

1.2. Synthesis

Reactions were performed in ambient conditions unless otherwise stated. Solvents were purified prior to use by passing through a column of activated alumina using an MBraun solvent purification system. All starting materials and reagents, unless otherwise specified, were obtained from commercial sources and used without further purification.

Synthesis of ligands

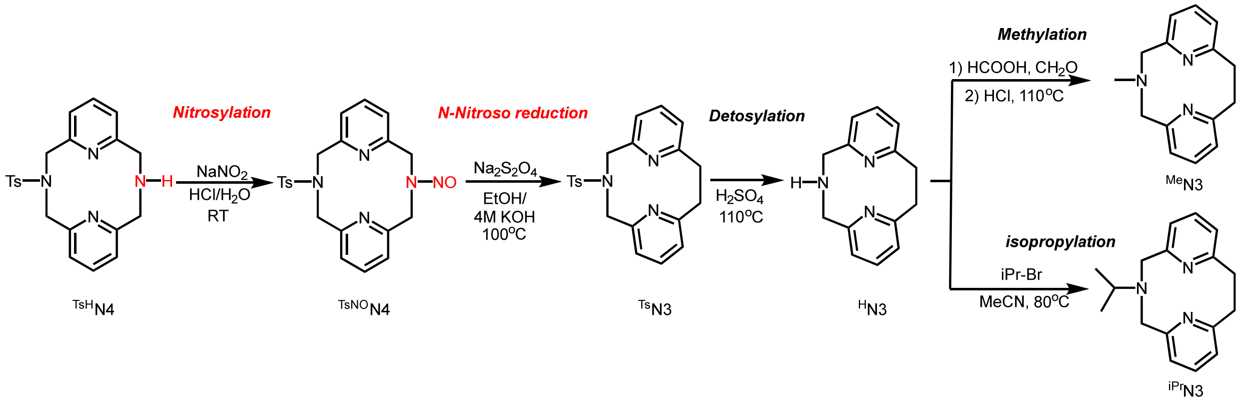

Supplementary Figure 1. Synthesis of ^{R}N3 ligands

<u>Synthesis of ^{TsH}N4 compound</u>

The starting material *N*-(tosyl)-2,11-diaza[3,3](2,6)pyridinophane (^{TsH}N4) was synthesized following our published procedure.[3] 2,11-diaza[3,3](2,6)pyridinophane (1.25 g, 5.20 mmol) and triethylamine (726 µL, 5.21 mmol) were dissolved in CH_2Cl_2 (150 mL) at 0 °C. To this solution, TsCl (0.9917 g, 5.20 mmol) in CH_2Cl_2 (450 mL) was added dropwise. After the addition was complete, the reaction was stirred at 0 °C for an additional three hours. The reaction mixture was washed with a solution of saturated sodium bicarbonate (100 mL). The organic layer was dried with potassium carbonate, filtered, and concentrated to dryness to give a white crude product mixture (1.74 g). The mixture was suspended in isopropanol (500 mL), stirred for 24 hours, and then filtered. The clear filtrate solution was dried to give a white solid product. Yield: ^{TsH}N4 (0.90 g, 44%). ^{1}H NMR (300 MHz, $CDCl_3$): 7.81 (d, J = 7.8 Hz, 2H), 7.39 (d, *J* = 7.8 Hz, 2H), 7.19 (t, *J* = 7.5 Hz, 2H), 7.06 (d, *J* = 7.5 Hz, 2H), 6.60 (d, *J* = 7.5 Hz, 2H), 4.52 (s, 4H), 3.91 (s, 4H), 2.48 (s, 3H).

Synthesis of ^{TsNO}N4 compound

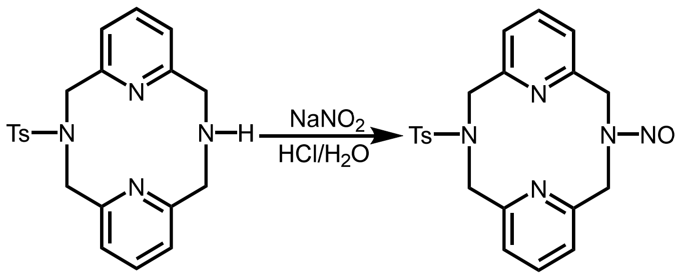

A cold solution of $NaNO_2$ 540 mg in 5mL of water was added to a solution of ^{TsH}N4 200 mg in 4 mL of conc. HCl. The mixture was left overnight at room temperature. Next day, yellowish-white solids were filtered and washed with Et_2O. Pale-yellow or white solid product were collected and dried. Yield: ^{TsNO}N4 (182 mg, 85%) ^{1}H NMR (500 MHz, DMSO-d_6) δ 8.40 (t, J = 7.8 Hz, 1H), 8.27 (t, J = 7.8 Hz, 1H), 8.00 (d, J = 7.8 Hz, 1H), 7.85 (d, J = 7.8 Hz, 1H), 7.81 – 7.73 (m, 4H), 7.45 (d, J = 8.0 Hz, 2H), 6.14 (s, 2H), 5.22 (s, 2H), 4.93 (s, 2H), 4.84 (s, 2H), 2.43 (s, 3H).

N-nitroso reduction reaction

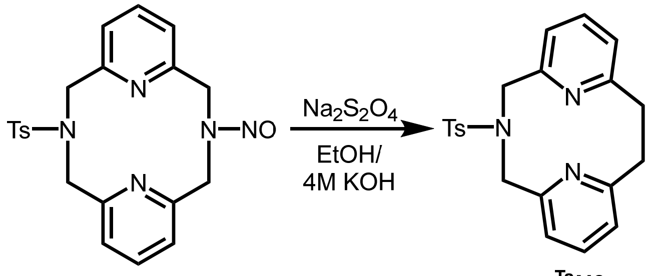

^{Ts}N3

Under N_2, the ^{TsNO}N4 compound (120 mg) was dissolved into a mixture of 4M KOH 10 mL and ethanol 10 mL and heated under reflux (100–110 °C). After stirring 1h, the reaction mixture was cooled down to room temperature. Then solid sodium hydrosulfite (176 mg) was quickly added to the reaction mixture. The reaction mixture refluxed at 110 °C for 3hr. Then the mixture was cooled down and poured into 40 mL of DI water. The resulting solution was extracted with CH_2Cl_2 (3×30 mL). Combined organic layers were dried over $MgSO_4$ and filtered. The clear solution was concentrated to dryness to give a white solid or yellowish-white solid. Yield: ^{TS}N3 (81 mg, 75%) ^{1}H NMR (400 MHz, Chloroform-d) δ 7.86 – 7.76 (m, 2H), 7.51 (t, J = 7.5 Hz, 2H), 7.35 (d, J = 8.0 Hz, 2H), 7.10 (d, J = 7.6 Hz, 2H), 6.95 (d, J = 7.6 Hz, 2H), 4.36 (s, 4H), 2.98 (s, 4H), 2.47 (s, 3H). ^{13}C NMR (126 MHz, $CDCl_3$) δ 160.45, 153.37, 143.62, 137.42, 130.24, 130.03, 127.51,

127.24, 122.50, 121.77, 54.24, 39.35, 21.84. HRMS-ESI (*m*/*z*): $[M + H]^+$ calcd for $C_{21}H_{21}N_3O_2S$, 380.1433; found 380.1440.

Synthesis of $^{Me}N3$ ligand

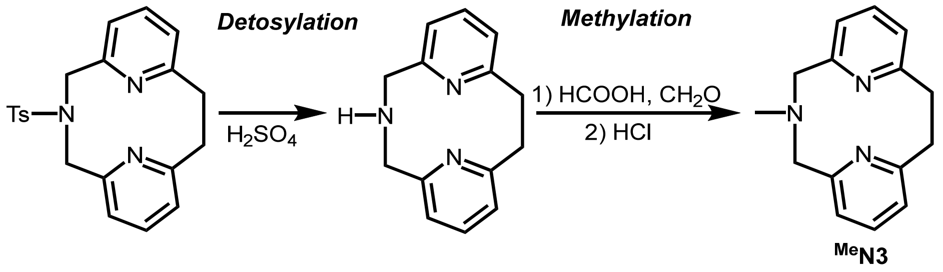

Detosylation

Under N_2, $^{Ts}N3$ 300 mg was dissolved in 15 mL of 90% sulfuric acid. This mixture was stirred and refluxed at 110°C for 2.5 hr. After cooling, the solution was treated with saturated KOH solution to make the pH 13. The resulting solution was extracted with CH_2Cl_2. The organic layer was dried over anhydrous K_2CO_3 and $MgSO_4$. The filtrate was dried to give an off-white solid. Yield: $^{H}N3$ (130 mg, 73 %) ^1H NMR (400 MHz, Chloroform-*d*) δ 7.54 (t, *J* = 7.5 Hz, 2H), 7.02 (dd, *J* = 17.6, 7.6 Hz, 4H), 3.87 (s, 4H), 3.01 (s, 4H).

Methylation

Under N_2, $^{H}N3$ 17 mg was dissolved in formic acid 2.5 mL and 40% formaldehyde solution 0.25 mL. The mixture was stirred and refluxed at 110°C for overnight. After cooling, the solution was treated with concentrated HCl 0.25 mL. After several minutes the solution was dried, and the residue was basified with NaOH solution and extracted with CH_2Cl_2. The organic layer was dried over anhydrous K_2CO_3 and $MgSO_4$. The filtrate was dried to give a slightly yellowish-white solid. Yield: $^{Me}N3$ (17 mg, 94%) ^1H NMR (400 MHz, Chloroform-*d*) δ 7.59 (t, *J* = 7.6 Hz, 2H), 7.17 – 7.08 (m, 4H), 3.69 (s, 4H), 3.00 (s, 4H), 2.76 (s, 3H). ^{13}C NMR (126 MHz, Chloroform-*d*) δ 160.26, 156.20, 136.95, 121.76, 120.95, 62.70, 62.67, 62.64, 47.23, 47.20, 47.18, 39.49. HRMS-ESI (*m*/*z*): $[M + H]^+$ calcd for $C_{15}H_{17}N_3$, 240.1501; found, 240.1492.

Synthesis of ^{iPr}N3 ligand

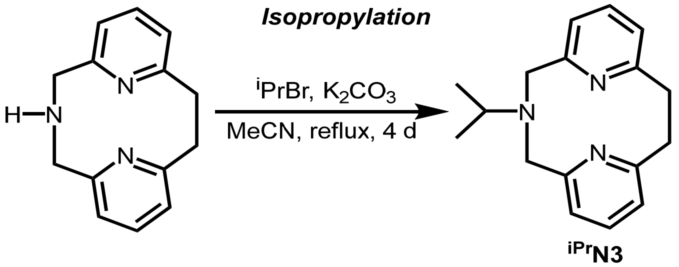

Isopropylation

^{H}N3 (390 mg, 1.73 mmol), isopropylbromide (8.1 mL, 86.5 mmol, 50 equiv), anhydrous K_2CO_3 (2.87 g, 20.7 mmol, 12 equiv), and dry MeCN (60 mL) were charged into a 200 mL Schlenk flask with a magnetic stir bar. The reaction mixture was refluxed under N_2 for 4 days. The solution was then cooled to room temperature, and the solvent was removed under reduced pressure. The residue was suspended in 100 mL of CH_2Cl_2 and then washed with 1M NaOH. The CH_2Cl_2 layer was isolated, and dried over $MgSO_4$, evaporated and further dried under vacuum to give a pale yellow solid. Yield: ^{iPr}N3 (416 mg, 90%) ^{1}H NMR (500 MHz, Chloroform-*d*) δ 7.51 (t, J = 7.6 Hz, 2H), 7.03 (ddd, J = 24.0, 7.6, 1.0 Hz, 4H), 3.74 (s, 4H), 3.23 – 3.18 (m, 1H), 3.00 (s, 4H), 1.26 (s, 6H). ^{13}C NMR (126 MHz, Chloroform-*d*) δ 159.98, 157.96, 136.87, 121.53, 121.29, 57.32, 56.27, 39.09, 19.96. HRMS-ESI (*m/z*): $[M + H]^+$ calcd for $C_{17}H_{21}N_3$, 268.1814; found 268.1812.

Synthesis of Ni complexes

Synthesis of (^{Me}N3)$NiCl_2$ (**1a**)

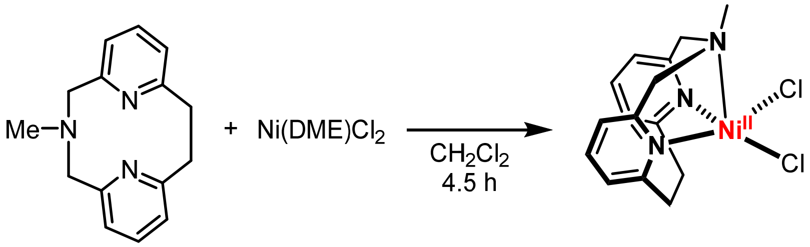

Inside of glovebox, Ni(DME)Cl_2 (29 mg) and ^{Me}N3 (32 mg) were dissolved in 10 ml of CH_2Cl_2 at room temperature. The yellow mixture was stirred for 4.5 h, slowly generating green precipitate. After 4.5 h, the precipitate was collected and dried under vacuum to afford highly insoluble green solids. Yield: 44 mg, 91%. The insolubility of this product prevents spectroscopic characterization. X-ray quality crystals were obtained by layering pentane and MeOH solution of the compound at

–35°C outside of the glovebox. Elemental analysis: found C 45.65, H 4.52, N 10.56%; calculated $C_{15}H_{17}Cl_2N_3Ni{\bullet}H_2O$ C 46.56, H 4.95, N 10.86%

Synthesis of (^{iPr}N3)$NiCl_2$ (**1b**)

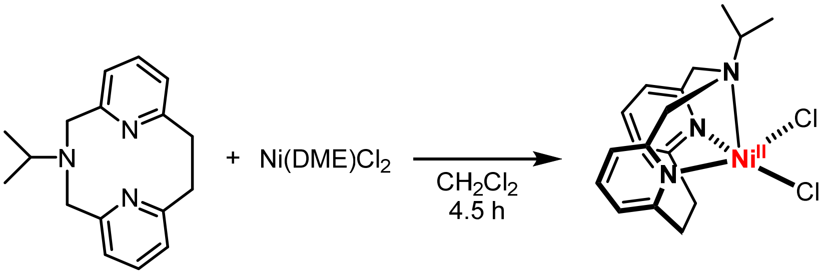

Inside of glovebox, $Ni(DME)Cl_2$ (27 mg, 0.12288 mmol) and ^{iPr}N3 (34 mg, 0.127 mmol) were dissolved in 10 mL of CH_2Cl_2 at room temperature. The yellow mixture was stirred for 4.5 h, and yellow color was slowly turned to green color. After 4.5h, the green precipitate was collected and dried under vacuum to afford bright green solids. Yield: 47 mg, 97 %. X-ray quality crystals were obtained by layering MeOH and CH_2Cl_2 solution of the compound at room temperature. ^{1}H NMR (500 MHz, CD_3OD) δ 68.82, 54.57, 42.18, 13.45. Evans method (CD_3OD): μ_{eff} = 3.03 μ_b. Elemental analysis: Found: C 43.76, H 4.77, N 8.61%; calculated $C_{17}H_{21}Cl_2N_3Ni{\bullet}1.2CH_2Cl_2$: C 43.82, H 4.73, N 8.42%

Synthesis of (^{iPr}N3)Ni(PhAc)Br (**4**)

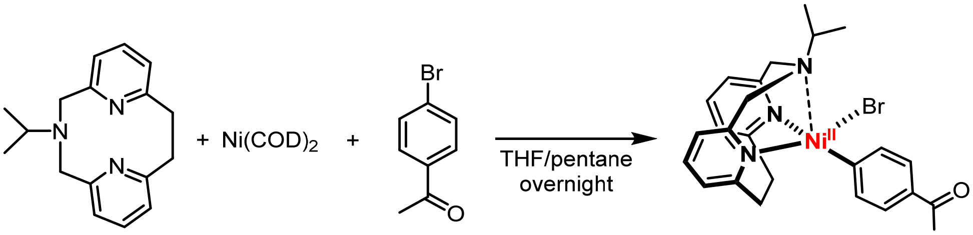

Inside of glovebox, $Ni(COD)_2$ (48 mg, 0.17 mmol), 4-bromoacetophenone (35 mg, mmol) and ^{iPr}N3 (47 mg, 0.18 mmol) were dissolved in 3 mL of THF at room temperature. After 10 min, 2 mL of Pentane was added. The mixture was stirred for overnight and brick red precipitate was collected and dried under vacuum. Yield: 52 mg, 58%. ^{1}H NMR (500 MHz, CD_2Cl_2, –40 °C) δ 8.01 – 7.96 (m, 1H), 7.36 (d, J = 6.9 Hz, 1H), 7.30 (d, J = 8.0 Hz, 1H), 7.25 (t, J = 7.5 Hz, 1H), 7.13 (d, J = 7.9 Hz, 1H), 6.99 (d, J = 7.5 Hz, 1H), 6.93 (d, J = 8.0 Hz, 1H), 6.79 (d, J = 7.7 Hz, 1H), 6.34 (s, 1H), 5.96 (s, 1H), 5.05 (d, J = 16.6 Hz, 1H), 4.51 (d, J = 12.8 Hz, 2H), 4.39 (t, J = 16.1 Hz, 2H), 3.94 (d, J = 14.1 Hz, 1H), 3.86 (d, J = 12.5 Hz, 1H), 2.39 (s, 3H), 2.06 (d, J = 5.6

Hz, 3H), 1.54 (d, J = 5.7 Hz, 3H). NMR spectrum exhibits peak broadening, might be due to the formation of paramagnetic impurities and/or flexible axial N arm. This complex is unstable in solution at LT (–40 °C) as over time and slowly becomes green (^{iPr}N3)NiBr$_2$ complex, but stable as a solid in room temperature. Elemental analysis could not be obtained due to the facile oxidation of the complex or difficulty of purification process (possible impurities: Ni black or decomposed ^{iPr}N3NiBr$_2$) due to the instability in solution.

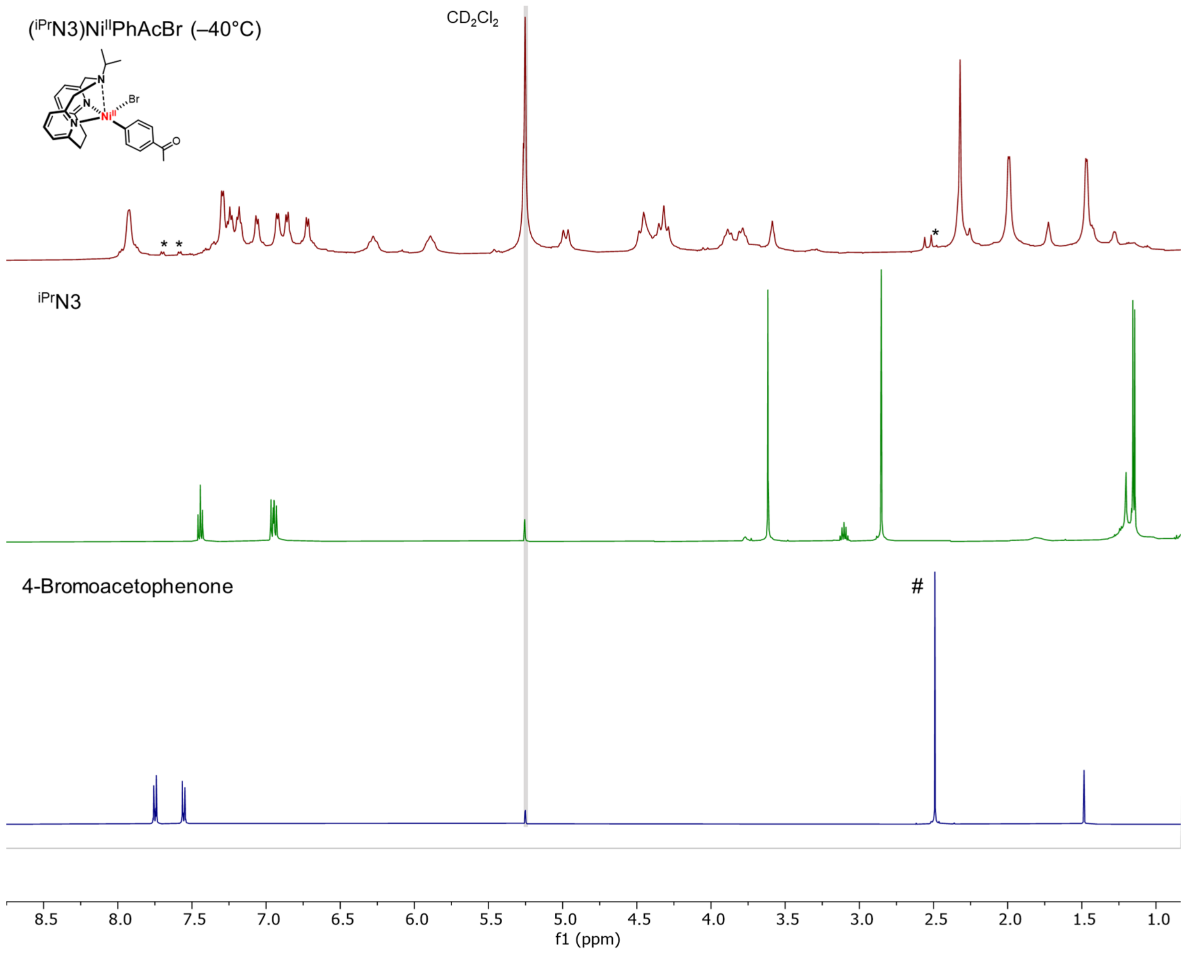

Supplementary Figure 2. Preparation of (^{iPr}N3)Ni^{II}(PhAc)Br (**4**). The ^{1}H NMR spectra in CD$_2$Cl$_2$ show shifts of ^{iPr}N3 ligand and 4-bromoacetophenone peaks upon coordination to the Ni center. Residual peaks for 4-bromoacetophenone (*) and H$_2$O (#) are marked accordingly (see Figure S11 for peak integration.)

Synthesis of [(^{iPr}N3)Ni^{III}(PhAc)(Br)]$^+$ (**5**)

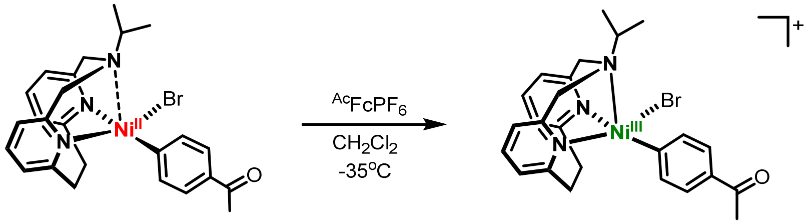

10 mL dichloromethane solution of one equivalent acetylferrocenium hexafluorophosphate (^{Ac}FcPF$_6$, 22 mg, 0.059 mmol) was added to solids of (^{iPr}N3)Ni(PhAc)Br (31 mg, 0.059 mmol) at –35 °C and the resulting solution was stirred for 20 min. The volume of the solution was reduced to 5mL and 10 mL of diethyl ether was added to precipitate the solids. The pink precipitated was filtered and washed with diethyl ether, and dried under vacuum. Yield: 32 mg, 81%. Evans method (CH_2Cl_2): μ_{eff} = 1.98 μ_b. Elemental analysis: Found: C 43.35, H 4.31, N 7.06%; calculated $C_{25}H_{28}BrF_6N_3NiOP$: C 44.81, H 4.21, N 6.27% Several attempts to obtain elemental analysis data proved unsuccessful, likely due to its decomposition during handling. Although these results are slightly outside the range viewed as establishing analytical purity, they are provided to illustrate the best values obtained to date.

1.3 NMR spectra

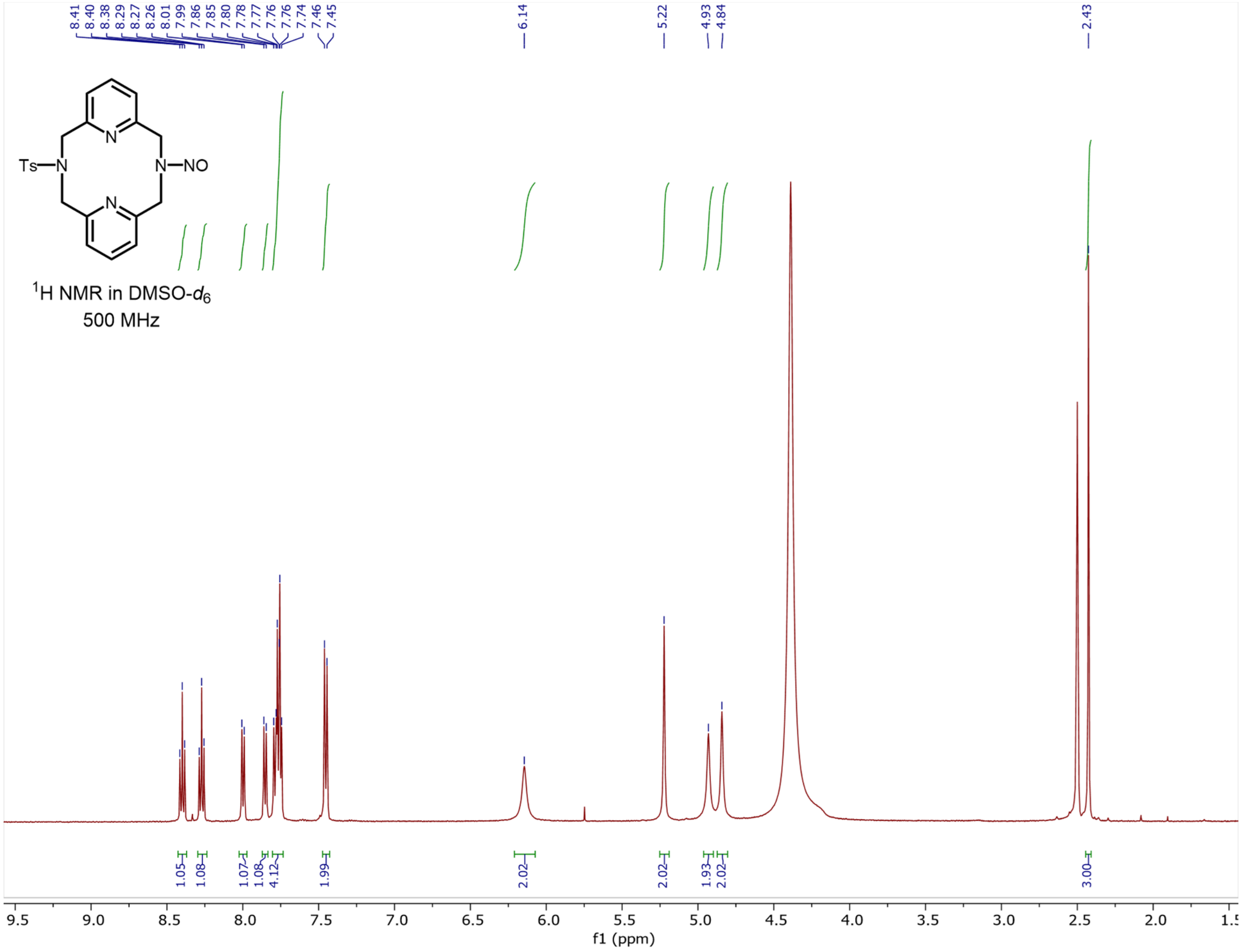

Supplementary Figure 3. ^{1}H NMR spectrum of ^{TsNO}N4, recorded at 500 MHz.

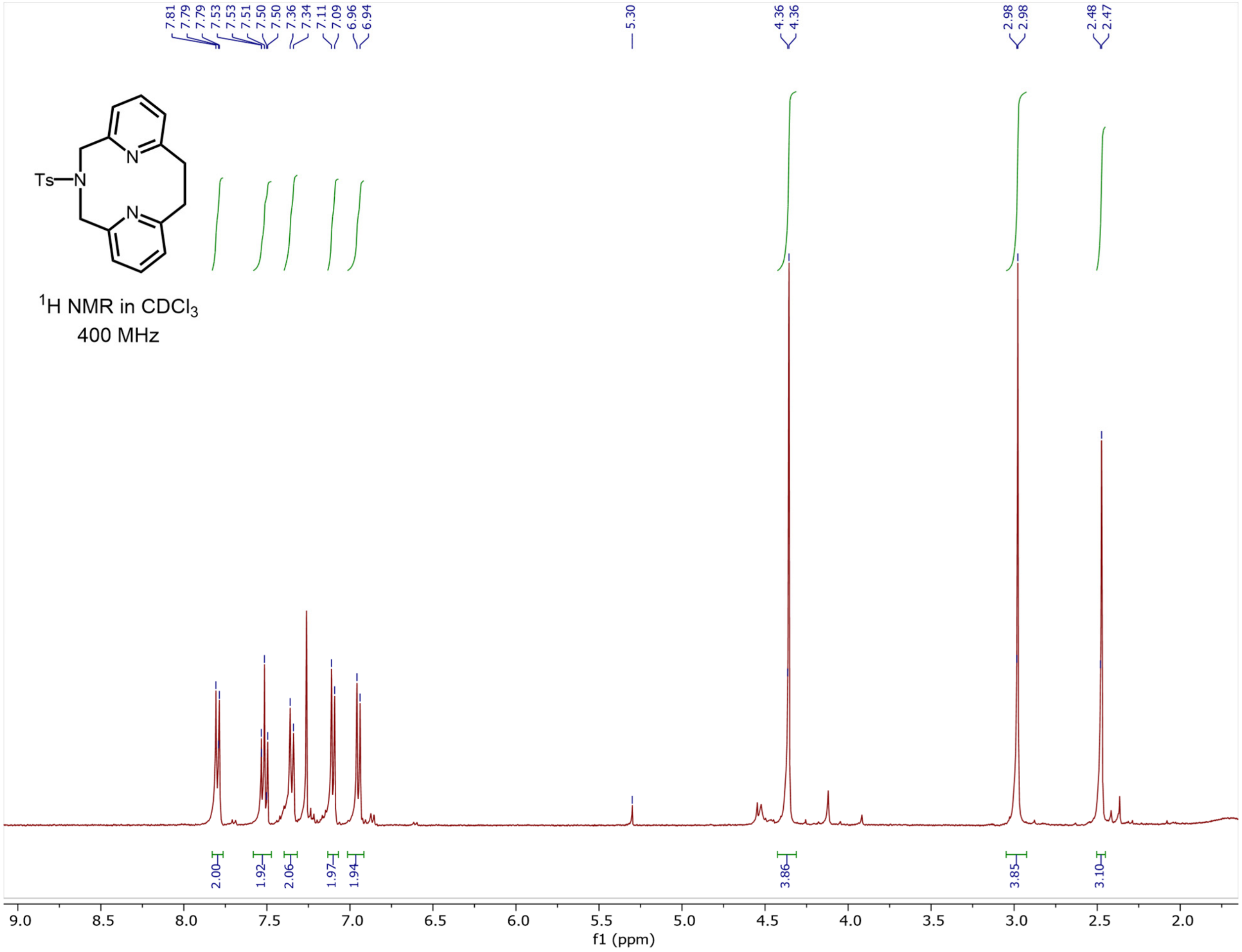

Supplementary Figure 4. ^{1}H NMR spectrum of ^{Ts}N3, recorded at 500 MHz.

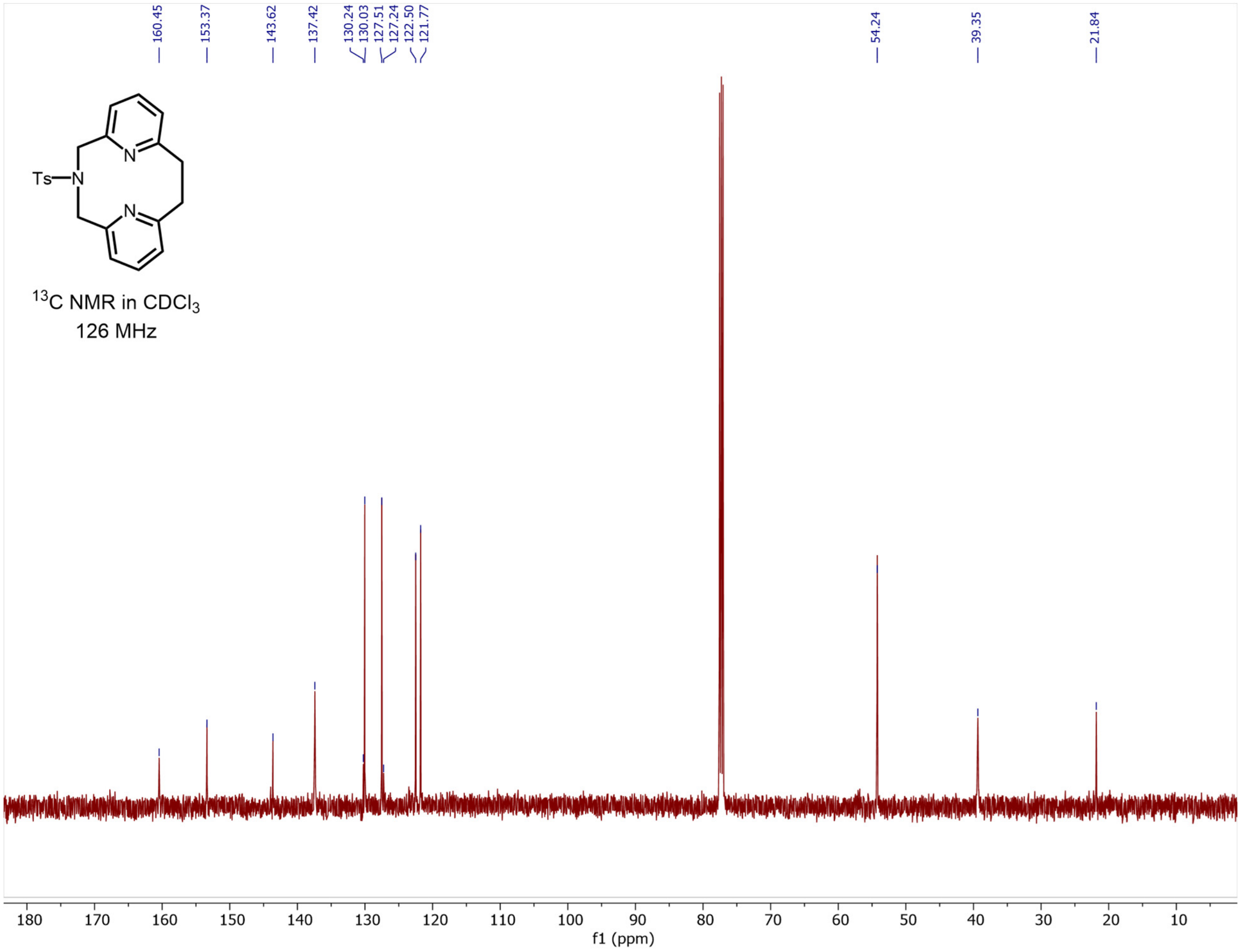

Supplementary Figure 5. ^{13}C NMR spectrum of ^{Ts}N3, recorded at 126 MHz.

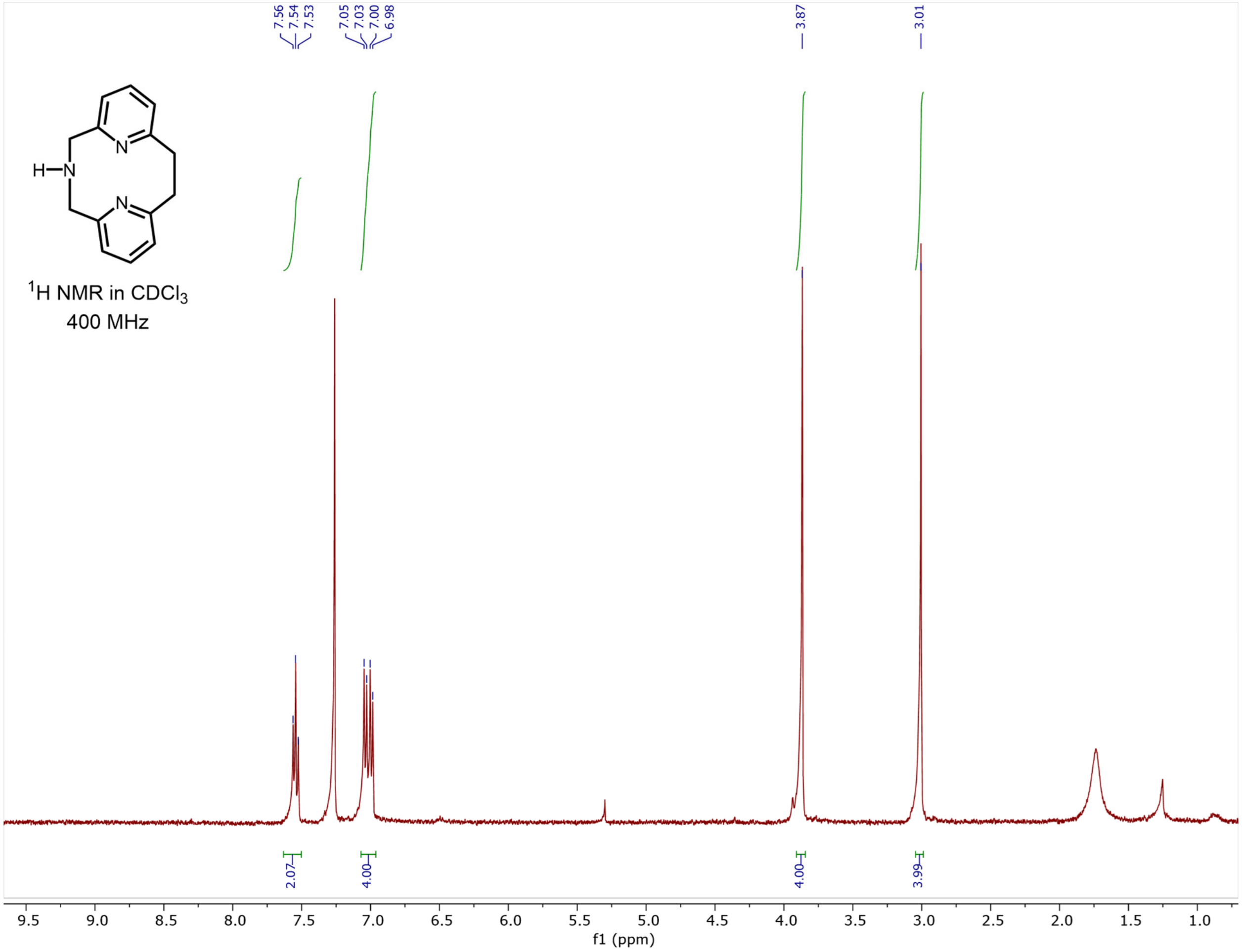

Supplementary Figure 6. ^{1}H NMR spectrum of ^{H}N3, recorded at 500 MHz.

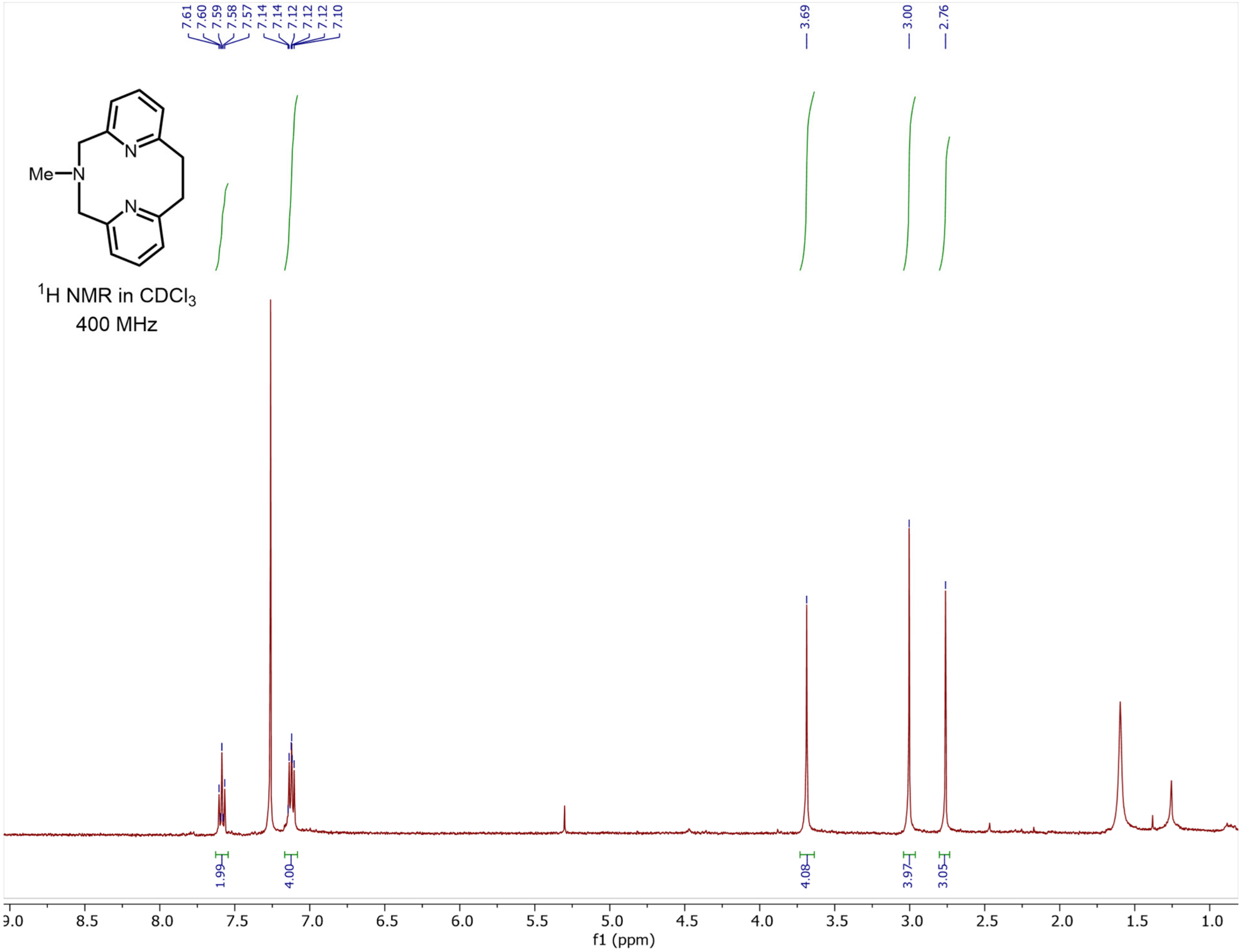

Supplementary Figure 7. ^{1}H NMR spectrum of $^{Me}N3$, recorded at 500 MHz.

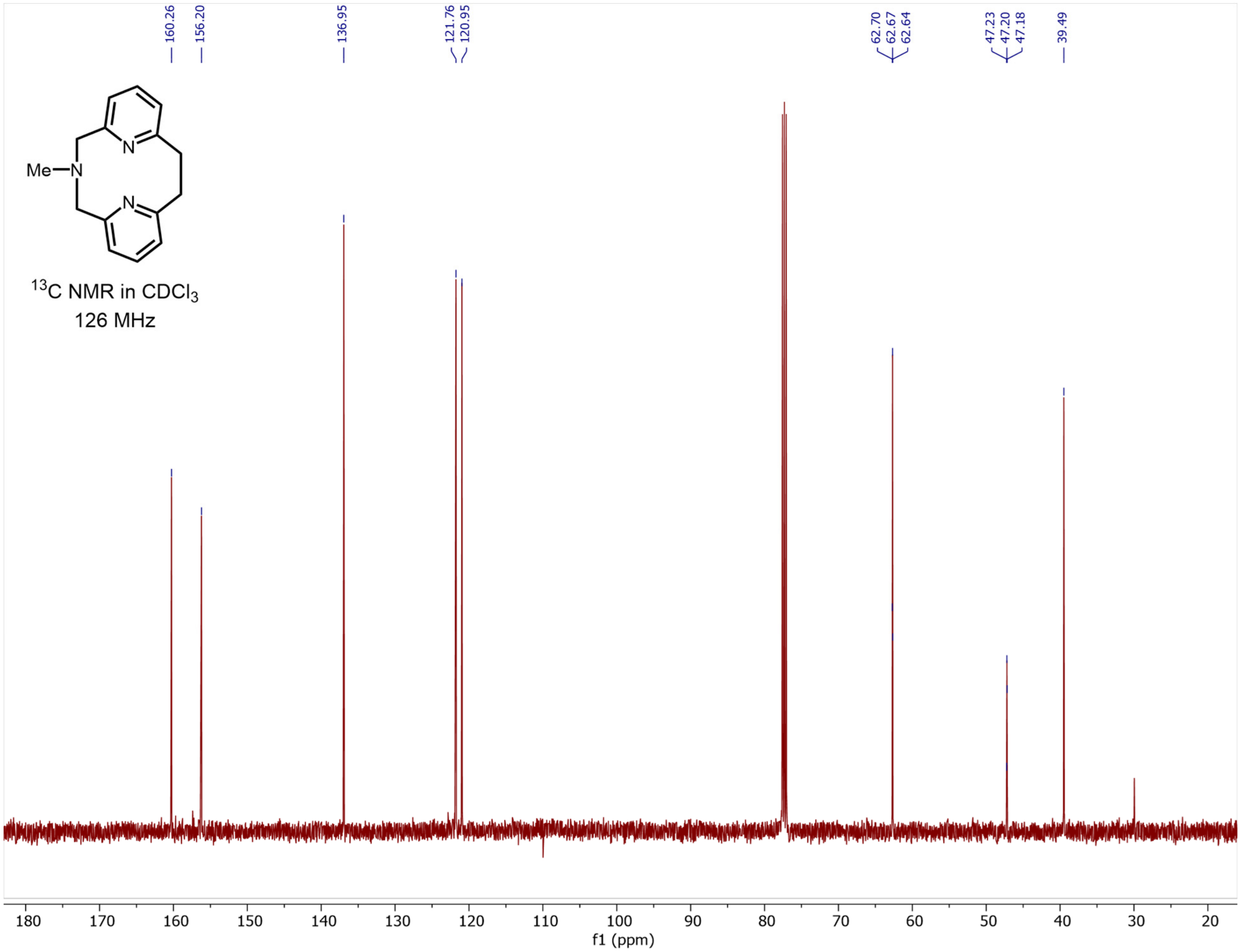

Supplementary Figure 8. ^{13}C NMR spectrum of ^{Me}N3, recorded at 126 MHz.

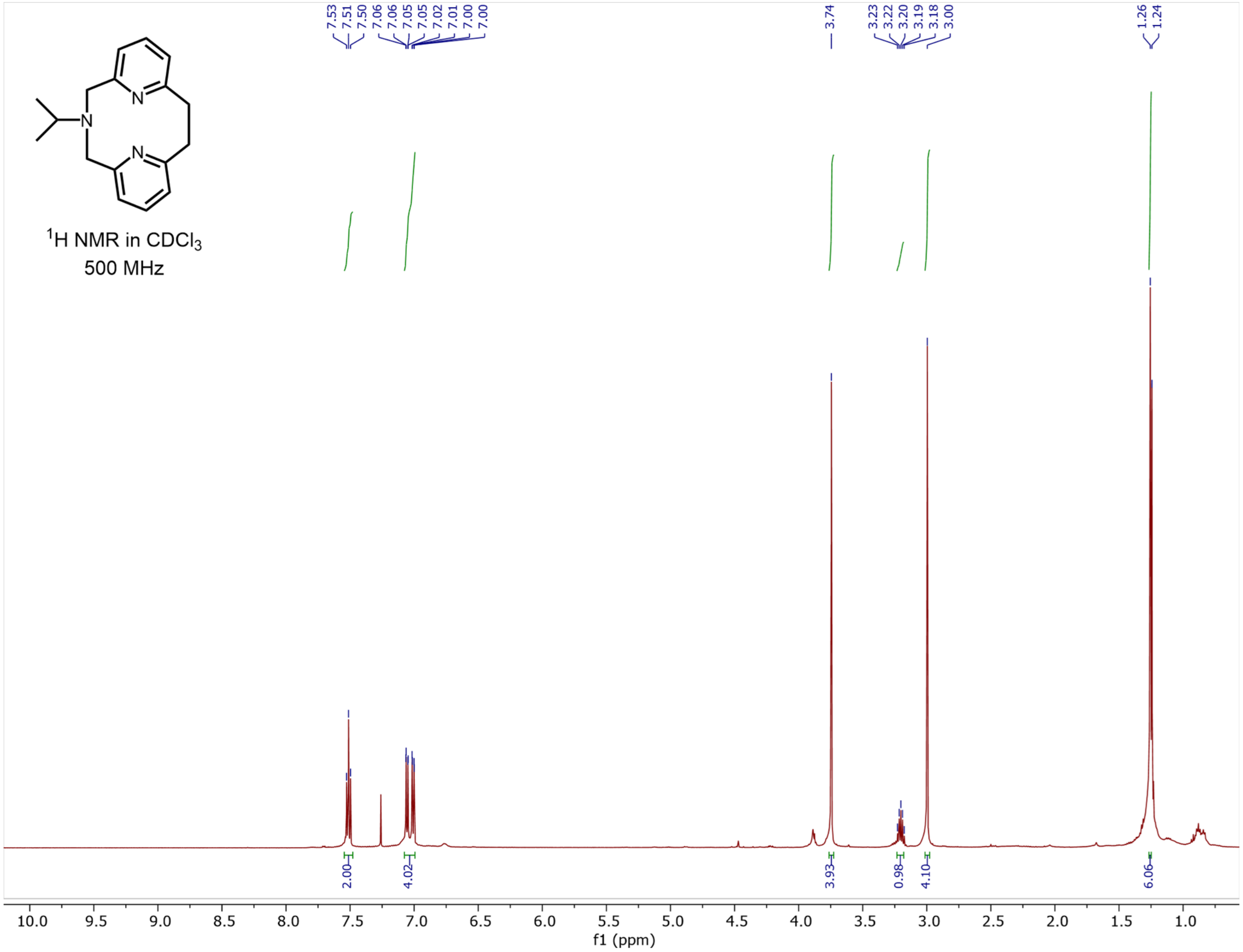

Supplementary Figure 9. ^{1}H NMR spectrum of ^{iPr}N3, recorded at 500 MHz.

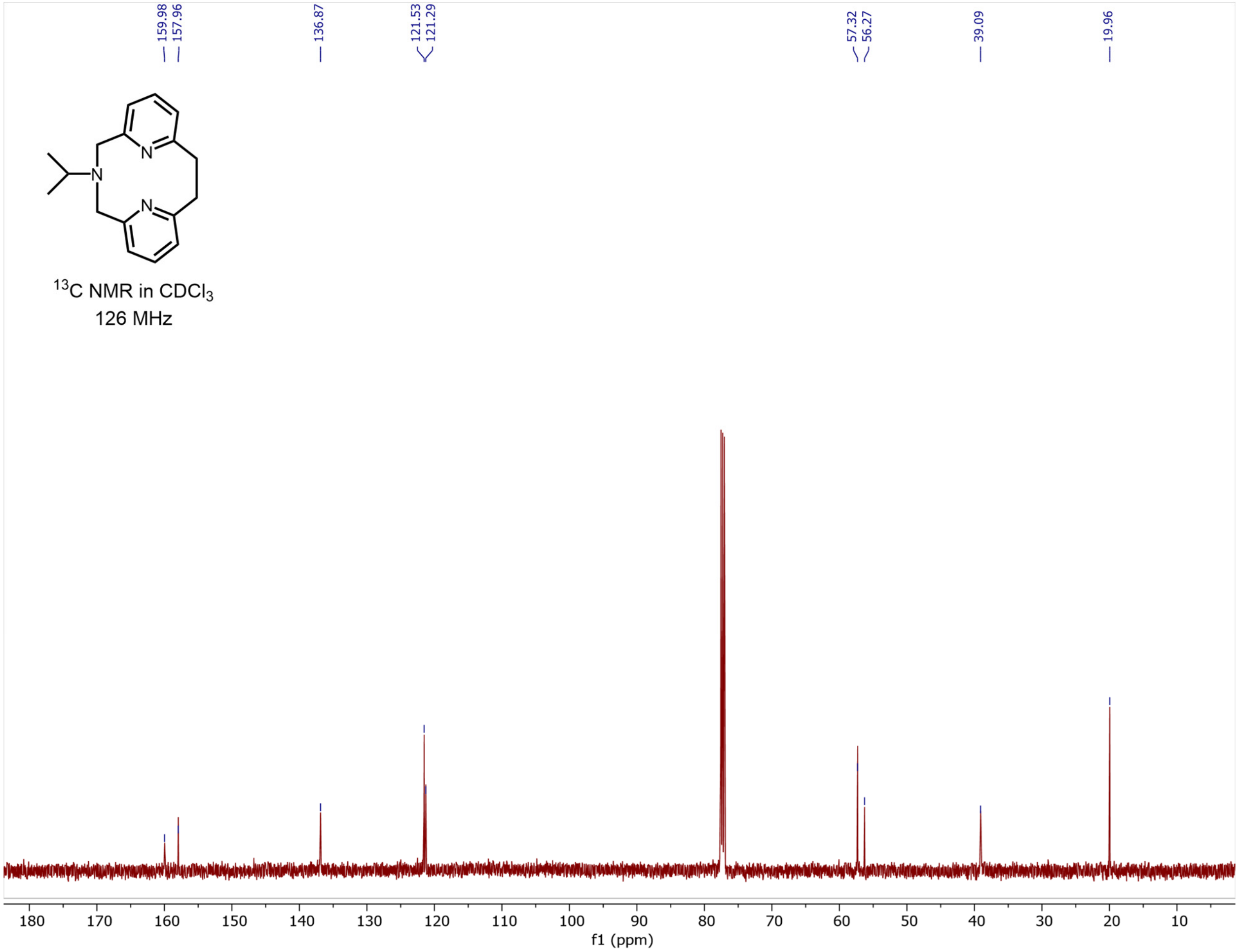

Supplementary Figure 10. ^{13}C NMR spectrum of ^{iPr}N3, recorded at 126 MHz.

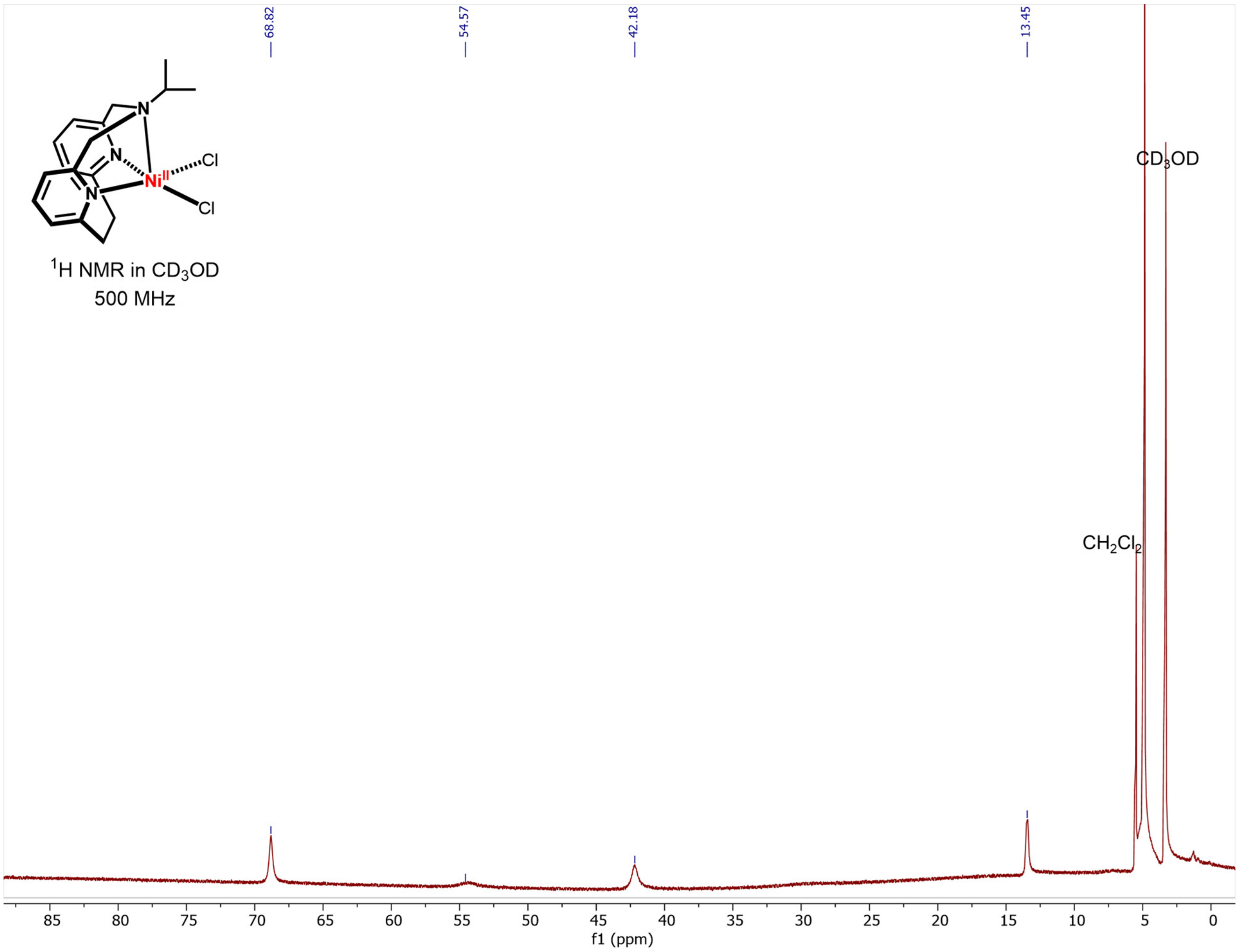

Supplementary Figure 11. ^{1}H NMR spectrum of (^{iPr}N3)NiCl$_2$ (**1b**) recorded at 500 MHz. The ^{1}H NMR spectrum of (^{Me}N3)NiCl$_2$ (**1a**) could not be obtained due its limited solubility.

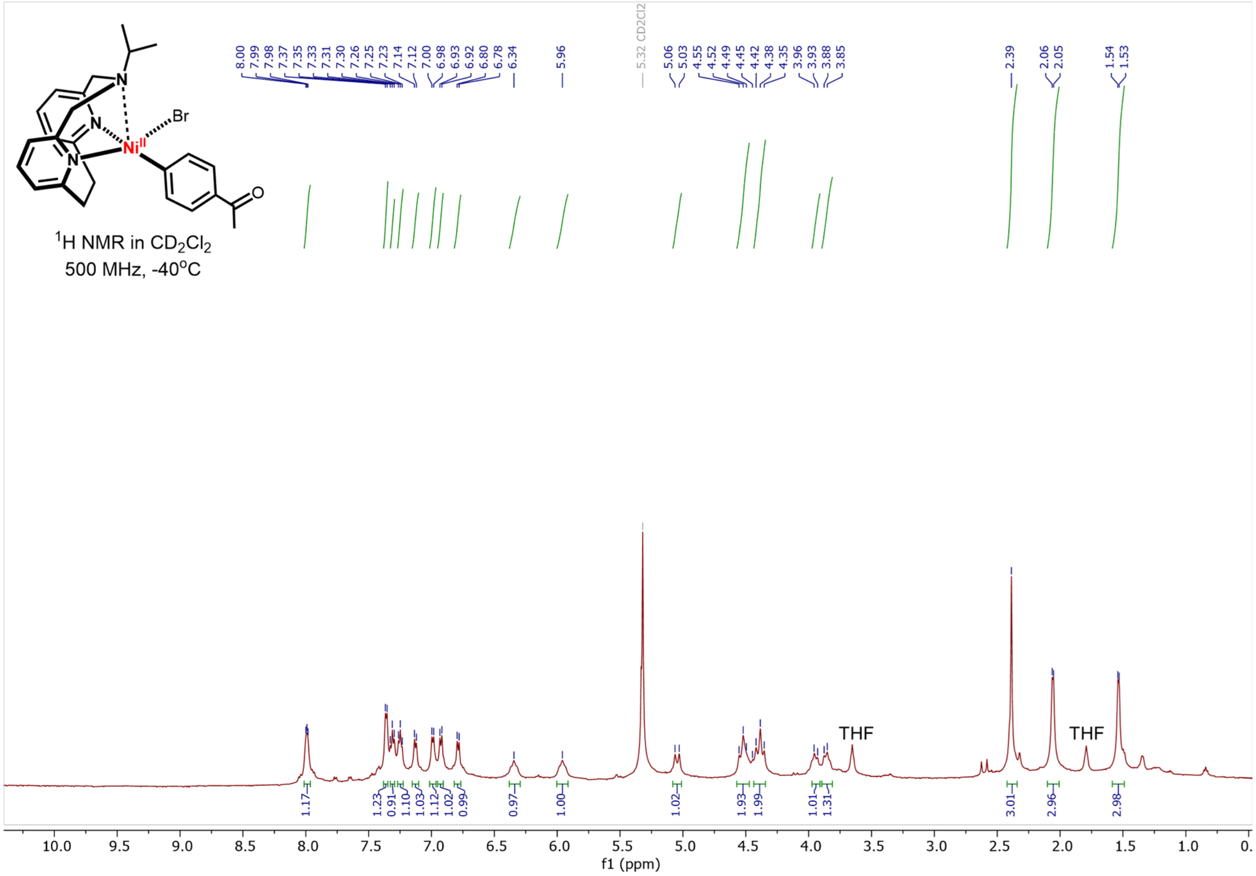

Supplementary Figure 12. ^{1}H NMR spectrum of (^{iPr}N3)Ni(PhAc)Br (**4**), recorded at 500 MHz at –40 °C.

1.4 Cyclic voltammograms of Ni complexes

Supplementary Table 1. Summary of Electrochemical Data[a]

	E^{ox} (V)	E^{red} (V)
(^{Me}N3)$NiCl_2$ (**1a**)	0.71[c], 1.12[d]	–1.20[d], –1.60[c]
(^{iPr}N3)$NiCl_2$ (**1b**)	0.71[c], 1.20[d]	–1.22[d], –1.72[c]
(dtbbpy)$NiCl_2$	0.56[c], 1.15[d]	–1.37[d], –1.72[c]
(^{iPr}N3)Ni(PhAc)Br (**4**)[b]	–0.40[c], 0.46[d], 0.69[c]	–1.89[d]

[a] Measured in 0.1 M $TBAPF_6$ acetonitrile solution with a scan rate of 0.1 V/s. All potentials are reported relative to Fc^+/Fc, and irreversible features are quoted as $E_{p,a}$ or $E_{p,c}$ values. [b] Recorded at –40°C in 0.1 M $TBAPF_6$ CH_2Cl_2 solution. [c] Quasi-reversible. [d] Irreversible.

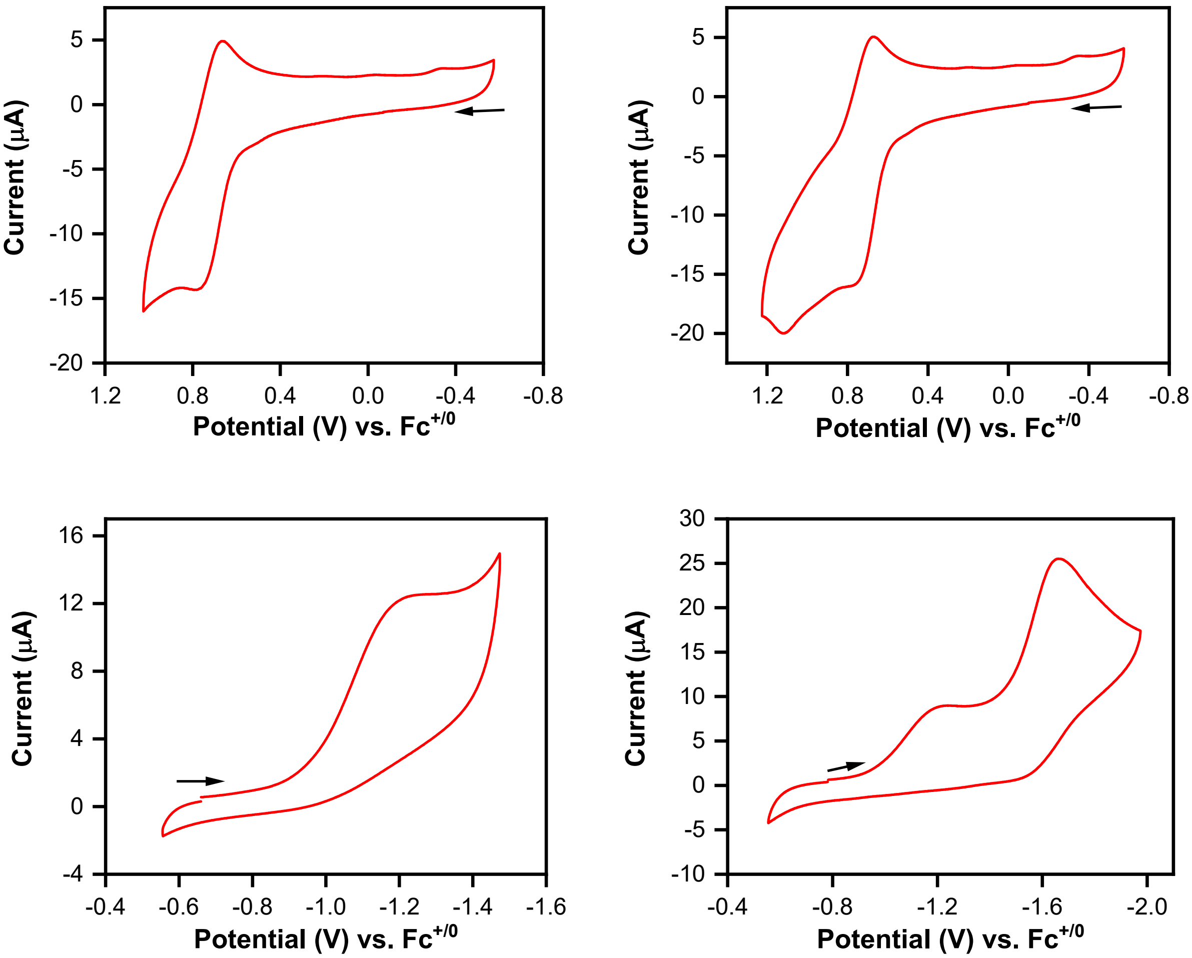

Supplementary Figure 13. Cyclic voltammograms of (^{Me}N3)$NiCl_2$ (**1a**), recorded at room temperature in 0.1 M $TBAPF_6$ MeCN with a scan rate of 0.1 V/s.

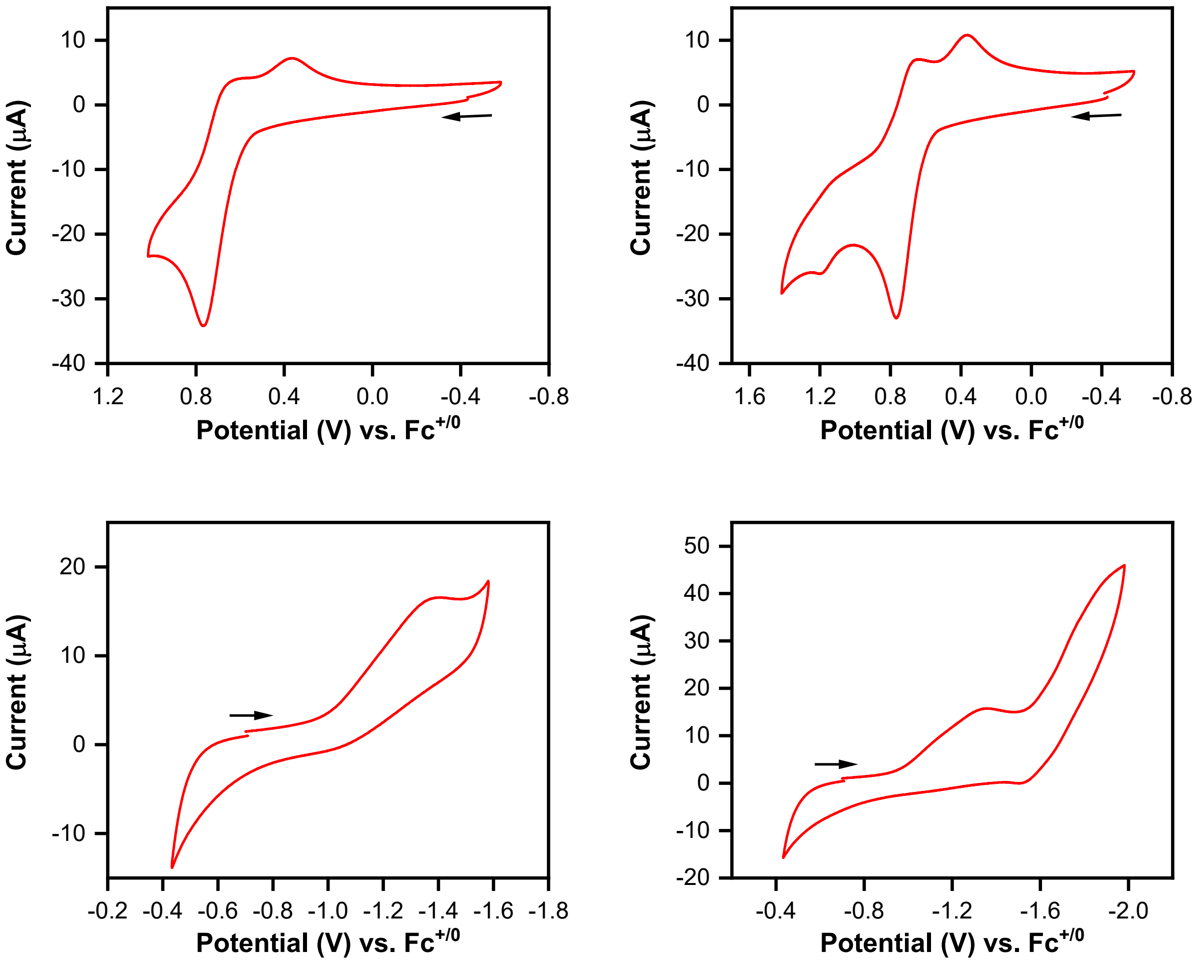

Supplementary Figure 14. Cyclic voltammograms of (^{iPr}N3)$NiCl_2$ (**1b**), recorded at room temperature in 0.1 M $TBAPF_6$ MeCN with a scan rate of 0.1 V/s.

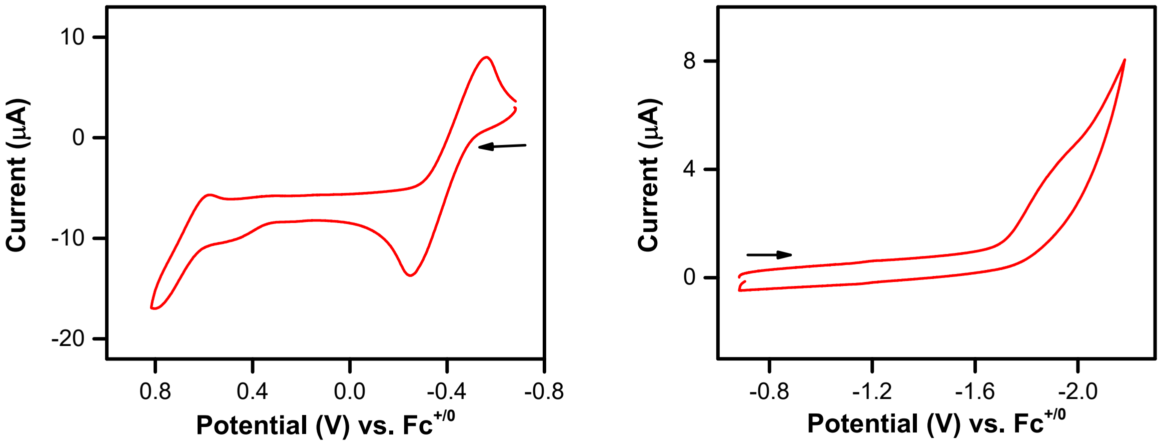

Supplementary Figure 15. Cyclic voltammograms of (^{iPr}N3)Ni(PhAc)Br (**4**), recorded at –40°C in 0.1 M $TBAPF_6$ CH_2Cl_2 with a scan rate of 0.1 V/s.

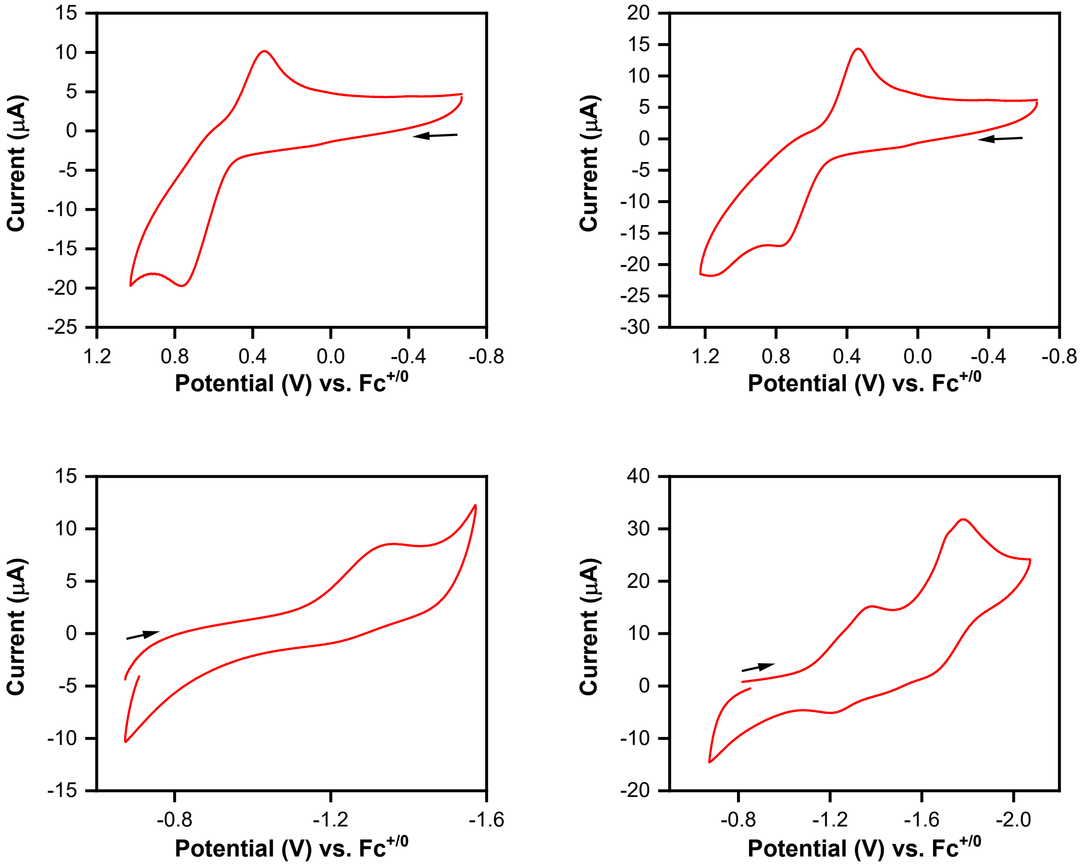

Supplementary Figure 16. Cyclic voltammograms of (dtbbpy)$NiCl_2$, recorded at room temperature in 0.1 M $TBAPF_6$ MeCN with a scan rate of 0.1 V/s.

1.5 UV-vis absorption spectra

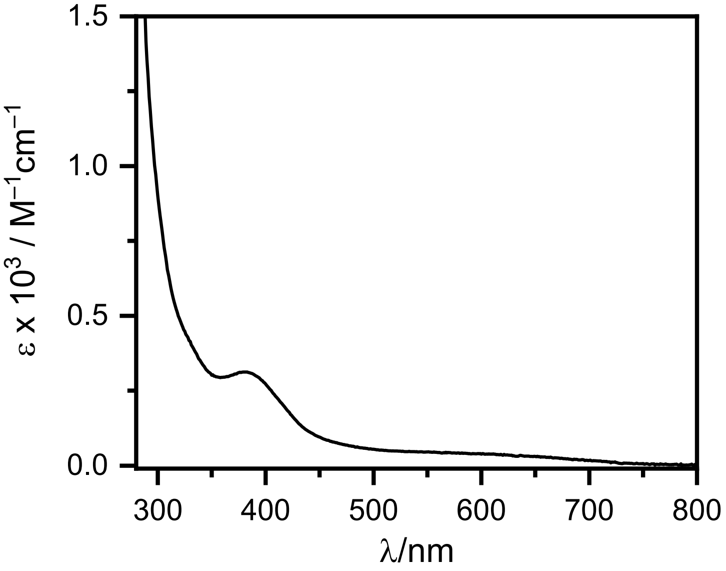

Supplementary Figure 17. UV-vis absorption spectrum of (MeN3)NiCl2 (**1a**) in CH2Cl2

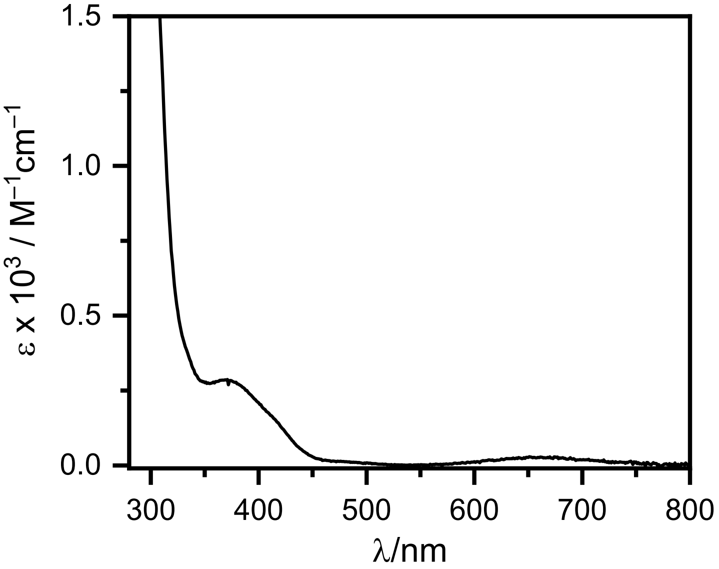

Supplementary Figure 18. UV-vis absorption spectrum of (iPrN3)NiCl2 (**1b**) in CH2Cl2

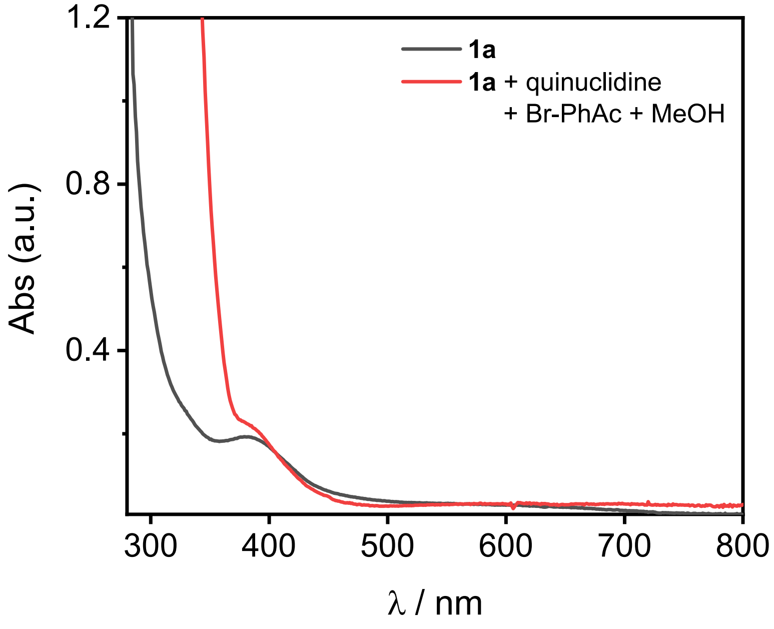

Supplementary Figure 19. UV-vis spectrum of ([Me]N3)$NiCl_2$ (**1a**) in the presence of quinuclidine, 4-bromoacetophenone and methanol (a molar ratio of Ni:Ar-Br:quinuclidine:MeOH = 1:50:55:200 was maintained, corresponding to the ratio in the photocatalytic reaction).

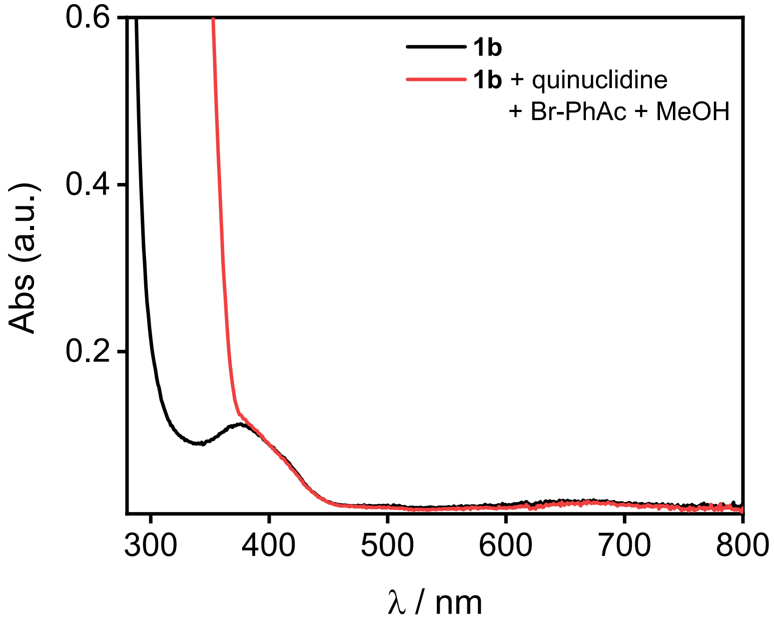

Supplementary Figure 20. UV-vis spectrum of ([iPr]N3)$NiCl_2$ (**1b**) in the presence of quinuclidine, 4-bromoacetophenone and methanol (a molar ratio of Ni:Ar-Br:quinuclidine:MeOH = 1:50:55:200 was maintained, corresponding to the ratio in the photocatalytic reaction).

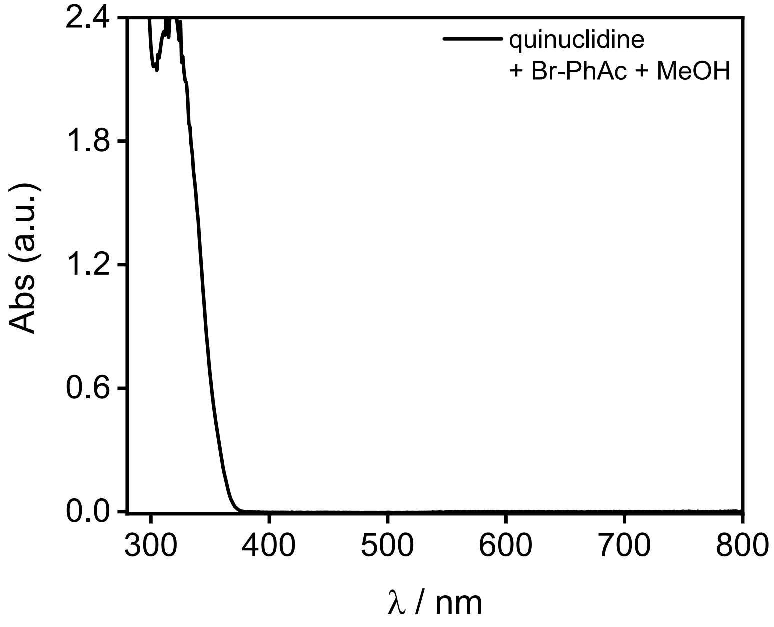

Supplementary Figure 21. UV-vis spectrum of quinuclidine, 4-bromoacetophenone, and methanol.

1.6 Nickel catalyzed C–O coupling experiments

Reaction optimization

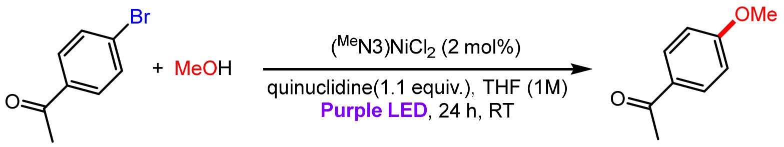

General procedure for the etherification reaction: In a nitrogen-filled glovebox, 4-bromoacetophenone (0.4 mmol, 1.0 equiv), quinuclidine (0.44 mmol, 1.1 equiv), MeOH (1.6 mmol, 4 equiv), ^{Me}N3NiCl$_2$ (2 mol %), and a magnetic stir bar were added into 0.4 mL of THF in a vial. The vial was irradiated with one Kessil purple LED (λ_{max} = 390 nm, max 52W) under fan cooling. After 24 h, 1,3-benzodioxole (0.4 mmol) was added to the reaction mixture. The residue was analyzed by ^{1}H NMR in $CDCl_3$ and GC-FID to give the yield of the reaction. The authentic product 4-Methoxyacetophenone was purchased from AK scientific and used to determine retention time and a response factor for GC-FID quantification.

Reaction optimization tables and control experiments

Supplementary Table 2. Control experiment

Entry	Condition	Ni loading	Yield (%)
**1**	1M, no light[a]	2%	0
**2**	1M	0%	0
**3**	1M, no Base	2%	0

[a] The dark entry was covered with foil and placed under purple LED.

Supplementary Table 3. Concentration dependence

Entry	Concentration	Ni loading	Yield (%)
**1**	0.5M	1%	77
**2**	1M	1%	94
**3**	2M	1%	92
**3**	0.25M	2%	63
**4**	0.5M	2%	72
**5**	1M	2%	95

Supplementary Table 4. Ni catalyst loading

Entry	Ni catalyst loading (1M)	Yield (%)
**1**	0.2 mol%	90. ± 1[a]
**2**	0.65 mol%	90
**3**	1 mol%	94
**4**	2 mol%	95 ± 1[a]

[a] The average product yield from three independent experiments

Supplementary Table 5. Solvent screening

Entry	Solvent	Ni loading	Yield (%)
**1**	THF	2%	95 ± 1[a]
**2**	MeCN	2%	20
**3**	Toluene	2%	59

[a] The average product yield from three independent experiments

Supplementary Table 6. Base screening

Entry	Base	Yield (%)
**1**	Quinuclidine	95 ± 1[a]
**2**	DABCO	77
**3**	DABCO, 36 h	97
**4**	DBU	9.0
**5**	K_2CO_3	0
**6**	KH_2PO_4	0
**7**	K_3PO_4	0
**8**	10% quinuclidine + 1 equiv K_2CO_3	0.33 (trace)

[a] The average product yield from three independent experiments

Supplementary Table 7. Alcohol scope[a]

Entry	Alcohol	Yield (%)
**1**	MeOH	95 ± 1[b]
**2**	1-hexanol	90
**3**	Benzyl alcohol	86

[a] General reaction condition (1M in THF, Ni catalyst 2wt%) was used.

[b] The average product yield from three independent experiments.

Supplementary Table 8. Ni catalyst[a]

Entry	Ni source	Yield (%)
**1**	$(^{Me}N3)NiCl_2$	95 ± 1[b]
**2**	$(^{Me}N3)NiBr_2$	90
**3**	$(^{iPr}N3)NiCl_2$	82
**4**	$^{Me}N3+Ni(DME)Cl_2$	77
**5**	$[(^{iPr}N3)Ni^{III}(PhAc)Br]PF_6$	31
**6**	$(^{Me}N4)NiCl_2$	2
**7**	$(iPr_3TACN)NiCl_2$	51

[a] General reaction condition (1M in THF, Ni catalyst 2wt%) was used.

[b] The average product yield from three independent experiments.

1.7 Light on/off experiments

General procedure. In a N_2-filled glovebox, 4-bromoacetophenone (0.4 mmol, 1.0 equiv), quinuclidine (0.44 mmol, 1.1 equiv), MeOH (1.6 mmol, 4 equiv), $^{Me}N3NiCl_2$ (2 mol%), and a magnetic stir bar were added into 0.4 mL THF in a vial. Two separate samples were prepared and irradiated with one Kessil purple LED (λ_{max} = 390 nm, max 52W) under fan cooling. One sample was irradiated continuously for 20 h (entry 1) while the other sample was irradiated for 15 min (light on) then stirred for 45 min (light off) using a PR controller (part number: KSPRCT01), and this light on/off process was performed for 20 h, resulting in total irradiation for 5 h (entry 2). After 20 h, 1,3-benzodioxole (0.4 mmol) was added to the reaction mixture. For comparison, one sample was prepared and irradiated with purple LED continuously for 5 h (entry 3). The reaction mixture was analyzed by GC-FID to give the yield of the reaction.

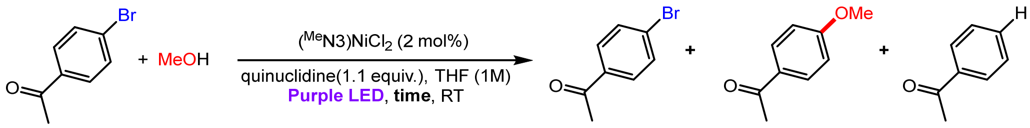

Supplementary Table 9. Light on/off experiments

Entry	Conditions		Ar-Br	Ar-OMe	Ar-H
**1**	Light on for 20 h	trial 1	0%	89%	4%
		trial 2	10%	81%	3%
		trial 3	4%	89%	4%
		average	5±4%	86±4%	3.6±0.5%
**2**	Light 15 min on, 45 min off, for 20 h (total irradiation time 5 h)	trial 1	14%	82%	1%
		trial 2	23%	72%	1%
		trial 3	16%	81%	2%
		average	18±4%	78±4%	1.3±0.5%
**3**	Light on for 5 h	trial 1	31%	62%	0%
		trial 2	43%	56%	1%
		trial 3	32%	63%	1%
		average	35±5%	60±3%	0.7±0.5%

Comparison of entries 1, 2, and 3 reveals that light on/off conditions (entry 2) generated a comparable amount of C–O coupled product to the 20 h irradiation conditions (entry 3). Furthermore, the yield of C–O coupled product obtained in entry 2 is ~20% higher and the consumed amount of Ar-Br substrate is also higher compared to those from entry 3, indicating that catalysis can continue while the light is off and thus supporting a self-sustained catalytic cycle.

Although a self-sustained Ni^{I}/Ni^{III} cycle is operative, considering that a comproportionation reaction between Ni^{I} and Ni^{II} can generate off-cycle Ni^{II} species during catalysis, as reported by Nocera *et al.,*[4] intermittent irradiation is required to regenerate the Ni^{I} species necessary for catalytic turnover (see section 1.10 for further details).

1.8 Quantum yield measurements

Reactions were carried out with one Kessil lamp model PR160 purple LED (λ_{max} = 390 nm, max 52W) at 100% light intensity. Fresh potassium ferrioxalate was prepared following the literature procedure as a standard actinometer under the dark.[5] The developer solution was prepared using 22.5 g of sodium acetate trihydrate and 1 g of 1,10-phenanthroline in 100 mL of 0.5 M sulfuric acid and it was stored in the dark.

A 1 cm × 0.5 cm quartz cuvette was charged with 0.8 mL of 0.15 M potassium ferrioxalate solution in 0.05 M sulfuric acid. Two sides of the cuvettes were coated with black electrical tape to ensure a minimum pathway of the light (1 cm). While stirring, the solution was irradiated with a purple LED at room temperature under a fan cooling. After irradiation for 1 min, 2 min, and 4 min, the 1 μL aliquot at each time point was added to 5 mL of a developer solution, and the vial was wrapped in a foil. A blank sample was prepared by adding 1 μL of the non-irradiated potassium ferrioxalate solution into 5 mL of developer solution. All solutions were kept in the dark for 1 h before transferring into the cuvettes and measuring the UV-vis absorption. The absorbance at 510 nm (ε = 11,100 M^{-1} cm^{-1})[5] was measured, and the moles of ferrous ion produced by ferrioxalate photo-degradation was determined by the following equation.

$$moles\ Fe^{2+} = \frac{V_1\, V_3\, \Delta A_{510nm}}{V_2\, l\, \varepsilon_{510nm}} \quad (1)$$

V_1 = total volume of irradiated solution (0.8 mL)

V_2 = volume of aliquot taken from V1 (1 μL)

V_3 = the volume that V_2 is diluted into (5 mL)

ΔA_{510nm} = difference in absorbance at 510 nm between blank and irradiated sample

l = pathlength (1 cm)

ε_{510nm} = extinction coefficient of $Fe(phen)_3^{2+}$ at 510 nm (11,100 M^{-1} cm^{-1})

From the above equation (1), the moles of Fe(II) photoproduct was calculated to be 4.44 $\times 10^{-5}$.

The photon flux can be determined by using the following equation (2) and quantum yield of ferrioxalate decomposition (Φ =1.13 at 392 nm):[6]

$$photon\ flux = \frac{moles\ of\ Fe^{2+}}{\Phi \times t \times F} \quad (2)$$

Φ =1.13

t = irradiated time (seconds)

F = mean fraction of light absorbed by the ferrioxalate solution (F ≈ 1 at 365–405 nm)[7]

The calculated photon flux in einsteins sec^{-1} $sample^{-1}$ is 3.274×10^{-7} (for a 0.8 mL irradiated sample solution).

The quantum yield (QY) of the photochemical reaction can be determined by the following equation (3):

$$\Phi = \frac{Rate\ of\ product\ formation\ (moles/sec)}{Total\ photon\ flux\ (moles/sec)\times(1-10^{-A(390nm)})} \quad (3)$$

Fraction factor $f = 1 - 10^{-A(390nm)}$, A(390) = absorbance at 390 nm of Ni complex in the solution, which is calculated from the absorption coefficient and concentration (A(390nm) = ε_{390nm} × concentration of Ni complex). Considering the low concentration of Ni complex in the reaction mixture (due to the limited solubility in THF), f = 0.4087 was used.

Therefore

$$\Phi = \frac{Rate\ of\ product\ formation\ (moles/sec)}{(3.274\times10^{-7})\times(0.4087)} = \frac{Rate\ of\ product\ formation\ (moles/sec)}{1.338\times10^{-7}} \quad (4)$$

It means that for QY = 1 (100%), 1.338×10^{-7} moles of the product should be obtained per second, indicating a fast reaction rate. When the reaction mixture of 0.8 mL (0.8 mmol of 4-bromoacetophenone is used) is irradiated, the C–O coupled product needs to be obtained 100% in 5979 sec =1.66 hr, theoretically.

However, in our reaction conditions the completion of the reaction takes longer than 5 h, indicating a QY < 1. Considering the average 78% yield obtained following the 5 h irradiation in light on/off experiments, the calculated QY is 0.26 (26%), while the average 60% yield obtained for the continuous 5 h irradiation resulted in the QY of 0.19 (19%). The lower QY is likely due to the less efficient absorption of light in the heterogeneous reaction mixture that contains insoluble material

and could prevent the accurate determination of the number of photons absorbed by Ni in the catalytic condition. In addition, the quantum yield ($\Phi_{Ni} < 0.26$) obtained by actinometry could be significantly overestimated. Given that there are several possible side reactions that would generate off-cycle Ni^{II} species, if $\Phi_{Ni} << 1$, then the dark Ni^{I}/Ni^{III} dark catalytic cycle may not be able to compensate for the deleterious side reactions. Furthermore, it has been reported that the calculated QY could vary during (on the reaction progress), and QY changes from 0.13 to 2.[8] While values of Φ >1 were reported previously for similar photoredox/Ni dual catalysis C–O cross-coupling reactions,[4] a dark Ni^{I}/Ni^{III} cycle seems to be operative since higher yields were obtained in the light on/off experiments than the comparable light-on experiments (Supplementary Table 9).

1.9 ReactIR experiments

General procedure: In a nitrogen-filled glovebox, 4-bromoacetophenone (0.4 mmol, 1.0 equiv), quinuclidine (0.44 mmol, 1.1 equiv), MeOH (1.6 mmol, 4 equiv), $^{Me}N3NiCl_2$ (**1a**, 2 mol %), and a magnetic stir bar were added into 0.4 mL of THF in an oven-dried flask and closed with a septum. Under a positive N_2 flow, the septum was quickly replaced with the ReactIR probe. The reaction flask was irradiated with one Kessil purple LED (λ_{max} = 390 nm, max 52W) under fan cooling. Data acquisition for *in-situ* IR was performed with an interval of 15 s.

Reaction monitoring: To assess reaction progress, the peak of 4-bromoacetophenone (substrate) at 1690 cm^{-1} was tracked due its relatively high intensity. The peak of 4-methoxyacetophenone (product) at 1511 cm^{-1} was also tracked since it is well-separated from the peak of 4-bromoacetophenone. However, a detailed analysis was performed using 4-bromoacetophenone (substrate) consumption trend lines, as the intensity of product IR stretching at 1511 cm^{-1} was relatively small and thus resulted in significant noise and inconclusive analysis. Since switching of LED light on/off affects signal intensity (i.e., turning light off increases signal intensity, resulting in spikes, see Supplementary Figure 24), the reaction mixture without the Ni catalyst (**1a**) was monitored under the same conditions to establish a background trend line to be used to obtain corrected plots (green solid lines in Supplementary Figure 25).

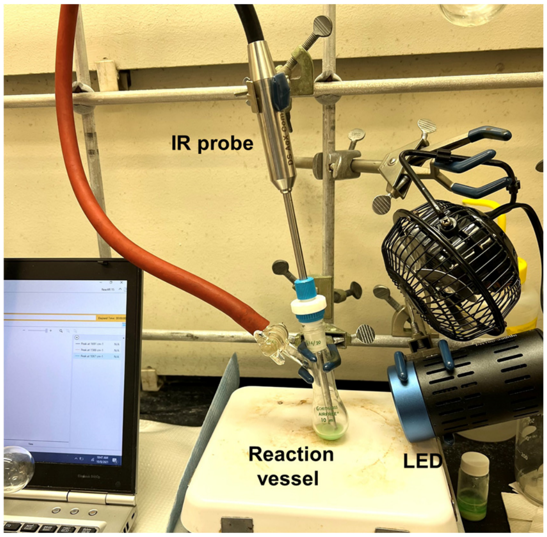

Supplementary Figure 22. ReactIR reaction setup.

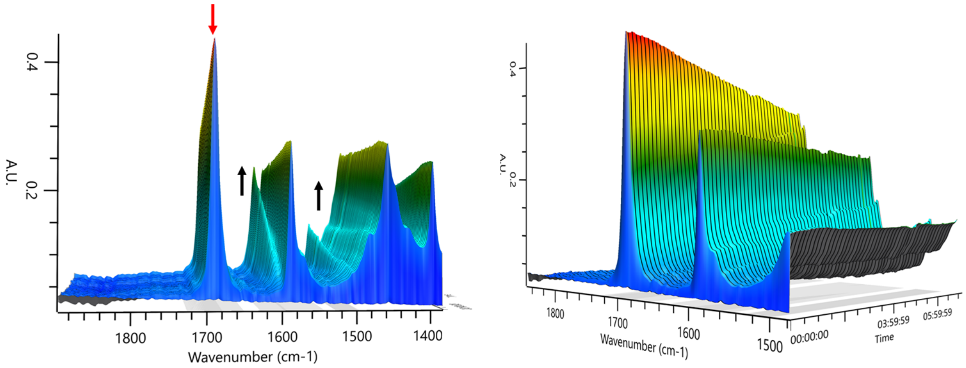

Supplementary Figure 23. ReactIR 3D surface plot of the spectra as a function of time and wavenumber.

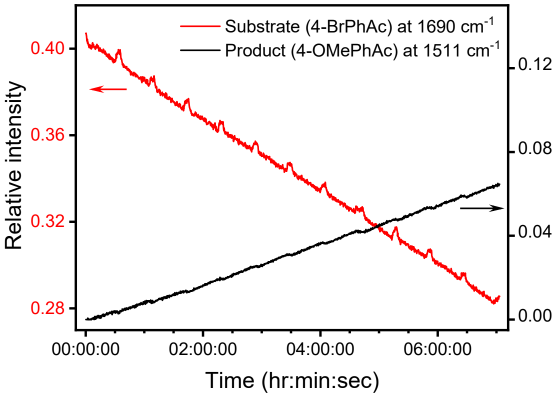

Supplementary Figure 24. Full ReactIR reaction trend lines for 4-bromoacetophenone consumption (solid red line) and 4-methoxyacetophenone formation (solid black line) over 7 h, with light 30 min on/5 min off.

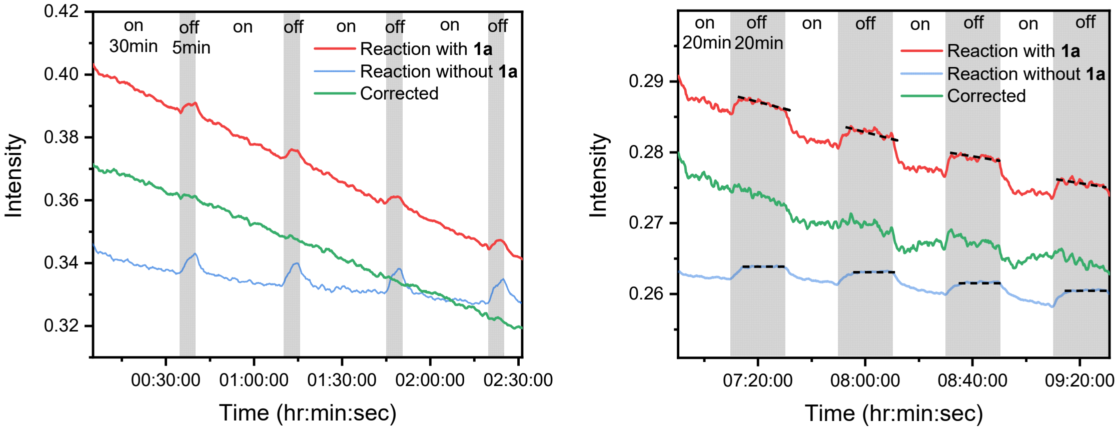

Supplementary Figure 25. ReactIR reaction progress trend lines for 4-bromoacetophenone consumption (at 1690 cm^{-1}) on an enlarged scale. Reaction with light 30 min on/5 min off (left), and 20 min on/20 min off (right). To allow for a clear visualization of all trend lines in one figure, some trend lines were moved up or down on the Y axis by an arbitrary value. Red solid line: reaction with **1a**. Blue solid line: reaction without **1a**.; green solid line: corrected trendline, showing a continuous consumption of the substrate.

Data analysis: As shown in Supplementary Fig. 25, in the 30 min light on/5 min light off experiments a continuous consumption of substrate was observed in the corrected trend line (green solid line), including the light off period. For the 20 min light on/20 min light off experiments the corrected trend line was noisier, and then the slopes corresponding to the substrate consumption between the reaction with and without **1a** during the light-off time were compared. Reaction with **1a** exhibits a noticeable decrease of the signal intensity during light off-time (solid red line, black dash), suggesting the consumption of the substrate. In contrast, in the absence of **1a** there is a negligible signal intensity decrease (solid blue line, black dash). Taken together, these results support a self-sustained Ni^{I}/Ni^{III} dark cycle, although throughout the catalysis a continuous irradiation is likely needed to regenerate the depleted active Ni^{I} species due to the Ni^{I}/Ni^{III} comproportionation process (see pages S37–S41 for further discussion).

1.10 Comproportionation studies

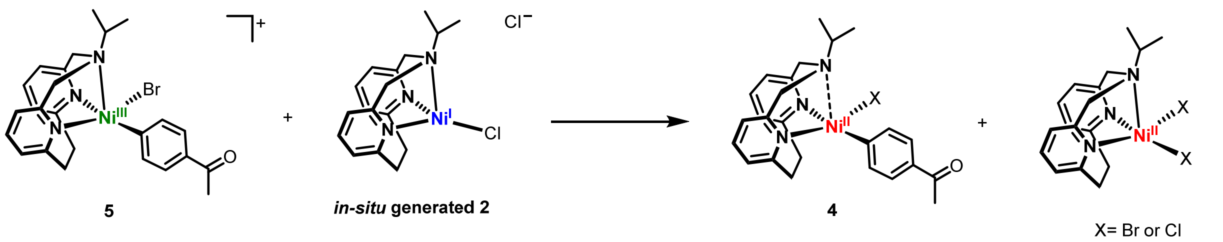

To further assess the Ni^{I} and Ni^{III} deactivation pathway, we attempted to experimentally observe the comproportionation between Ni^{III} and *in situ* generated Ni^{I} through EPR and NMR spectroscopy.

EPR spectroscopy: frequency ≈ 9.42 GHz, power = 0.6325 mW, modulation frequency = 100 kHz, modulation amplitude = 5 G, time constant = 82 ms, linewidth = 10–20 G.

In a N_2-filled glovebox, complex **5** $[(^{iPr}N3)Ni^{III}PhAcBr]PF_6$ (2 mg, 1 equiv) in 0.2 mL of CH_2Cl_2 was added to the EPR tube and the solution was frozen at 77 K. Then 0.4 mL toluene was layered and frozen to separate complex **5** and **2**. On top of the toluene layer, 0.2 mL MeOH solution of complex **1b** $(^{iPr}N3)NiCl_2$ (1.2 mg, 1 equiv) and 0.2 mL toluene solution of $CoCp^*_2$ (1 mg, 1 equiv) was layered, the sample was quickly frozen, and taken outside of the glovebox. The initial EPR spectrum was recorded at 77 K before mixing the sample to confirm the presence of complex **5**. The EPR tube was warmed up to –95 °C for mixing the reaction mixture and then quickly frozen at 77 K to collect the spectra.

The EPR signal of complex **5** started to decay and completely disappeared within 30 s upon addition of *in-situ* generated **2** (i.e., **1b** + $CoCp^*_2$), indicating conversion into EPR-inactive species (Supplementary Fig. 26). We note that without the addition of **1b** + $CoCp^*_2$, only marginal signal decay of complex **5** was observed even when the sample was warmed up to room temperature for 5 min (Supplementary Fig. 35).

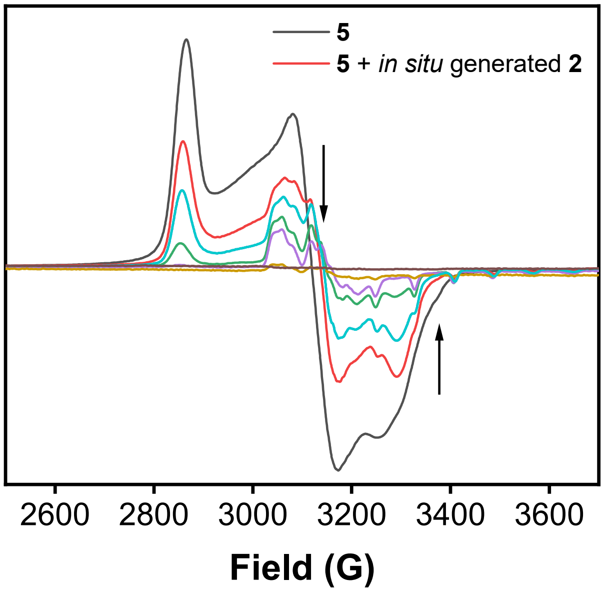

Supplementary Figure 26. EPR signal decay of **5** upon addition of *in situ* generated **2** (i.e., **1b** + $CoCp^*_2$). The initial spectrum of **5** was collected before mixing the sample (black solid line). Each spectrum was recorded at 77 K, after quick thawing to –95 °C for 5 s and subsequent refreezing. The order of the spectra obtained: black-red-blue-green-purple-yellow-dark brown.

NMR spectroscopy: In a N_2-filled glovebox, a 0.3 mL of CD_3OD solution of complex **1b** (^{iPr}N3)$NiCl_2$ (1.2 mg, 1 equiv) and $CoCp^*_2$ (1 mg, 1 equiv) was added into a NMR tube and then quickly frozen at 77 K. Then 0.4 mL of CD_2Cl_2 solution of complex **5** [(^{iPr}N3)Ni^{III}PhAcBr]PF_6 (2 mg, 1 equiv) was added, the solution was quickly mixed, and the ^{1}H NMR was collected at –40°C immediately. After collection of the NMR spectrum, the mixture was warmed up to room temperature for several hours, and then ^{1}H NMR spectrum was collected again.

While the obtained ^{1}H NMR spectrum of the resulting reaction mixture (middle, green spectrum, Supplementary Fig. 27) did not match that of the independently synthesized complex **4** (top, blue spectrum), we observed the formation of organic compounds (Supplementary Fig. 28, bottom: 4,4'-diacetylbiphenyl, 4-bromoacetophenone, 4-methoxyacetophenone) and complex **1b** (^{iPr}N3)$NiCl_2$ (Supplementary Fig. 29; the ^{1}H paramagnetic NMR spectrum of independently synthesized **1b** is shown in Supplementary Fig. 11), which could re-enter the catalytic cycle. While we cannot observe complex **4** directly, two peaks (marked with * in Supplementary Figs. 28 and 30) were observed that are tentatively assigned to a Ni-p-acetylphenyl intermediate that generates acetophenone upon solution workup for GC-MS. We assume that complex **4** is unstable under the

reaction conditions, since an immediate color change from red to yellow was observed for complex **4** in presence of MeCN or MeOH (polar or protic solvent), indicating decomposition or formation of other species. The ^{1}H NMR of complex **4** obtained upon addition of CD_3OD supports this decomposition or formation of a new species (Supplementary Fig. 30).

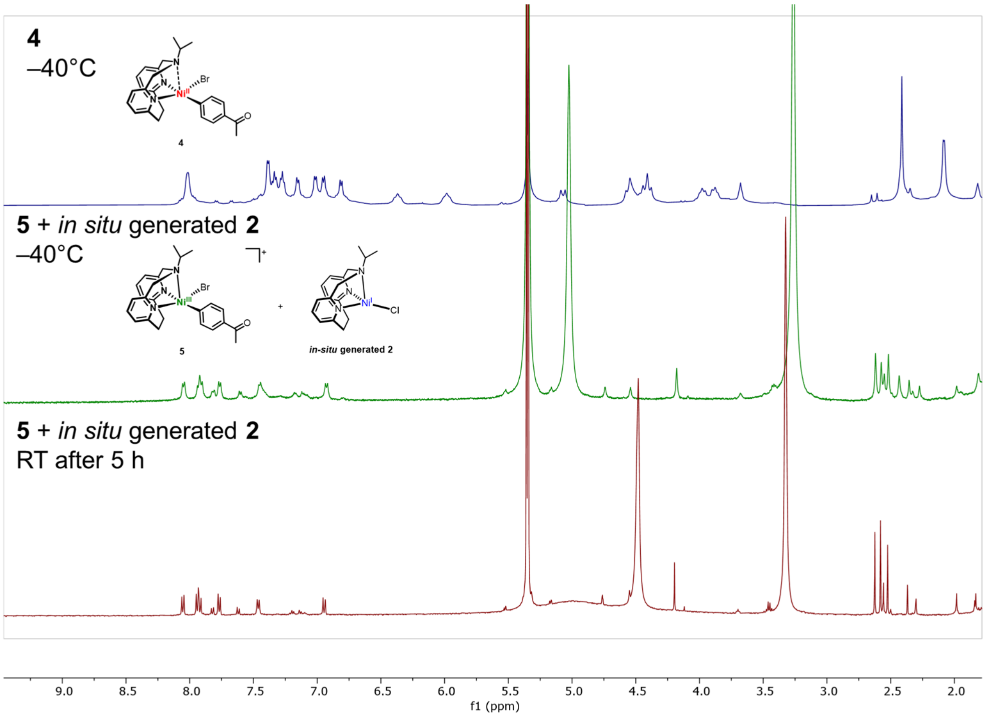

Supplementary Figure 27. ^{1}H NMR spectra of the complex **4** (top, blue), *in situ* generated **2** (i.e., **1b** + $CoCp^*_2$, middle, green), and the reaction mixture after 5 h at RT (bottom, red).

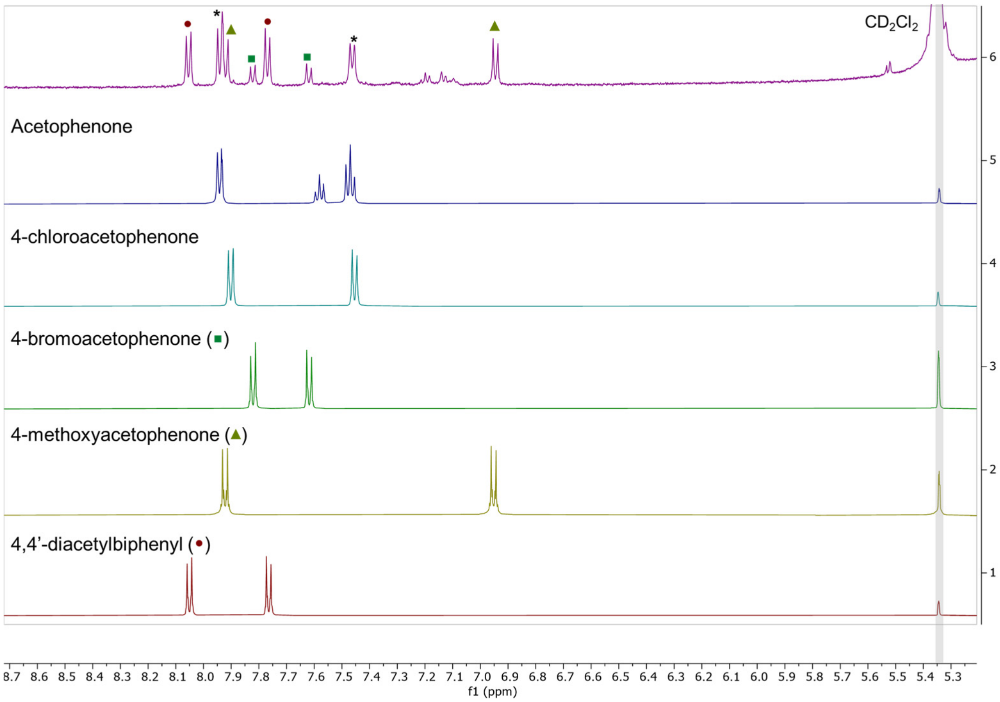

Supplementary Figure 28. Identification of organic products. Enlarged aromatic region of ^{1}H NMR spectrum of the reaction mixture (top, purple) and the assignment of organic products: ● = 4,4'-diacetylbiphenyl, ■ = 4-bromoacetophenone, ▲ = 4-methoxyacetophenone, * = peaks likely belonging to complex **4** (or a derivative of), which generates acetophenone upon workup for GC-MS analysis). All ^{1}H NMR spectra were recorded in $CD_2Cl_2 + CD_3OD$ under N_2.

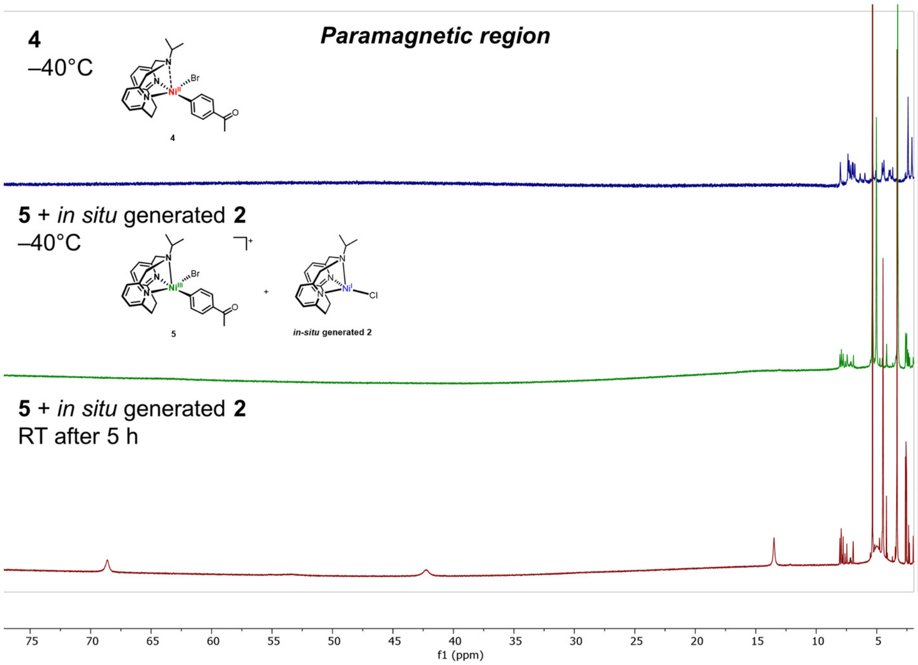

Supplementary Figure 29. ^{1}H NMR paramagnetic spectra of complex **4** (top, blue), *in situ* generated **2** (i.e., **1b** + $CoCp^*_2$, middle, green), and the reaction mixture left at room temperature for 5 h (bottom-red).

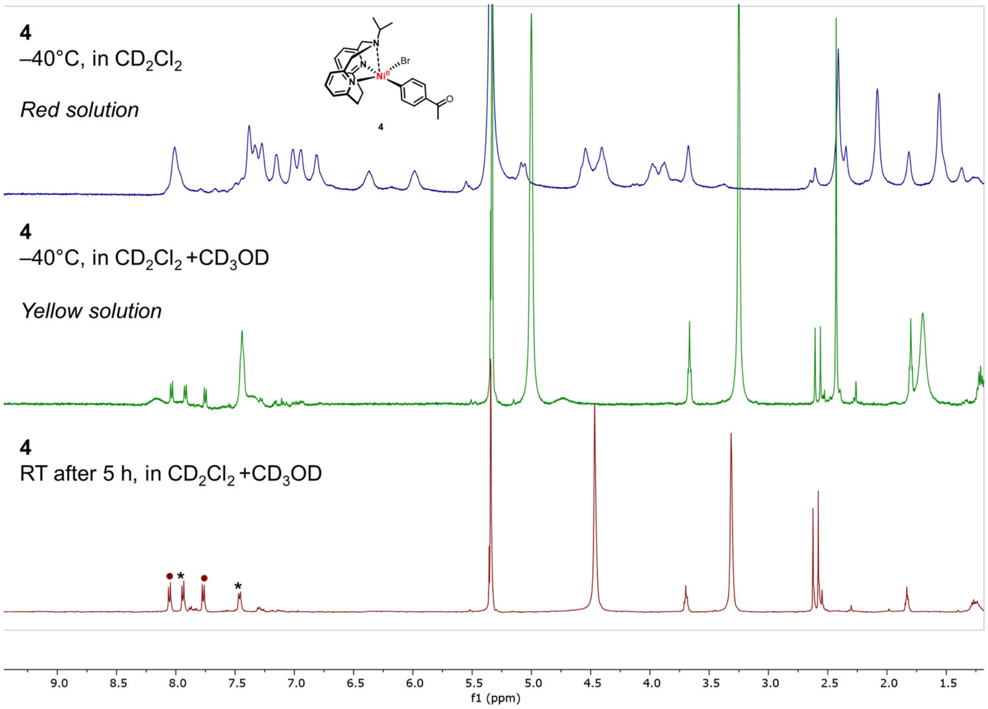

Supplementary Figure 30. ^{1}H NMR spectra of the complex **4** (top, blue) in CD_2Cl_2, the complex **4** in CD_2Cl_2 + CD_3OD (middle, green), and the reaction mixture left at room temperature for 5 h (bottom, red) with assignment of aromatic peaks: ● = 4,4'-diacetylbiphenyl, * = peaks likely belonging to complex **4** (or a derivative of), which generates acetophenone upon workup for GC-MS analysis.

Overall, while we were not able to observe complex **4**, our EPR and NMR results support the deleterious Ni^I/Ni^{III} comproportionation that leads to off-cycle Ni^{II} dihalide species, which later can re-enter the catalytic cycle upon photoexcitation. This Ni^I/Ni^{III} comproportionation reaction is consistent with previously reported computational[4] or experimental studies.[9]

1.11 Electron paramagnetic resonance (EPR) experiments

General experimental conditions: frequency ≈ 9.42 GHz, power = 0.6325 mW, modulation frequency = 100 kHz, modulation amplitude = 5 G, time constant = 82 ms, linewidth = 10–20 G.

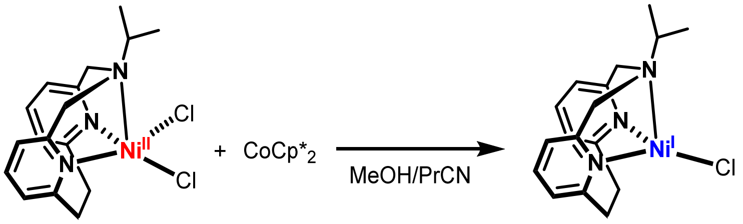

In a N_2-filled glovebox, MeOH solution of (^{iPr}N3)$Ni^{II}Cl_2$ complexes was layered on top of the frozen PrCN solution of chemical reducing agent CoCp*$_2$ in the EPR tube. The mixture was quickly frozen and taken outside of the glovebox. The reaction mixture was quickly mixed in –95 °C cold bath, then frozen to 77 K in the liquid nitrogen for EPR detection.

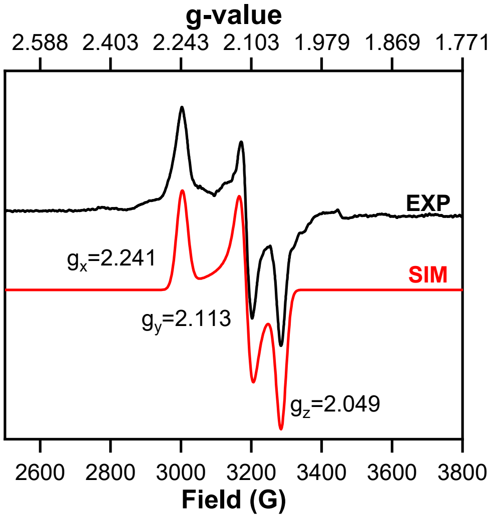

Supplementary Figure 31. EPR spectrum of **1b** + CoCp*$_2$.

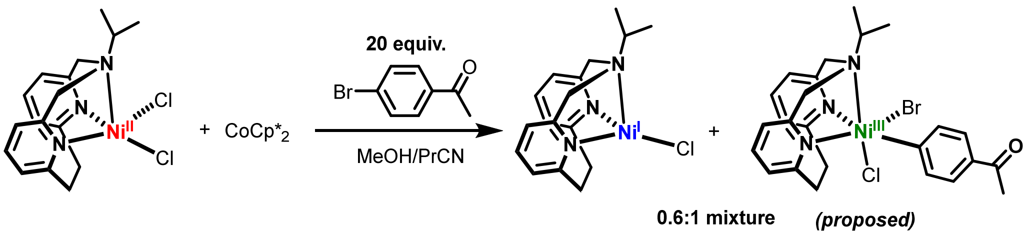

In a N_2-filled glovebox, MeOH solution of (^{iPr}N3)$NiCl_2$ complexes was layered on top of the frozen PrCN solution of chemical reducing agent $CoCp^*_2$ and 4-bromoacetonphenone (20 equiv) in the EPR tube. The mixture was quickly frozen and taken outside of the glovebox. The reaction mixture was quickly mixed in –95 °C cold bath, then frozen to 77 K in the liquid nitrogen for EPR detection. An initial EPR spectrum was taken at 77 K, then the sample was carefully warmed to –80 °C for 10–30 s to allow for the complete reaction to occur and refrozen to 77 K. The EPR spectrum was recorded again. Warming and mixing at this temperature showed degradation of signal from both Ni^I and Ni^{III} species.

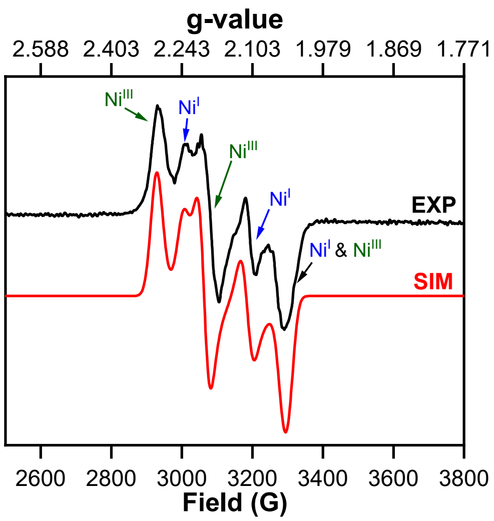

Supplementary Figure 32. EPR spectrum of **1b** + $CoCp^*_2$ + 20 equiv 4-bromoacetophenone.

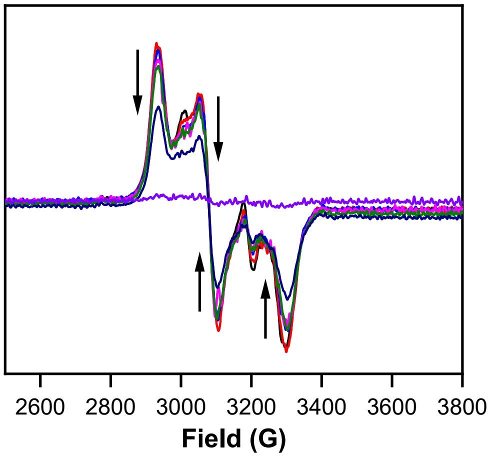

Supplementary Figure 33. EPR signal decays of **1b** + $CoCp^*_2$ + 20 equiv 4-bromoacetophenone over time. Each spectrum was recorded in MeOH/PrCN at 77 K, after quick thawing to –95 °C and subsequent refreezing. The order of the spectra obtained: black-red-blue-pink-green-dark blue-purple.

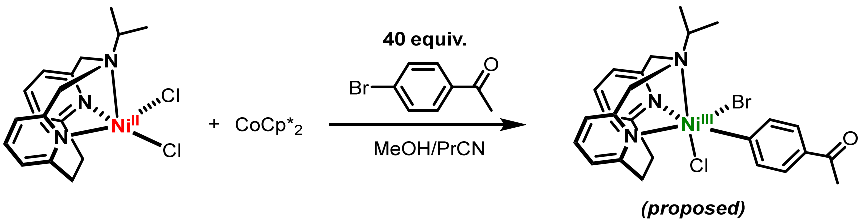

In N_2 filled glovebox, MeOH solution of (^{iPr}N3)$Ni^{II}Cl_2$ complexes was layered on top of the frozen PrCN solution of CoCp*$_2$ and 4-bromoacetonphenone (40 equiv) in the EPR tube. The mixture was quickly frozen and taken outside of the glovebox. The reaction mixture was mixed in –95 °C cold bath, then quickly frozen to 77 K for EPR detection.

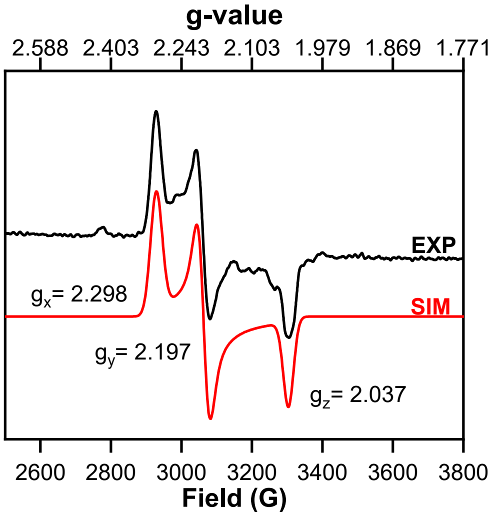

Supplementary Figure 34. EPR spectrum of **1b** + CoCp*$_2$ + 40 equiv 4-bromoacetophenone.

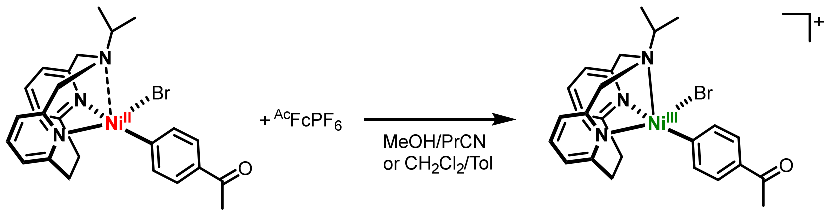

In a N_2-filled glovebox, MeOH:PrCN solution or CH_2Cl_2:Toluene of $^{Ac}FcPF_6$ (1 equiv) was added to the (^{iPr}N3)Ni^{II}(PhAc)Br solid at –40 °C. The reaction mixture was stirred, and the resulting deep red solution was quickly transferred to the EPR tube. After taking out the EPR tube from the glovebox, it was frozen to 77 K for EPR detection.

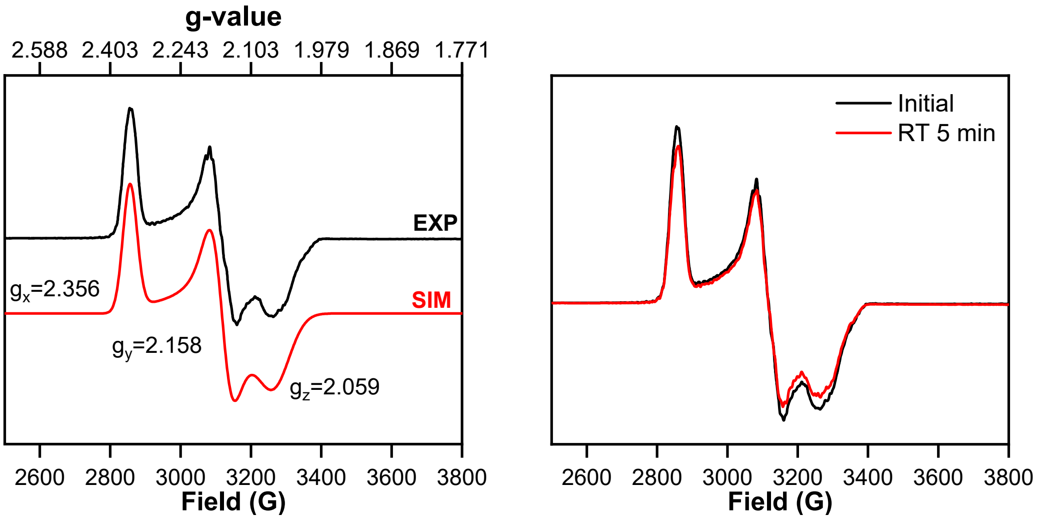

Supplementary Figure 35. EPR spectrum of **5.** EPR spectrum of **5** (left) and signal decay after warming up the sample to RT for 5 min, showing the stability of the species (right).

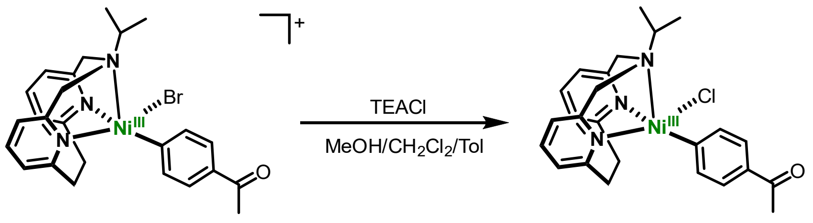

In a N_2-filled glovebox, an EPR tube was charged with 1:3 CH_2Cl_2:Toluene or PrCN solution of $[(^{iPr}N3)Ni^{III}(PhAc)(Br)]^+$ (**5**) and frozen to 77K in liquid nitrogen bath. MeOH solution of tetraethylammonium chloride (TEACl) was added over the frozen solution of **5**. After taking out the EPR tube from the glovebox, and followed by a quick shake of the tube, then the sample was frozen to 77 K for EPR detection to track the decay.

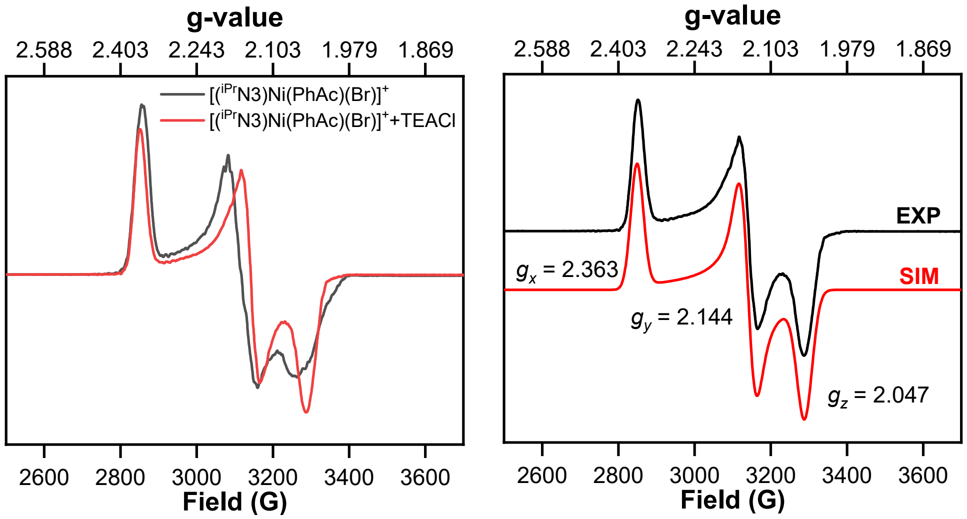

Supplementary Figure 36. EPR spectrum of **6**.

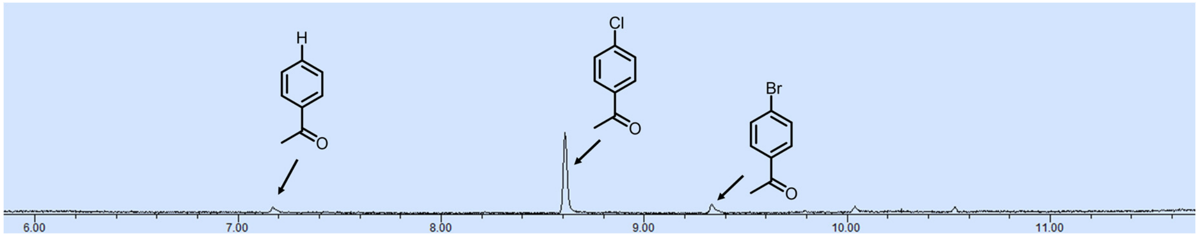

Supplementary Figure 37. Representative GC chromatogram from the EPR reaction mixture (in the absence of MeOH) after warming up to RT, indicating the conversion from $[(^{iPr}N3)Ni^{III}(PhAc)(Br)]^+$ to $[(^{iPr}N3)Ni^{III}(PhAc)(Cl)]^+$. Analysis of the reaction in the presence of MeOH is shown in Supplementary Figs. 49 and 50.

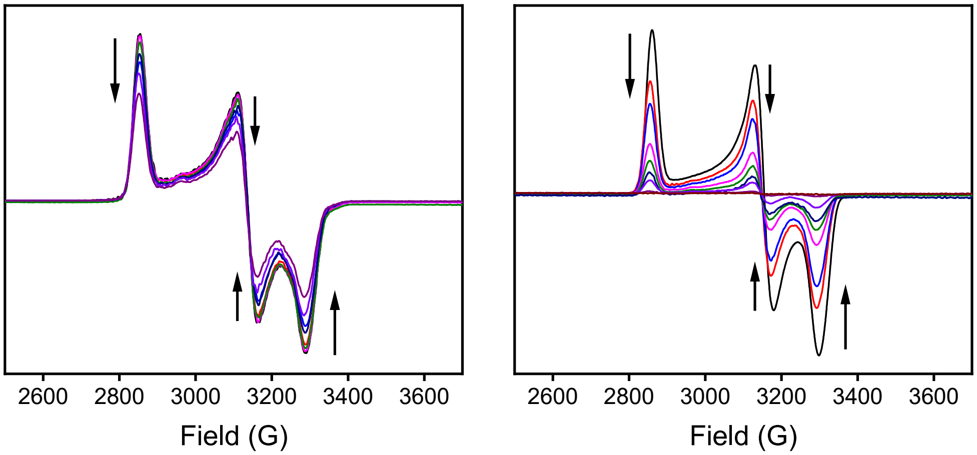

Supplementary Figure 38. EPR spectrum change of **6** generated by 1 equiv. of TEACl (left) and 10 equiv. of TAECl (right) over time, showing faster decay of signal with excess TEACl. Each spectrum was recorded in MeOH/CH_2Cl_2/Toluene at 77 K, after quick thawing to –80 °C and subsequent refreezing. Black solid line represents the initial EPR spectrum of **6**, right after mixing **5** with TEACl. The order of the spectra obtained: black-red-blue-pink-green-dark blue-purple.

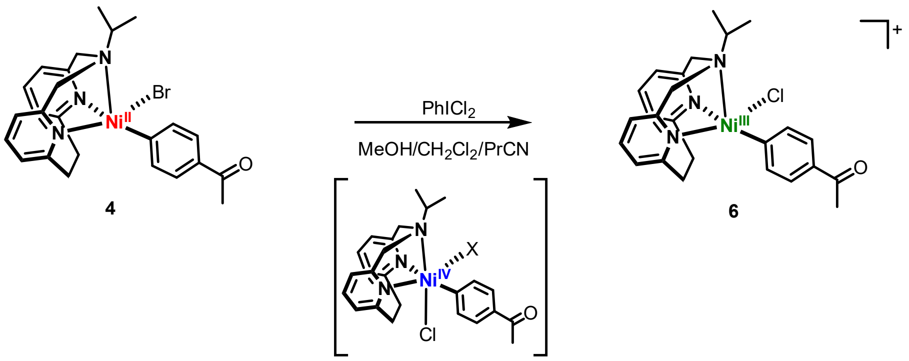

In a N_2-filled glovebox, an EPR tube was charged with CH_2Cl_2 solution of (^{iPr}N3)Ni^{II}(PhAc)Br (**4**) and frozen to 77K in a liquid nitrogen bath. 1:3 MeOH:PrCN solution of $PhICl_2$ (1 equiv) was added over the frozen solution of **4**. After taking out the EPR tube from the glovebox, and followed by a quick shake of the tube, then the sample was frozen to 77 K for an initial EPR spectrum. The Ni^{III} species is thought to be formed by degradation of a transient Ni^{IV} species.

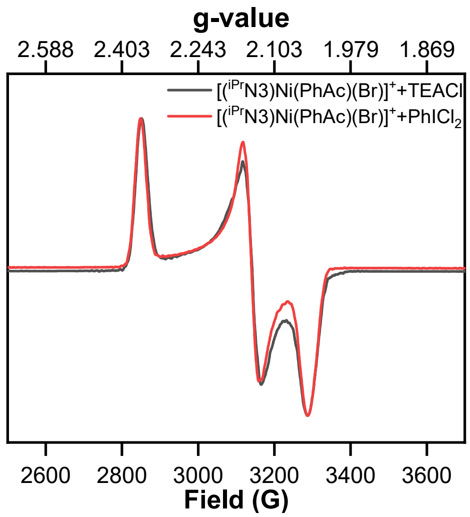

Supplementary Figure 39. EPR spectrum of the reaction overlaid with that of **6**, showing that EPR signal is originating from the identical Ni^{III} species.

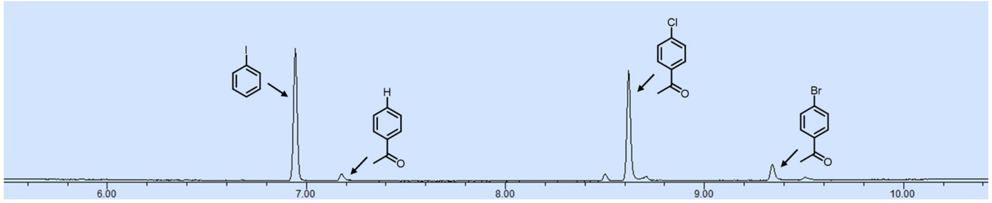

Supplementary Figure 40. Representative GC chromatogram from the EPR reaction mixture after warming up to RT, indicating the conversion from [(^{iPr}N3)Ni^{III}(PhAc)(Br)]$^+$ to [(^{iPr}N3)Ni^{III}(PhAc)(Cl)]$^+$.

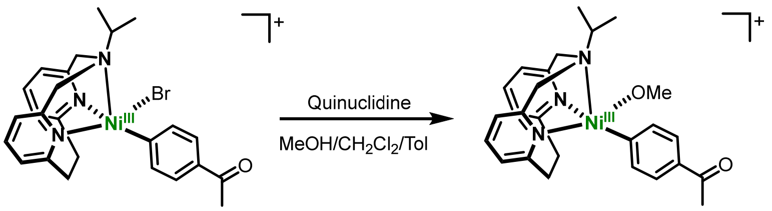

In a N_2-filled glovebox, an EPR tube was charged with 1:3 CH_2Cl_2:Toluene solution of [(^{iPr}N3)Ni^{III}(PhAc)(Br)]$^+$ (**5**) and frozen to 77 K in liquid nitrogen bath. MeOH solution of quinuclidine was added over the frozen solution of **5**. After taking out the EPR tube from the glovebox, and followed by a quick shake of the tube, then the sample was frozen to 77 K for initial EPR detection. The sample was then carefully warmed for 10 s and refrozen, the EPR spectrum was recorded again to track the decay.

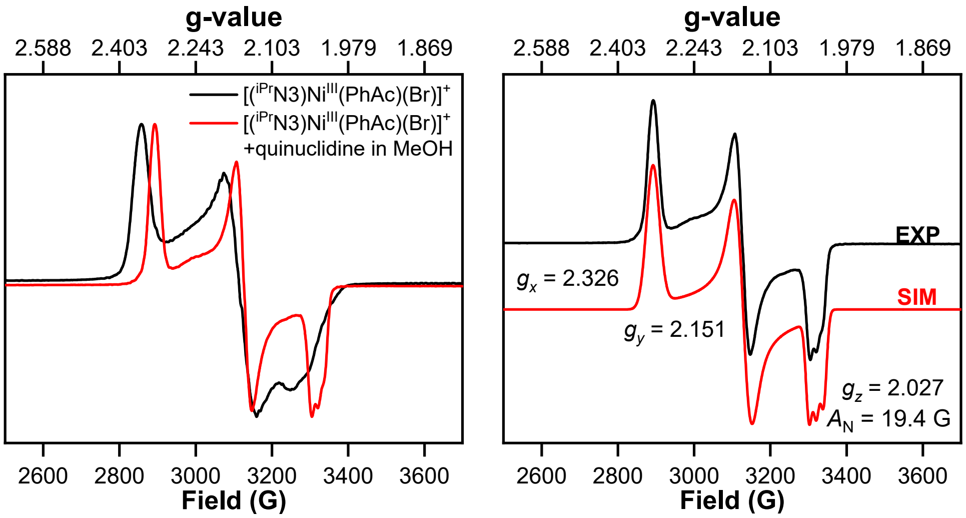

Supplementary Figure 41. EPR spectrum of **7**.

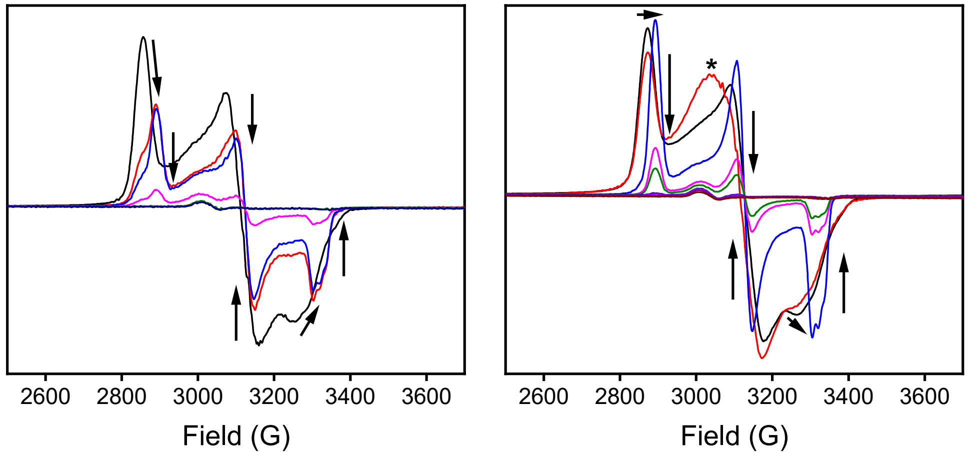

Supplementary Figure 42. EPR spectrum change of **7** generated by 1 equiv. of quinuclidine (left) and 10 equiv. of quinuclidine (right) over time. Each spectrum was recorded in MeOH/CH_2Cl_2/Toluene at 77K, after quick thawing to –80 °C and subsequent refreezing. Black solid line represents the initial EPR spectrum of **5**, before mixing with quinuclidine. Red solid line shows signals from a mixture of **5** and **7**, and the signal marked with ***** proposed to be originated from quinuclidine bound species as it appears prominent with a higher concentration of quinuclidine. (The order of the spectrum obtained: black-red-blue-pink-green-dark blue-purple)

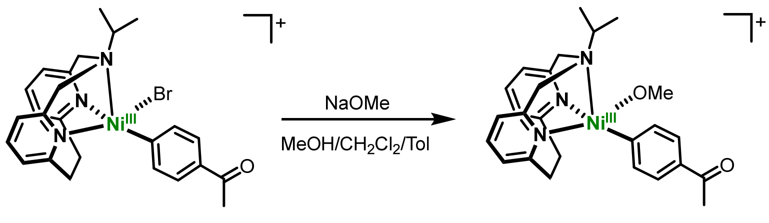

In a N_2-filled glovebox, an EPR tube was charged with 1:3 CH_2Cl_2:Toluene solution of $[(^{iPr}N3)Ni^{III}(PhAc)(Br)]^+$ (**5**) and frozen to 77 K in a liquid nitrogen bath. A MeOH solution of NaOMe was added over the frozen solution of **5**. After taking out the EPR tube from the glovebox, and followed by a quick shake of the tube, the sample was frozen to 77 K for an initial EPR spectrum. The sample was then carefully warmed for 10 s and refrozen, and the EPR spectrum was recorded again to track the decay.

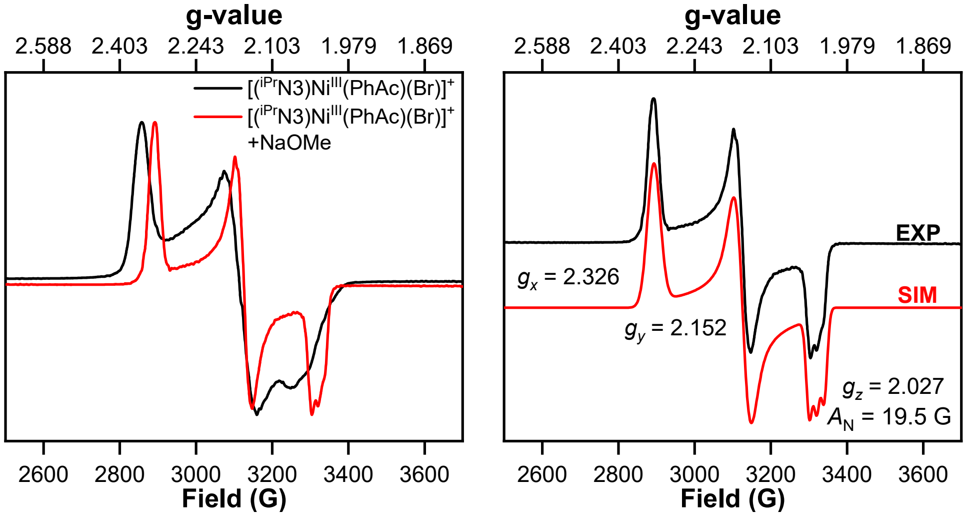

Supplementary Figure 43. EPR spectrum of 7.

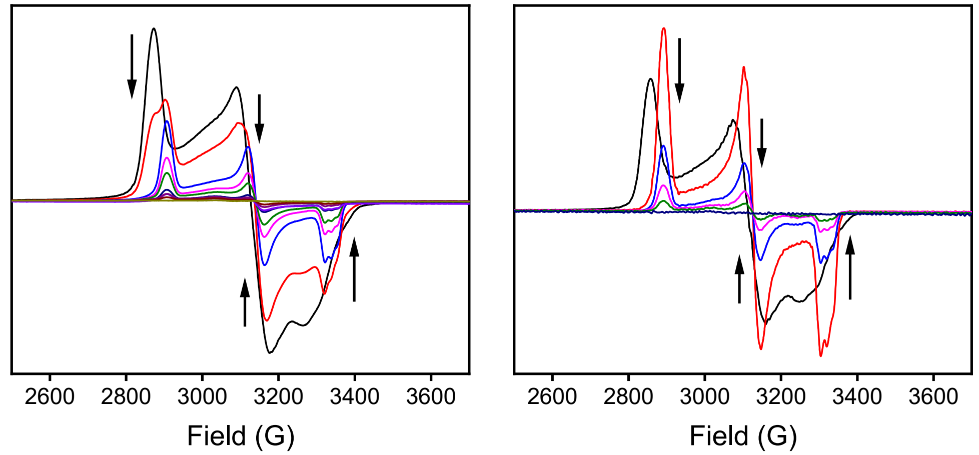

Supplementary Figure 44. EPR spectral change of **7** generated by 1 equiv. of NaOMe (left) and 10 equiv. of NaOMe (right) over time. Each spectrum was recorded in MeOH/CH_2Cl_2/Toluene at 77 K, after quick thawing to –80 °C and subsequent refreezing. Black solid line represents the initial EPR spectrum of **5**, before mixing with NaOMe. Red solid line in the left graph shows signals from a mixture of **5** and **7**, while in the right graph shows signals only from **7**. The order of the spectra obtained: black-red-blue-pink-green-dark blue-purple.

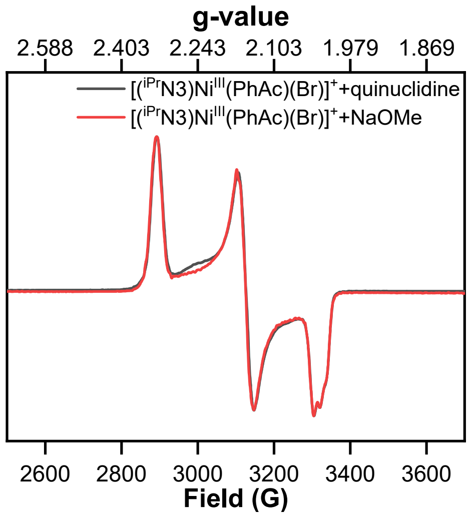

Supplementary Figure 45. Overlaid EPR spectra of **7** obtained by addition of quinuclidine (black solid line) or NaOMe (red solid line), confirming the generation of an identical species.

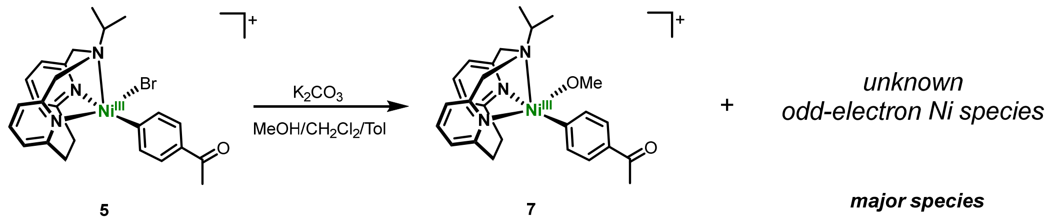

5

7

In a N_2-filled glovebox, an EPR tube was charged with a 1:3 CH_2Cl_2:Toluene solution of $[(^{iPr}N3)Ni^{III}(PhAc)(Br)]^+$ (**5**) and frozen to 77 K in a liquid nitrogen bath. A MeOH solution of K_2CO_3 was added over the frozen solution of **5**. After taking out the EPR tube from the glovebox, and followed by a quick shake of the tube, the sample was frozen to 77 K for an initial EPR spectrum. The sample was then carefully warmed for 10 s and refrozen, and the EPR spectrum was recorded again to track the decay.

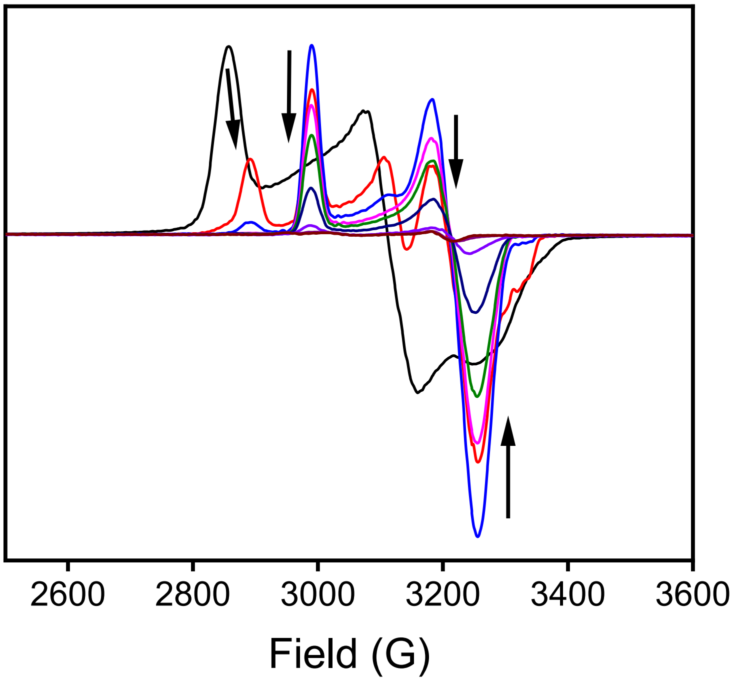

Supplementary Figure 46. EPR spectral changes of **5** upon the addition of K_2CO_3 in the presence of MeOH. Each spectrum was recorded in MeOH/CH_2Cl_2/Toluene at 77 K, after a quick thawing to –80 °C and subsequent refreezing. Black solid line represents the initial EPR spectrum of **5**, before mixing with K_2CO_3. The red and blue solid lines show signals from a mixture of **7** and an unknown Ni species, while the pink solid line shows signal only originating from unknown Ni species. Separated red and pink solid lines are shown in the Figure S34. The order of the spectra obtained: black-red-blue-pink-green-dark blue-purple.

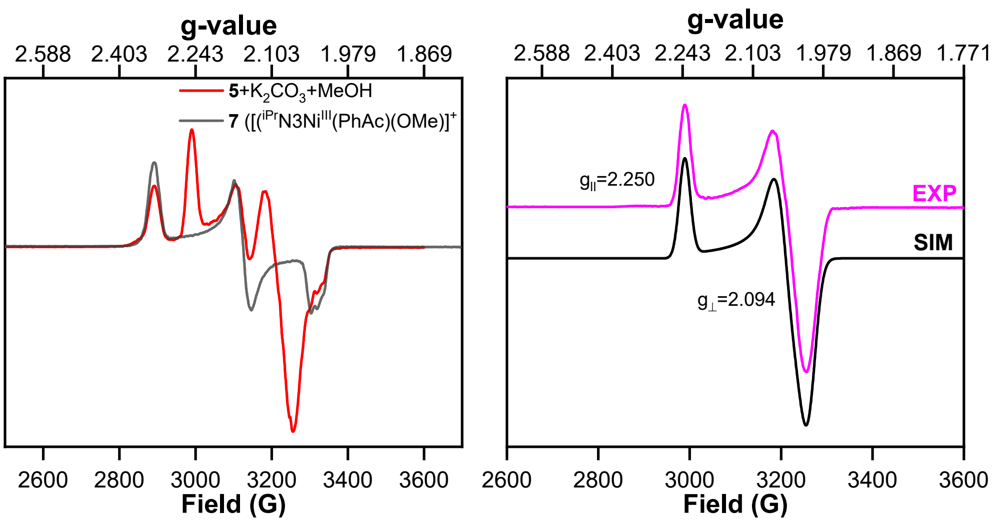

Supplementary Figure 47. The EPR spectrum corresponding to the red solid line in Supplementary Figure 46. Evidence of a mixture of **7** and an unknown Ni species (left) and the EPR spectrum corresponding to the pink solid line in Supplementary Figure 46, indicating the presence of a single axial EPR signal (right).

EPR glassing solvent effect for 6

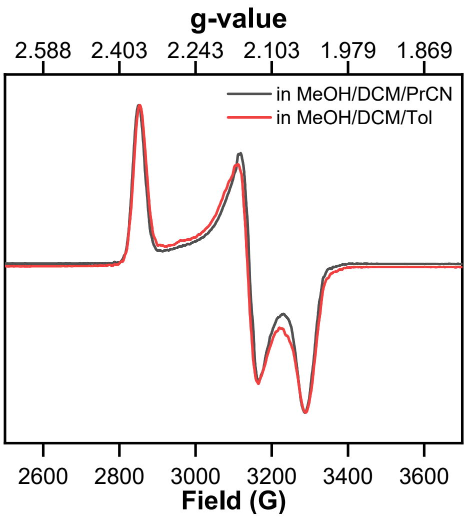

Supplementary Figure 48. EPR spectra of **6** in different glassing solvents (PrCN or Toluene), showing the minimal effect of glassing solvent.

Supplementary Table 10. Parameters for the EPR spectra of the various Ni species, including the DFT (B3LYP/tzvp) calculated EPR parameters for **5**.

**Species**	g_x	g_y	g_z
**(**^{iPr}N3)Ni^{I}Cl (**2**)	2.241	2.113	2.049
(^{iPr}N3)Ni^{III}(PhAc)BrCl (**3**)	2.298	2.197	2.037
[(^{iPr}N3)Ni^{III}(PhAc)(Br)]$^{+}$ (**5**)	2.356	2.158	2.059
DFT (B3LYP/tzvp) Calculated for ***5***	*2.338*	*2.179*	*2.068*
[(^{iPr}N3)Ni^{III}(PhAc)(Cl)]$^{+}$ (**6**)	2.363	2.144	2.047
[(^{iPr}N3)Ni^{III}(PhAc)(OMe)]$^{+}$ (**7**)	2.326	2.152	2.027

1.12 Reactivity study of ^{iPr}N3Ni^{III} species

General procedure for the reactivity studies of (^{iPr}N3)Ni^{III} complexes: In N_2-flled glove box, 3 mg of [(^{iPr}N3)Ni^{III}(PhAc)(Br)]$^+$ (**5**) in 1 mL of CH_2Cl_2 was added into a NMR tube at –40 °C and then frozen. Cooled (–40 °C) 0.5 mL MeOH solution of TEACl, quinuclidine or NaOMe was layered on top of the frozen CH_2Cl_2 solution. After taking out the NMR tube from the glove box, and followed by a quick shake of the tube, the sample was warmed to room temperature. After 5 min, 1,3-benzodioxole as a standard was added to the reaction mixture and extracted with ethyl ether. The residue was analyzed by GC-FID to give the yield of the reaction.

Addition of TEACl, quinuclidine with MeOH or NaOMe generated **6** or **7**, which underwent C–O bond-forming reductive elimination to afford 4-methoxyacetophenone with acetophenone side product (no Ar–Ar homo-coupled product was observed).

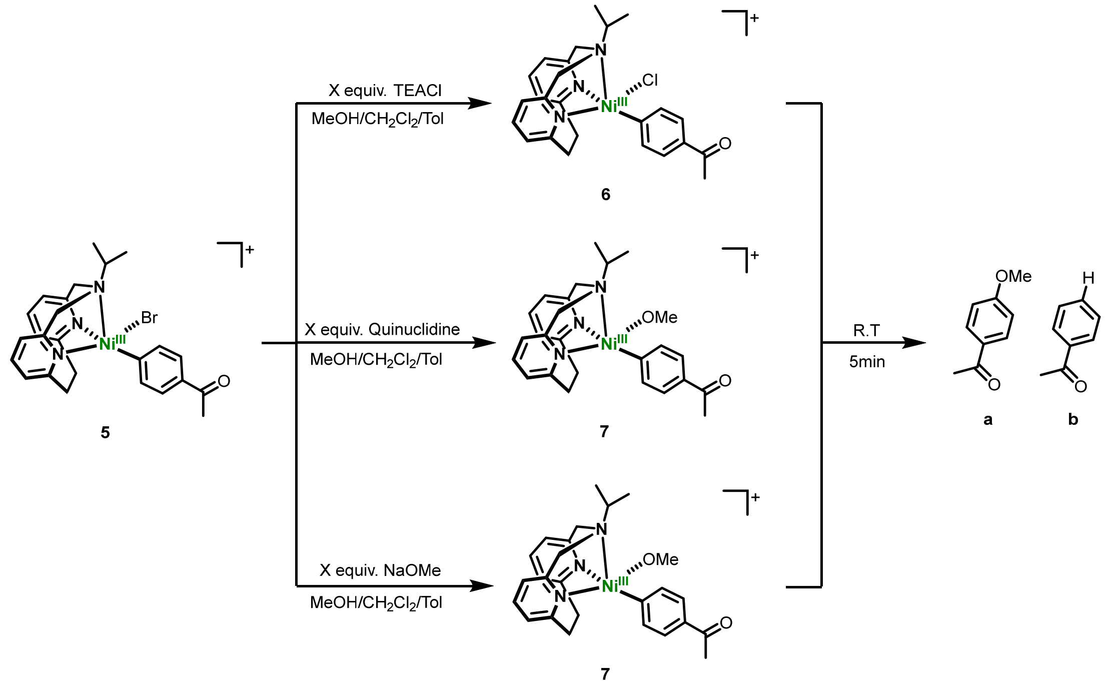

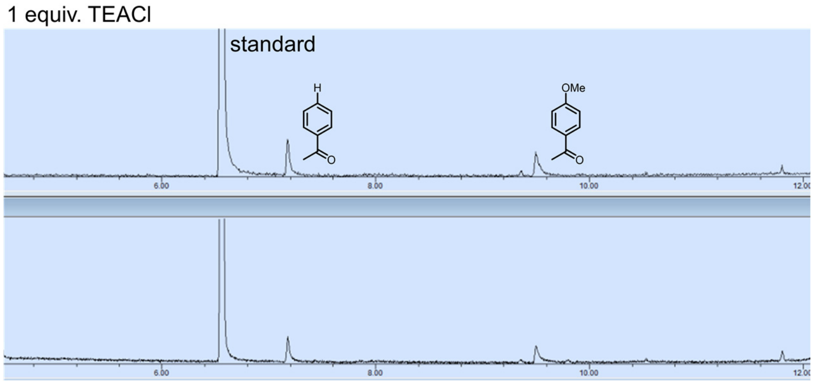

Supplementary Figure 49. Representative GC chromatogram from the reaction of **5** with 1 equiv of TEACl.

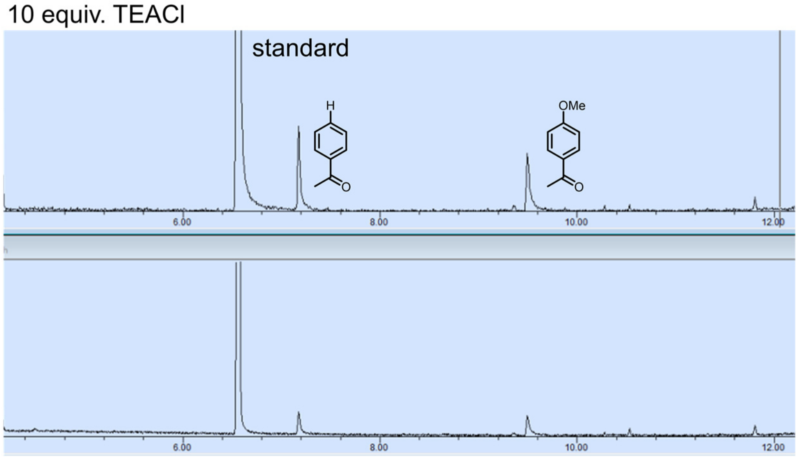

Supplementary Figure 50. Representative GC chromatogram from the reaction of **5** with 10 equiv of TEACl.

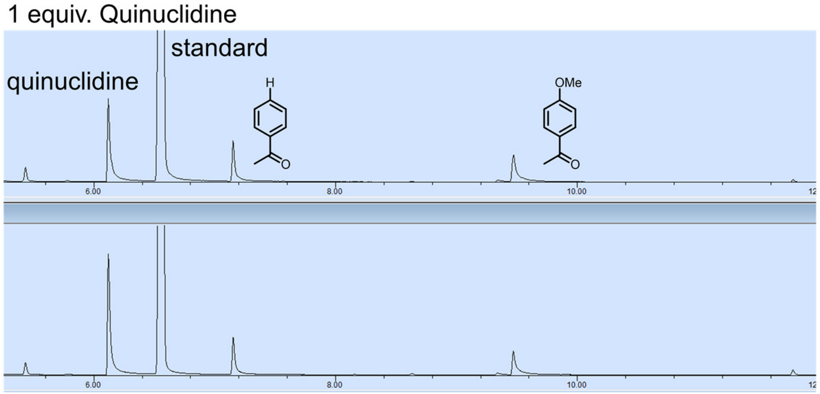

Supplementary Figure 51. Representative GC chromatogram from the reaction of **5** with 1 equiv of quinuclidine.

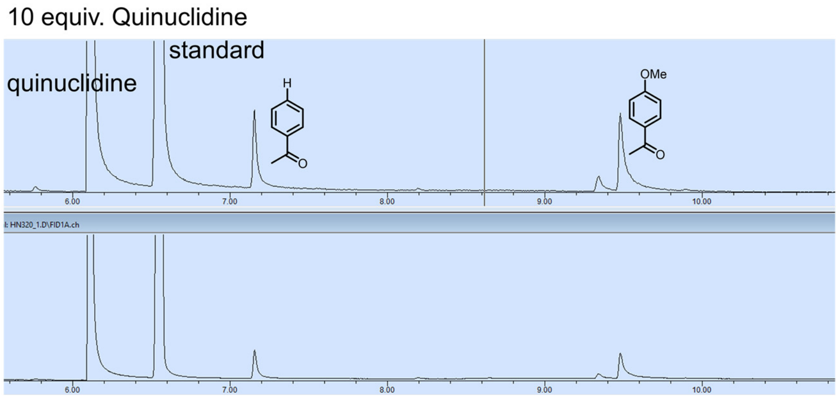

Supplementary Figure 52. Representative GC chromatogram from the reaction of **5** with 10 equiv of quinuclidine.

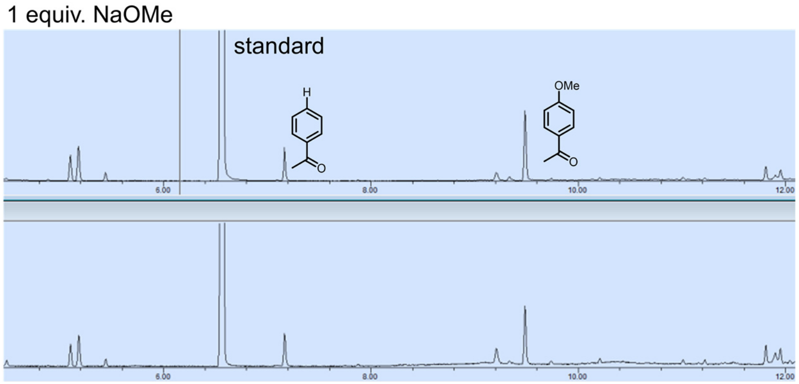

Supplementary Figure 53. Representative GC chromatogram from the reaction of **5** with 1 equiv of NaOMe.

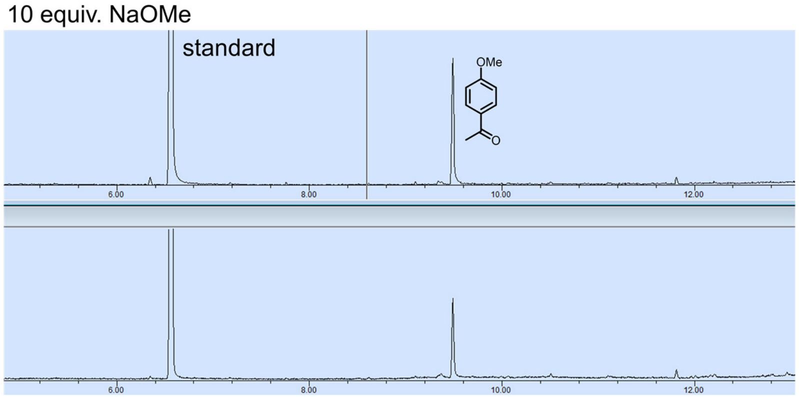

Supplementary Figure 54. Representative GC chromatogram from the reaction of **5** with 10 equiv of NaOMe.

1.13 X-ray structure Characterization

General information.

Single crystals were grown by vapor diffusion of pentane into concentrated solution of CH_2Cl_2 and MeOH. Crystals were mounted on a Bruker D8 Venture kappa diffractometer equipped with a Photon II CPAD detector. An Iμs microfocus Mo source (λ = 0.71073 Å) coupled with a multi-layer mirror monochromator provided the incident beam. The sample was mounted on a nylon loop with the minimal amount of Paratone-N oil. Data was collected as a series of φ and/or ω scans. Data were collected at 100 K using a cold stream of N_2(g). The collection, cell refinement, and integration of intensity data was carried out with the APEXIII software.[15] A multiscan absorption correction was performed with SADABS or TWINABS-2012/1.[16] The structure was phased with intrinsic methods using SHELXT and refined with the full-matrix least-squares program SHELXL.[17] Hydrogen atoms were placed in calculated positions using the standard riding model and refined isotropically; all non-hydrogen atoms were refined anisotropically. The crystal of $^{iPr}N3Ni^{II}Cl_2$ (**1b**) was refined as a twin (Scales: 0.811(2) 0.189(2)). The structure of $^{iPr}N3Ni^{II}Cl_2$ (**1b**) included electron density attributed to heavily disordered solvents that could not be modeled, necessitating the use of the SQUEEZE function in PLATON.[18]

Computer programs: *SAINT* V8.38A (2016), *SHELXT* (Sheldrick, 2015), XL (Sheldrick, 2008), Olex2 (Dolomanov *et al.*, 2009).

The deposition numbers CCDC 2085516 (**1a**) and 2085517 (**1b**) at the Cambridge Crystallographic Data Centre CCDC contain the supplementary crystallographic data. These data are provided free of charge by the Cambridge Crystallographic Data Centre. Crystallographic details are summarized in Supplementary Tables 11−14.

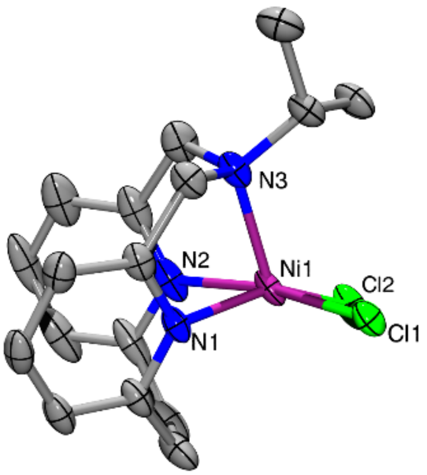

Supplementary Figure 55. ORTEP representation of $^{iPr}N3Ni^{II}Cl_2$ with 50% probability thermal ellipsoids.

X-ray structure determination of (^{Me}N3)Ni^{II}Cl$_2$ (1a)

Supplementary Table 11. Crystal data and structure refinement for (^{Me}N3)Ni^{II}Cl$_2$ (**1a**)

Crystal data	
Chemical formula	$C_{15}H_{17}Cl_2N_3Ni$
M_r	368.92
Crystal system, space group	Orthorhombic, *Pnma*
Temperature (K)	100
a, *b*, *c* (Å)	15.2470 (4), 13.2816 (3), 7.5023 (2)
V (Å^3)	1519.25 (7)
Z	4
Radiation type	Mo *K*a
m (mm^{-1})	1.62
Crystal size (mm)	0.14 × 0.10 × 0.06
Data collection	
Diffractometer	Bruker Kappa/PhotonII CCD
Absorption correction	Multi-scan<br>*SADABS2016*/2 (Bruker,2016/2) was used for absorption correction. wR2(int) was 0.0741 before and 0.0529 after correction. The Ratio of minimum to maximum transmission is 0.9284. The l/2 correction factor is Not present.

T_{min}, T_{max}	0.692, 0.746
No. of measured, independent and observed [$I > 2s(I)$] reflections	17175, 1968, 1811
R_{int}	0.040
(sin q/l)$_{max}$ (Å^{-1})	0.667
Refinement	
$R[F^2 > 2s(F^2)]$, $wR(F^2)$, S	0.022, 0.055, 1.09
No. of reflections	1968
No. of parameters	107
H-atom treatment	H atoms treated by a mixture of independent and constrained refinement
Dρ$_{max}$, Dρ$_{min}$ (e Å^{-3})	0.37, -0.32

Supplementary Table 12. Bond length (Å) and angles (°) for $^{Me}N3Ni^{II}Cl_2$ (**1a**)

Ni1—Cl1^i	2.3219 (4)	C13—C14	1.5019 (19)
Ni1—Cl1	2.3220 (4)	C8—H8A	0.9900
Ni1—N1^i	2.0580 (11)	C8—H8B	0.9900
Ni1—N1	2.0579 (11)	C10—H10	0.9500
Ni1—N2	2.0559 (16)	C10—C11	1.391 (2)
N1—C9	1.3410 (17)	C12—H12	0.9500
N1—C13	1.3501 (17)	C12—C11	1.388 (2)
N2—C8^i	1.4949 (16)	C14—C14^i	1.574 (3)
N2—C8	1.4948 (16)	C14—H14A	0.9900
N2—C15	1.478 (2)	C14—H14B	0.9900
C9—C8	1.5089 (19)	C15—H15A	0.948 (18)
C9—C10	1.3868 (19)	C15—H15B	0.98 (3)
C13—C12	1.3893 (19)	C11—H11	0.9500

Symmetry code(s): (i) x, $-y+1/2$, z.

Cl1^i—Ni1—Cl1	96.55 (2)	N2—C8—C9	111.59 (12)
N1—Ni1—Cl1^i	92.89 (3)	N2—C8—H8A	109.3
N1^i—Ni1—Cl1^i	165.10 (3)	N2—C8—H8B	109.3
N1—Ni1—Cl1	165.10 (3)	C9—C8—H8A	109.3
N1^i—Ni1—Cl1	92.88 (3)	C9—C8—H8B	109.3

N1—Ni1—N1^{i}	75.72 (6)	H8A—C8—H8B	108.0
N2—Ni1—Cl1	105.37 (3)	C9—C10—H10	120.9
N2—Ni1—Cl1^{i}	105.37 (3)	C9—C10—C11	118.30 (13)
N2—Ni1—N1	83.07 (5)	C11—C10—H10	120.9
N2—Ni1—N1^{i}	83.07 (5)	C13—C12—H12	120.5
C9—N1—Ni1	113.39 (9)	C11—C12—C13	119.00 (13)
C9—N1—C13	120.19 (11)	C11—C12—H12	120.5
C13—N1—Ni1	123.56 (9)	C13—C14—C14^{i}	114.86 (7)
C8^{i}—N2—Ni1	106.49 (9)	C13—C14—H14A	108.6
C8—N2—Ni1	106.49 (9)	C13—C14—H14B	108.6
C8—N2—C8^{i}	112.91 (15)	C14^{i}—C14—H14A	108.6
C15—N2—Ni1	113.67 (13)	C14^{i}—C14—H14B	108.6
C15—N2—C8	108.68 (10)	H14A—C14—H14B	107.5
C15—N2—C8^{i}	108.68 (10)	N2—C15—H15A	109.7 (10)
N1—C9—C8	114.99 (12)	N2—C15—H15B	111.8 (14)
N1—C9—C10	121.72 (13)	H15A—C15—H15B	109.4 (13)
C10—C9—C8	123.28 (12)	C10—C11—H11	120.1
N1—C13—C12	120.71 (12)	C12—C11—C10	119.72 (13)
N1—C13—C14	116.21 (12)	C12—C11—H11	120.1
C12—C13—C14	122.96 (12)		

Symmetry code(s): (i) *x*, −*y*+1/2, *z*.

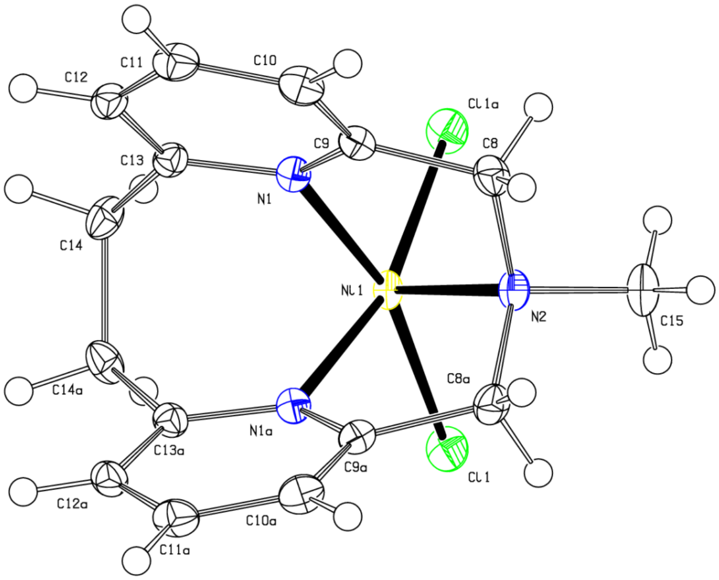

Supplementary Figure 56. Projection view of (^{Me}N3)Ni^{II}Cl$_2$ (**1a**) with 50% probability ellipsoids.

X-ray structure determination of (^{iPr}N3)Ni^{II}Cl$_2$ (1b)

Supplementary Table 13. Crystal data and structure refinement for (^{iPr}N3)Ni^{II}Cl$_2$ (**1b**)

Crystal data	
Chemical formula	$C_{17}H_{21}Cl_2N_3Ni$
M_r	396.98
Crystal system, space group	Orthorhombic, *Pbca*
Temperature (K)	100
a, *b*, *c* (Å)	11.0592 (4), 14.6160 (5), 24.2709 (9)
V (Å^3)	3923.2 (2)
Z	8
Radiation type	Mo *K*α
μ (mm^{-1})	1.26
Crystal size (mm)	0.25 × 0.18 × 0.13
Data collection	
Diffractometer	Bruker Kappa/PhotonII CCD
Absorption correction	TWINABS-2012/1 (Bruker,2012) was used for absorption correction.
No. of measured, independent and observed [$I > 2\sigma(I)$] reflections	189376, 189376, 149148
R_{int}	0.035
$(\sin \theta/\lambda)_{max}$ (Å^{-1})	0.651
Refinement	
$R[F^2 > 2\sigma(F^2)]$, $wR(F^2)$, S	0.041, 0.099, 1.04
No. of reflections	189376
No. of parameters	211
H-atom treatment	H-atom parameters constrained
$\Delta\rangle_{max}$, $\Delta\rangle_{min}$ (e Å^{-3})	0.52, -0.65

Supplementary Table 14. Bond length (Å) and angles (°) for $^{iPr}N3Ni^{II}Cl_2$ (**1b**)

Ni1—Cl1	2.3406(7)	C14—H14B	0.9900
Ni1—Cl2	2.3049(8)	C14—C13	1.515(4)
Ni1—N1	2.024(2)	C15—H15	1.0000
Ni1—N3	2.087(2)	C15—C17	1.522(4)
Ni1—N2	2.061(2)	C15—C16	1.518(4)
N1—C2	1.334(3)	C13—C12	1.392(4)
N1—C6	1.354(3)	C9—C10	1.378(5)
N3—C1	1.498(3)	C9—C8	1.489(5)
N3—C14	1.495(3)	C7—H7A	0.9900
N3—C15	1.517(3)	C7—H7B	0.9900
N2—C13	1.327(4)	C7—C8	1.559(4)
N2—C9	1.363(4)	C17—H17A	0.9800
C2—C1	1.506(4)	C17—H17B	0.9800
C2—C3	1.383(4)	C17—H17C	0.9800
C1—H1A	0.9900	C16—H16A	0.9800
C1—H1B	0.9900	C16—H16B	0.9800
C4—H4	0.9500	C16—H16C	0.9800
C4—C3	1.388(4)	C12—H12	0.9500
C4—C5	1.379(4)	C12—C11	1.394(5)
C3—H3	0.9500	C11—H11	0.9500
C6—C5	1.385(4)	C11—C10	1.367(5)
C6—C7	1.494(4)	C10—H10	0.9500
C5—H5	0.9500	C8—H8A	0.9900
C14—H14A	0.9900	C8—H8B	0.9900

Cl2—Ni1—Cl1	97.10(3)	C13—C14—H14A	109.2
N1—Ni1—Cl1	89.92(6)	C13—C14—H14B	109.2
N1—Ni1—Cl2	151.93(7)	N3—C15—H15	106.7
N1—Ni1—N3	83.86(9)	N3—C15—C17	113.2(2)
N1—Ni1—N2	76.22(8)	N3—C15—C16	112.1(2)
N3—Ni1—Cl1	99.70(6)	C17—C15—H15	106.7

N3—Ni1—Cl2	121.26(6)	C16—C15—H15	106.7
N2—Ni1—Cl1	165.55(6)	C16—C15—C17	110.9(2)
N2—Ni1—Cl2	93.44(7)	N2—C13—C14	116.4(3)
N2—Ni1—N3	83.08(10)	N2—C13—C12	121.8(3)
C2—N1—Ni1	113.81(17)	C12—C13—C14	121.8(3)
C2—N1—C6	121.4(2)	N2—C9—C10	119.8(4)
C6—N1—Ni1	121.99(18)	N2—C9—C8	117.5(3)
C1—N3—Ni1	103.98(16)	C10—C9—C8	122.5(3)
C1—N3—C15	108.22(19)	C6—C7—H7A	108.6
C14—N3—Ni1	106.75(17)	C6—C7—H7B	108.6
C14—N3—C1	111.4(2)	C6—C7—C8	114.5(3)
C14—N3—C15	111.7(2)	H7A—C7—H7B	107.6
C15—N3—Ni1	114.51(17)	C8—C7—H7A	108.6
C13—N2—Ni1	113.56(18)	C8—C7—H7B	108.6
C13—N2—C9	120.6(3)	C15—C17—H17A	109.5
C9—N2—Ni1	122.5(2)	C15—C17—H17B	109.5
N1—C2—C1	114.7(2)	C15—C17—H17C	109.5
N1—C2—C3	120.9(2)	H17A—C17—H17B	109.5
C3—C2—C1	124.5(3)	H17A—C17—H17C	109.5
N3—C1—C2	112.3(2)	H17B—C17—H17C	109.5
N3—C1—H1A	109.1	C15—C16—H16A	109.5
N3—C1—H1B	109.1	C15—C16—H16B	109.5
C2—C1—H1A	109.1	C15—C16—H16C	109.5
C2—C1—H1B	109.1	H16A—C16—H16B	109.5
H1A—C1—H1B	107.9	H16A—C16—H16C	109.5
C3—C4—H4	120	H16B—C16—H16C	109.5
C5—C4—H4	120	C13—C12—H12	121.3
C5—C4—C3	119.9(3)	C13—C12—C11	117.4(4)
C2—C3—C4	118.6(3)	C11—C12—H12	121.3
C2—C3—H3	120.7	C12—C11—H11	119.9
C4—C3—H3	120.7	C10—C11—C12	120.3(3)
N1—C6—C5	119.7(3)	C10—C11—H11	119.9
N1—C6—C7	114.9(2)	C9—C10—H10	120.1
C5—C6—C7	125.1(3)	C11—C10—C9	119.8(3)
C4—C5—C6	119.4(3)	C11—C10—H10	120.1

C4—C5—H5	120.3	C9—C8—C7	115.3(3)
C6—C5—H5	120.3	C9—C8—H8A	108.5
N3—C14—H14A	109.2	C9—C8—H8B	108.5
N3—C14—H14B	109.2	C7—C8—H8A	108.5
N3—C14—C13	111.9(2)	C7—C8—H8B	108.5
H14A—C14—H1	4B 107.9	H8A—C8—H8B	107.5

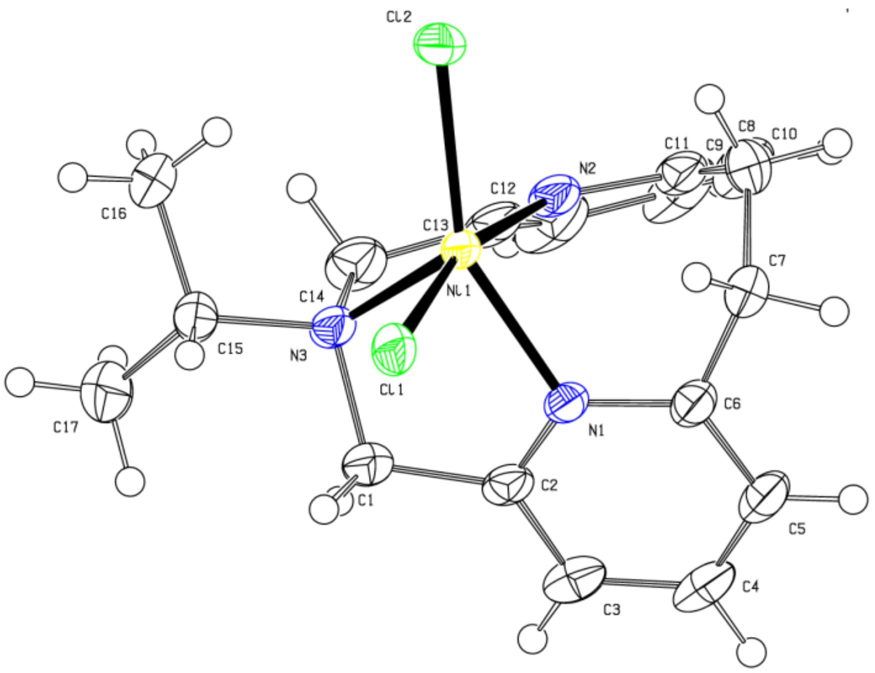

Supplementary Figure 57. Projection view of (^{iPr}N3)Ni^{II}Cl$_2$ (**1b**) with 50% probability ellipsoids.

1.14 Space filling model of 1a and 1b

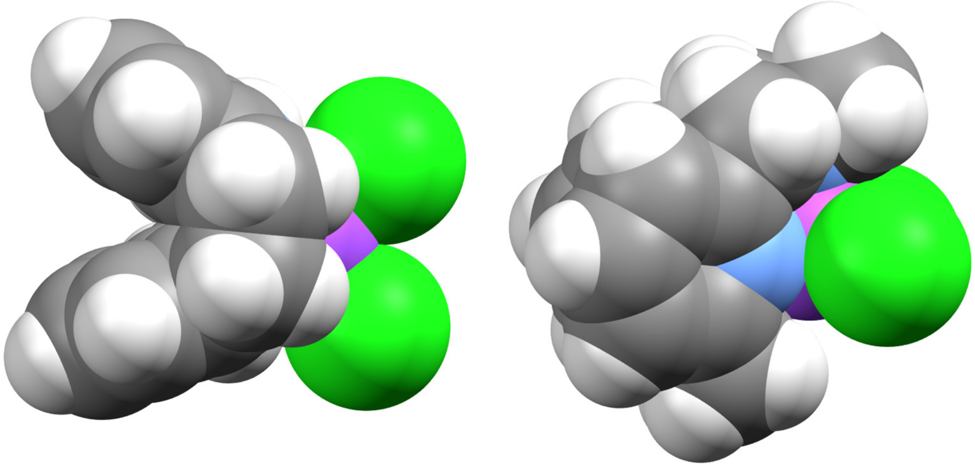

Supplementary Figure 58. The space filling model of $(^{Me}N3)Ni^{II}Cl_2$ (**1a**)

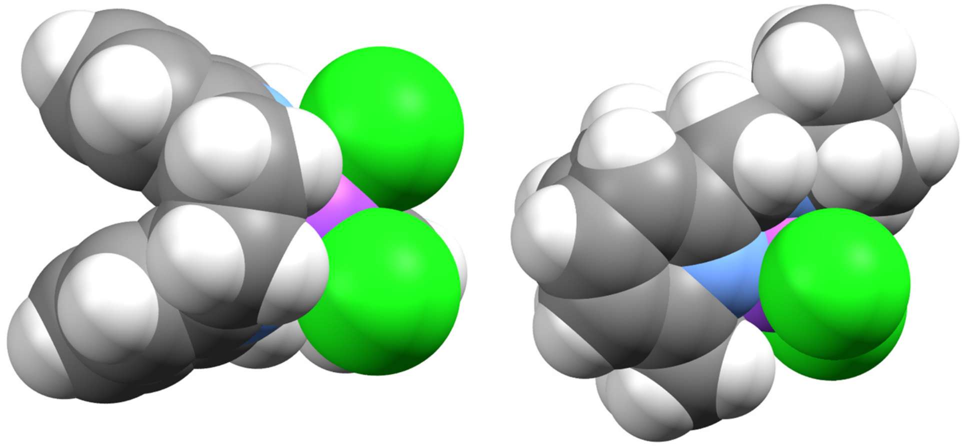

Supplementary Figure 59. The space filling model of **(**$(^{iPr}N3)Ni^{II}Cl_2$ (**1b)**

1.15 X-ray photoelectron spectroscopy (XPS) experiments

Solid samples of (^{iPr}N3)Ni^{II}(PhAc)Br (**4**) and [(^{iPr}N3)Ni^{III}(PhAc](Br)]PF$_6$ (**5**) were deposited on a piece of tape pre-fixed to the XPS metal plate at room temperature inside of N_2-filled glovebox. The XPS metal plate was stored under N_2 and quickly loaded into the instrument to reduce the exposure of samples to the air. XPS spectra were recorded at room temperature over several hours, using Kratos Axis ULTRA X-ray Photoelectron Spectrometer at the Materials Research Laboratory at UIUC. Obtained spectra were analyzed by software CasaXPS.

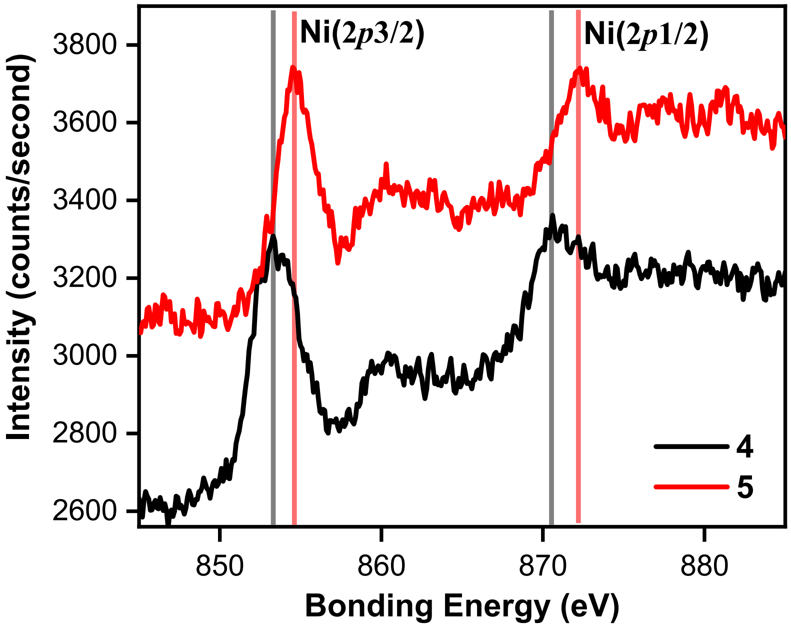

Supplementary Figure 60. X-ray photoelectron spectra showing the Ni binding energy region for **4** (black line) and **5** (red line). Selected bonding energies (eV): **4**: 2*p*3/2, 853.3; 2*p*1/2, 870.6; **5**: 2*p*3/2, 854.5; 2*p*1/2, 872.1.

1.16 DFT calculations

The density functional theory (DFT) and time-dependent density functional theory (TD-DFT) calculations were performed using software package Gaussian 16.[19] Single point energy, EPR parameters, and TD-DFT calculations were performed using the crystallographic coordinates for **1a** and **1b**, and including the experimentally determined triplet ground state (S = 1) for both complexes. The M06[20] functional along with the tzvp[21,22] basis set were employed, since this combination of hybrid functional and basis set has been previously shown to work well for reproducing experimental parameters for Ni complexes.[23,24] In addition, the B3LYP[25,26] functional, the long-range corrected functionals CAM-B3LYP[27] and ωB97xD,[28] and the def2tzvp[29,30] basis set were also employed to probe their effect on the TD-DFT calculated UV-vis spectra and the nature of the electronic transitions (Supplementary Fig. 73); while the predicted transition energies vary slightly depending on the functional/basis set combination, inspection of the NTOs corresponding to the transitions at 370–400 nm and 500–600 nm reveal that these are mainly charge transfer and d-d transitions, respectively, for all functional/basis set combinations. The atomic contributions to frontier molecular orbitals, the calculated UV-Vis spectra, and the natural transition orbitals (NTOs) were analyzed using the program Chemissian.[31] The excited states of interest were investigated in more detail for spin contamination and were confirmed to be triplet excited states. For the simulated UV-vis spectra, Gaussian peak shapes with a full width at half maximum (FWHM) of 0.33 eV were used.

Supplementary Table 15. Cartesian coordinates for $(^{Me}N3)Ni^{II}Cl_2$ (**1a**)

Atom type	x	y	z
Ni	9.9776	3.3204	2.7555
Cl	10.8915	1.5874	1.5092
N	8.7164	4.5833	3.7799
N	11.0144	3.3204	4.5306
C	9.1737	4.9228	4.994
C	7.4096	4.7386	3.4787
C	10.6163	4.5662	5.2545
H	11.1959	5.314	4.9628
H	10.7507	4.4374	6.2269
C	8.3625	5.5457	5.9306
H	8.7015	5.7894	6.7843
C	6.5489	5.3776	4.3626

H	5.6383	5.5198	4.1315
C	6.9444	4.1072	2.1979
H	7.5259	4.4228	1.4614
H	6.0256	4.4228	2.0069
C	12.4829	3.3204	4.3603
C	7.0392	5.8053	5.5885
H	6.4739	6.2729	6.1916
H	12.7465	4.0814	3.8637
H	12.9462	3.3204	5.2216
N	8.7164	2.0575	3.7799
C	9.1737	1.718	4.994
C	7.4096	1.9022	3.4787
C	10.6163	2.0746	5.2545
H	11.1959	1.3268	4.9628
H	10.7507	2.2034	6.2269
C	8.3625	1.0951	5.9306
H	8.7015	0.8514	6.7843
C	6.5489	1.2632	4.3626
H	5.6383	1.121	4.1315
C	6.9444	2.5336	2.1979
H	7.5259	2.218	1.4614
H	6.0256	2.218	2.0069
C	7.0392	0.8355	5.5885
H	6.4739	0.3679	6.1916
H	12.7465	2.5594	3.8637
Cl	10.8915	5.0534	1.5092

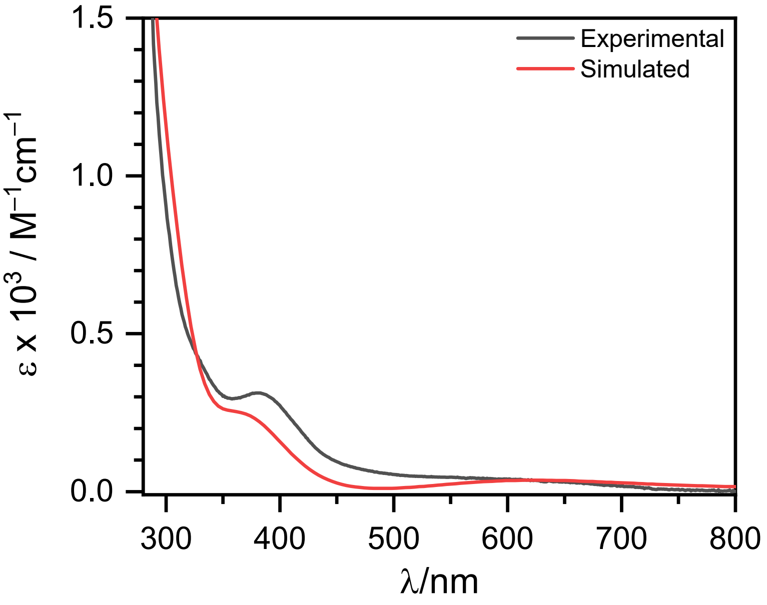

Supplementary Figure 61. Overlaid experimental and TD-DFT (M06/tzvp) calculated UV-vis absorption spectrum of (^{Me}N3)Ni^{II}Cl$_2$ (**1a**).

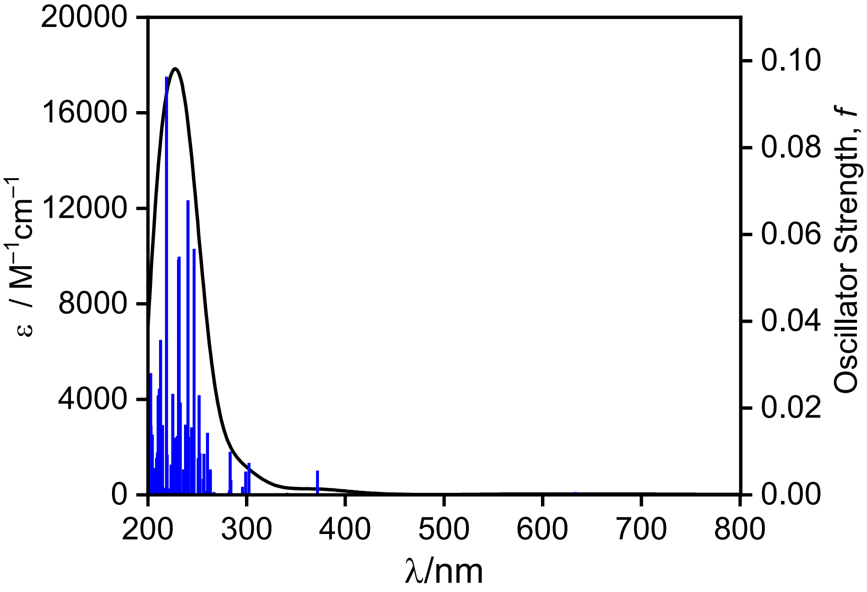

Supplementary Figure 62. Relevant oscillator (solid blue bars) from TD-DFT (M06/tzvp) for (^{Me}N3)Ni^{II}Cl$_2$ (**1a**), with the simulated absorption spectrum overlaid (solid black line).

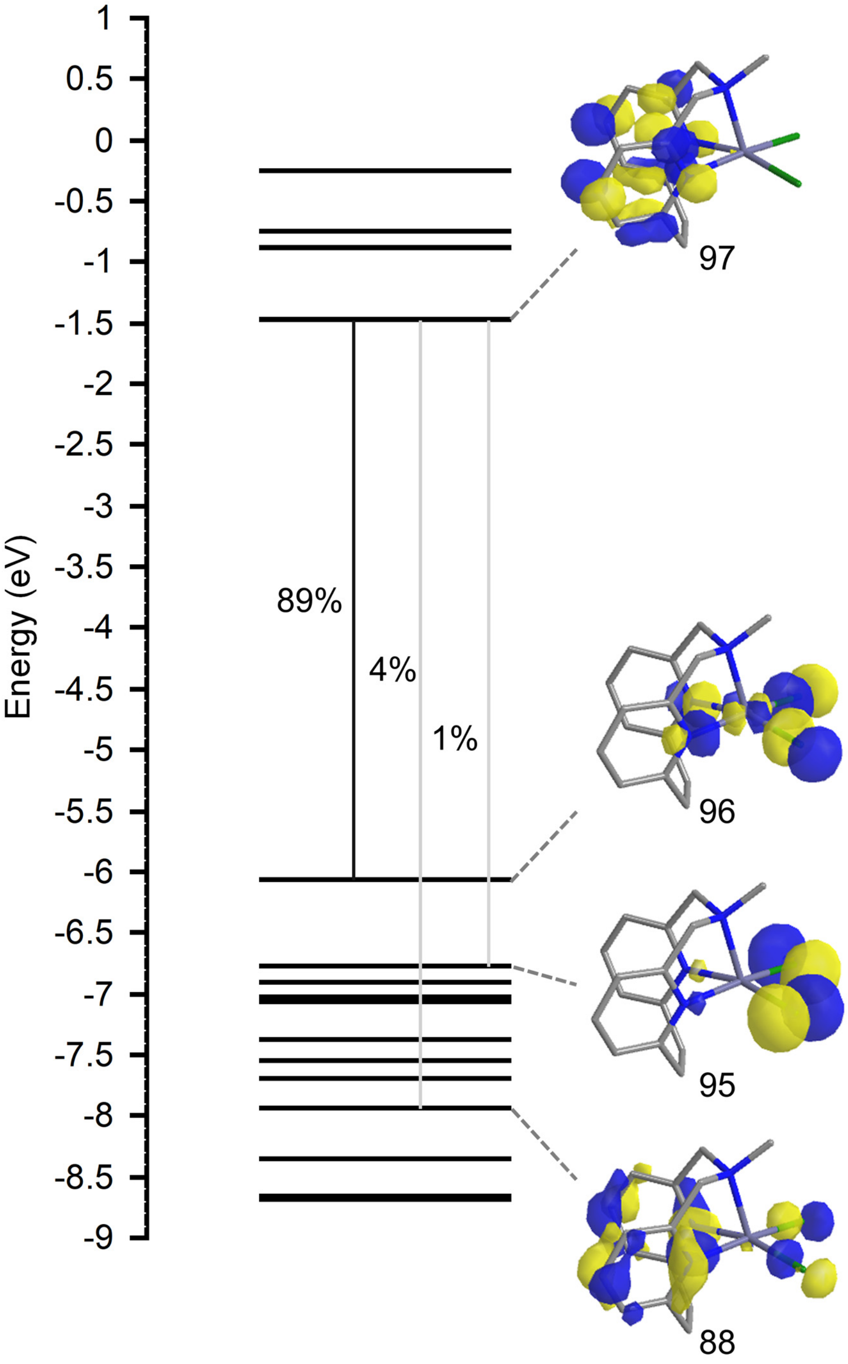

Supplementary Figure 63. Frontier molecular orbital (α-MO) diagram for (^{Me}N3)Ni^{II}Cl$_2$ (**1a**) corresponding to 371 nm excitation as determined by TD-DFT (M06/tzvp).

Supplementary Table 16. Atomic contributions of selected molecular orbitals for (^{Me}N3)Ni^{II}Cl$_2$ (**1a**), as determined by TD-DFT (M06/tzvp).

MO #	MOs (0.05 isocontour value)	Ni	Cl	N
97<br>α-LUMO	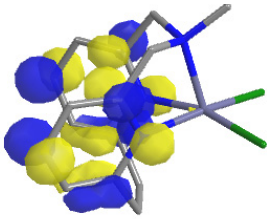	0%	0%	18%
96<br>α-HOMO	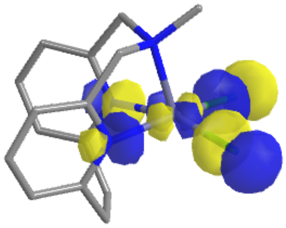	22%	50%	20%
95<br>α-HOMO–1	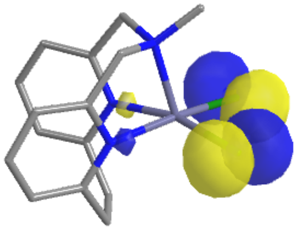	0%	94%	4%
88<br>α-HOMO–8	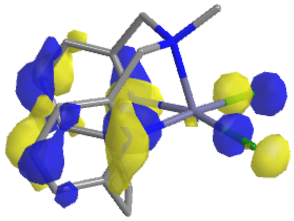	0%	20%	32%

Supplementary Table 17. Selected vertical electronic excitation of (^{Me}N3)Ni^{II}Cl$_2$ (**1a**) with oscillator strength[a], as determined by TD-DFT (M06/tzvp).

Wavelength (nm)	Osc. Strength (*f*)	<S^2> values	Major contributions
**632.570**	0.0006	2.006	β – β amplitudes (percentage contribution)<br>92b → 97b 0.43 (19%)<br>83b → 97b –0.40 (16%)<br>85b → 97b –0.34 (11%)<br>92b → 99b –0.31 (10%)<br>93b → 97b 0.30 (9%)<br>83b → 99b 0.30 (9%)<br>85b → 99b 0.25 (7%)<br>88b → 97b 0.24 (6%)<br>93b → 99b –0.21 (5%)<br>88b → 99b –0.18 (3%)<br>91b → 95b 0.15 (2%)<br>87b → 95b 0.11 (1%)
**371.140**	0.0055	2.454	α – α amplitudes (percentage contribution)<br>96a → 97a 0.94 (89%)<br>88a → 97a 0.19 (4%)<br>95a → 97a 0.10 (1%)
301.690	0.0073	2.085	β – β amplitudes (percentage contribution)<br>92b → 95b 0.67 (45%)<br>93b → 95b 0.36 (13%)<br>83b → 95b 0.36 (13%)<br>85b → 95b 0.30 (9%)<br>88b → 95b –0.28 (8%)<br>83b → 96b 0.18 (3%)<br>92b → 96b 0.17 (3%)<br>85b → 96b 0.15 (2%)<br>88b → 96b –0.14 (2%)

[a]Calculated transitions with energies less than 300 nm and oscillator strengths greater than 0.001 are listed.

Supplementary Table 18. TD-DFT (M06/tzvp) calculated transitions for $^{Me}N3Ni^{II}Cl_2$ (**1a**).

Wavelength (nm)	Oscillator strength	Wavelength (nm)	Oscillator strength
**1890.29**	0.0001	237.53	0.0161
**1705.91**	0.0001	235.38	0.0058
**1209.06**	0	235.38	0.0031
**1073.8**	0.0003	232.88	0.0005
**632.57**	0.0006	232.25	0.0212
**602.27**	0.0003	232.1	0.0001
**371.14**	0.0055	231.05	0
**343.15**	0	230.98	0.0548
**340.47**	0.0004	230.33	0.0541
**336.22**	0	229	0.0135
**314.23**	0.0001	228.86	0
**312.3**	0	227.8	0.001
**304.11**	0	226.9	0.0131
**301.69**	0.0073	226.74	0.0012
**298.54**	0.0053	226.66	0.0049
**296.18**	0	226.35	0.0025
**295.21**	0.0018	225.35	0.0103
**286.89**	0.0002	224.51	0.0232
**285.42**	0.0001	222.84	0.0069
**283.3**	0.0033	222.68	0.0035
**282.74**	0.0099	221.52	0.0002
**282.08**	0.001	221.24	0.0014
**281.32**	0.0005	219.43	0.0013
**279.12**	0	219.09	0.0012
**274.68**	0.0002	218.84	0.0092
**270.14**	0	218.28	0.0963
**267.28**	0.0001	217.39	0.0008
**266.27**	0.0006	216.01	0.0008
**264.64**	0	215.66	0.0016
**263.2**	0.0001	215.2	0
**262.6**	0.0058	214.17	0.016
**261.14**	0.0004	212.2	0.0356
**259.69**	0.0142	211.24	0.0005
**258.47**	0.0008	210.98	0.0145
**256.11**	0.0094	210.82	0.0244
**255.44**	0.0036	209.82	0.0229
**252.03**	0.0095	209.67	0.0033
**251.25**	0.0229	208.98	0.0097

**250.9**	0.0028	208.16	0.0085
**250.26**	0.0084	207.79	0.0001
**248.17**	0.0002	207.42	0.0015
**246.73**	0.0002	206.13	0.0001
**246.21**	0.0566	205.93	0.0038
**244.57**	0.0005	205.44	0.0061
**243.76**	0	205.21	0.0005
**243.71**	0.0155	204.26	0.0036
**240.46**	0.0133	203.78	0.0138
**240.15**	0	202.42	0.0159
**239.92**	0.0678	202.09	0.028
**238.83**	0.0003	201.12	0.0087

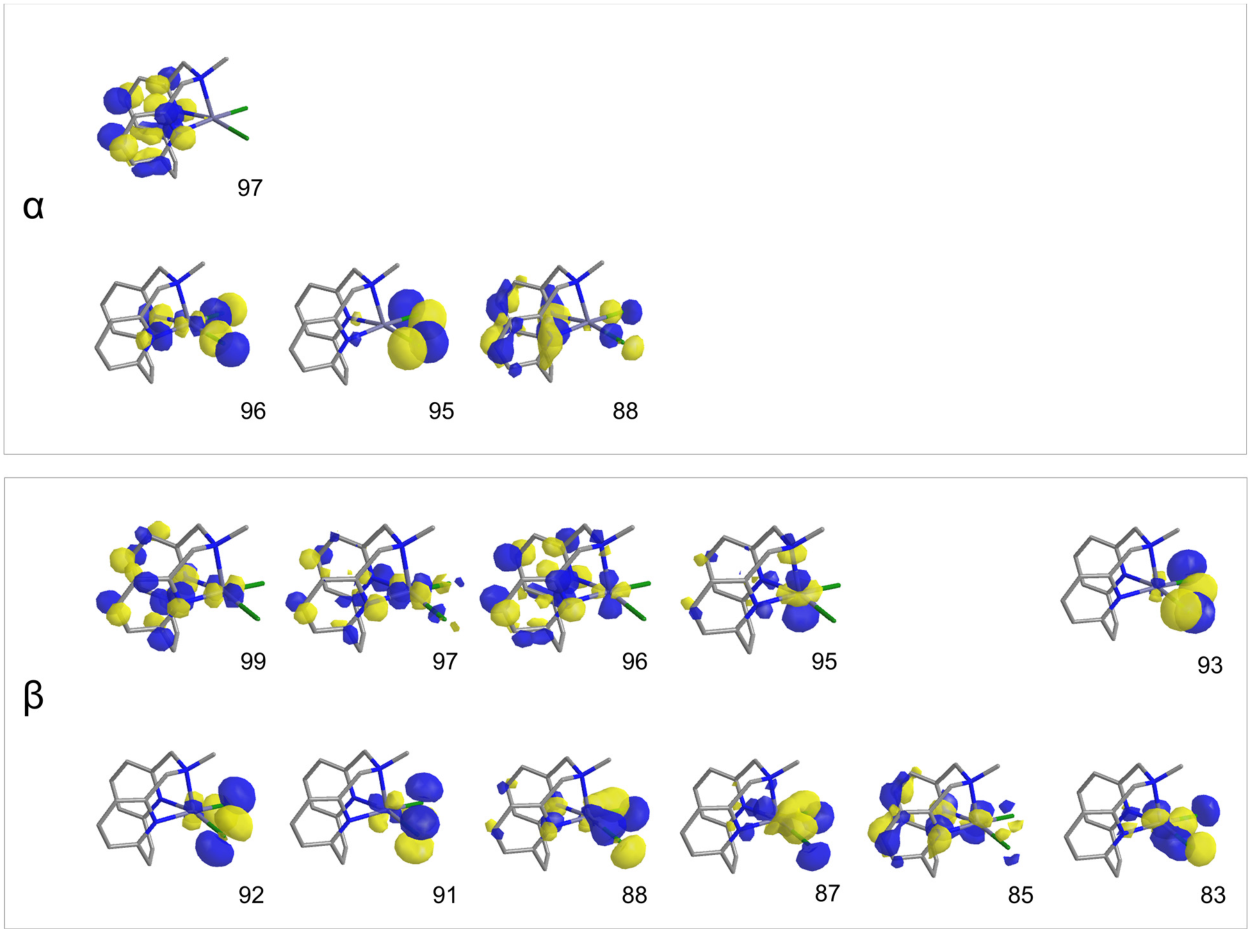

Supplementary Figure 64. Frontier molecular orbitals for (^{Me}N3)Ni^{II}Cl$_2$ (**1a**), determined by TD-DFT (M06/tzvp).

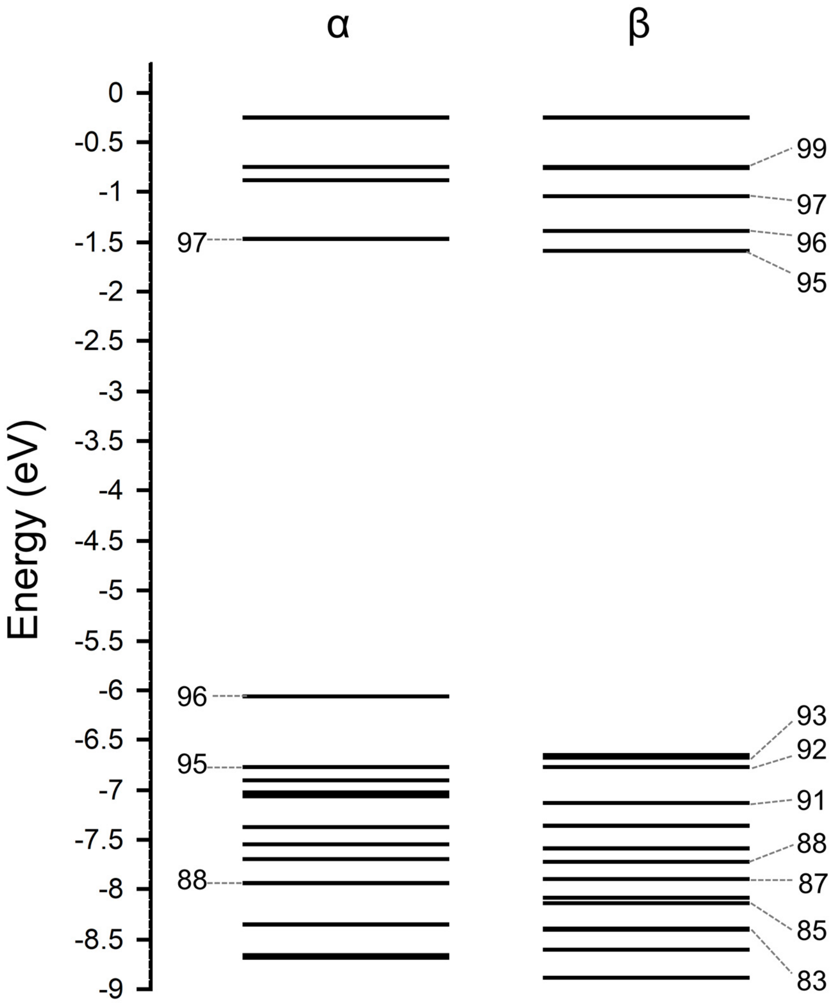

Supplementary Figure 65. Energy levels for molecular orbitals of $(^{Me}N3)Ni^{II}Cl_2$ (**1a**), determined by TD-DFT (M06/tzvp).

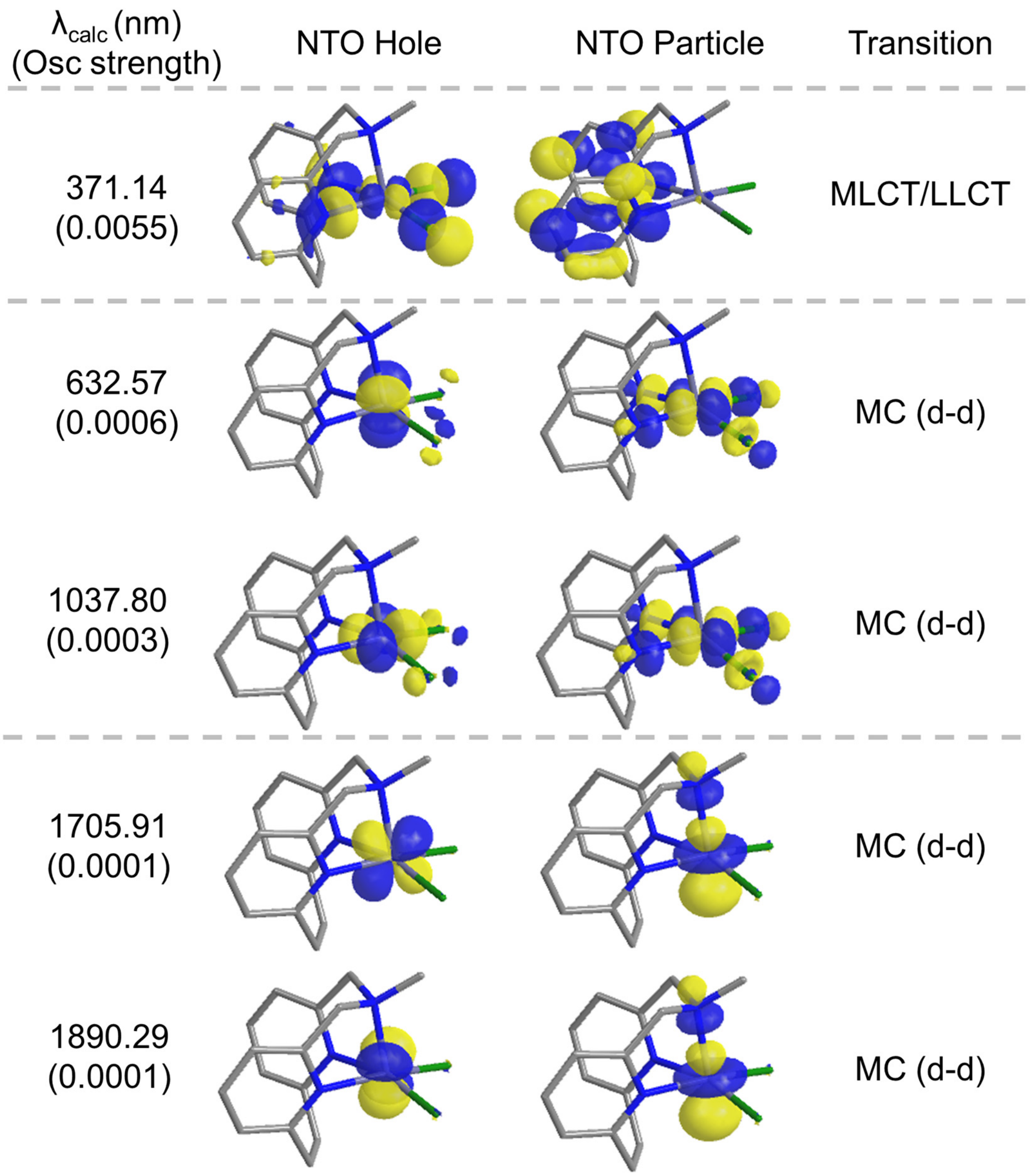

Supplementary Figure 66. Natural transition orbitals (NTOs) of (^{Me}N3)$Ni^{II}Cl_2$ (**1a**), illustrating the nature of optically active excited states corresponding to the transitions of interest with respect to photoreactivity. Calculations were carried out using the M06/tzvp functional/basis set combination.

Supplementary Table 19. Cartesian coordinates for (^{iPr}N3)Ni^{II}Cl$_2$ (**1b**)

Atom type	x	y	z
Ni	6.85195	8.26666	9.14188
Cl	7.95953	7.16038	7.4019
Cl	8.26941	7.61786	10.83963
N	6.15223	9.63428	7.82373
N	4.95231	7.42887	8.93776
N	5.85253	9.62537	10.32727
C	4.93683	9.35365	7.34996
C	4.46681	7.94804	7.61864
H	3.47701	7.92918	7.6065
H	4.79195	7.35331	6.89779
C	4.7444	11.57704	6.51674
H	4.26996	12.2409	6.02889
C	4.20139	10.30925	6.67304
H	3.3432	10.10258	6.32257
C	6.6698	10.88351	7.74436
C	5.97307	11.87185	7.0689
H	6.33913	12.74515	6.98759
C	4.12619	7.95403	10.0683
H	4.19807	7.33723	10.83938
H	3.1762	7.98472	9.79088
C	4.93461	5.91305	8.88631
H	5.56278	5.64324	8.15745
C	4.56745	9.33919	10.49474
C	6.3148	10.88936	10.54182
C	7.91617	11.09998	8.53753
H	8.3508	11.9325	8.22298
H	8.53991	10.35105	8.3589
C	3.57102	5.34069	8.52564
H	2.91189	5.61839	9.19624
H	3.62299	4.36141	8.50452
H	3.30117	5.67101	7.64291

C	5.46103	5.29231	10.16829
H	6.28163	5.75286	10.44134
H	5.65346	4.34388	10.0166
H	4.78642	5.38015	10.87336
C	3.66723	10.27359	10.99957
H	2.75042	10.05873	11.12335
C	4.16379	11.53641	11.31606
H	3.59313	12.18536	11.71071
C	5.47099	11.84773	11.05952
H	5.79613	12.72323	11.23743
C	7.69388	11.20463	10.07655
H	8.32426	10.58929	10.52629
H	7.91839	12.12543	10.36367

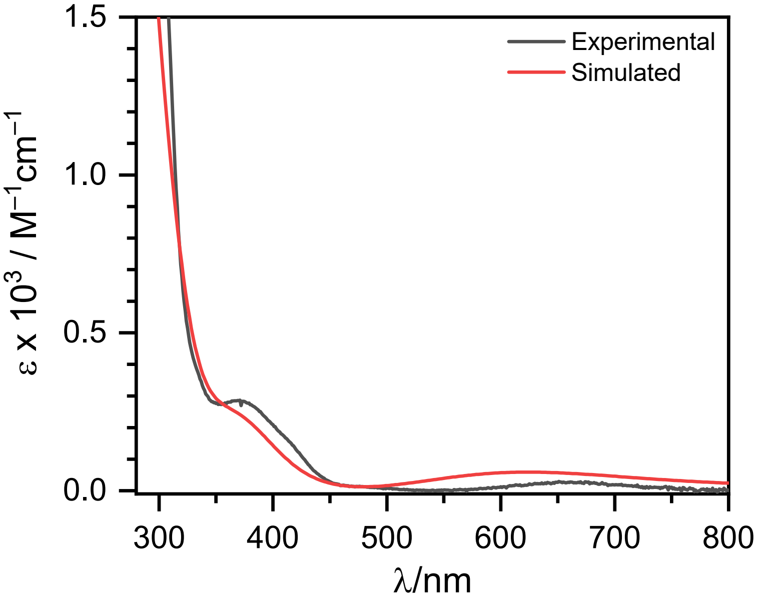

Supplementary Figure 67. Overlaid experimental and TD-DFT (M06/tzvp) calculated UV-vis absorption spectrum of (^{iPr}N3)Ni^{II}Cl$_2$ (**1b**).

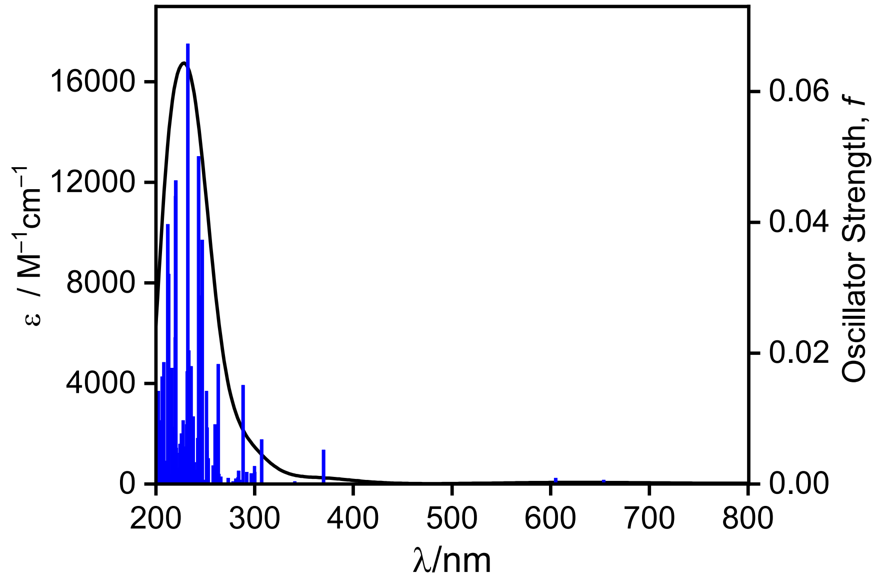

Supplementary Figure 68. Relevant oscillators (solid blue bars) from TD-DFT (M06/tzvp) for (^{iPr}N3)Ni^{II}Cl$_2$ (**1b**), with the simulated absorption spectrum overlaid (solid black line).

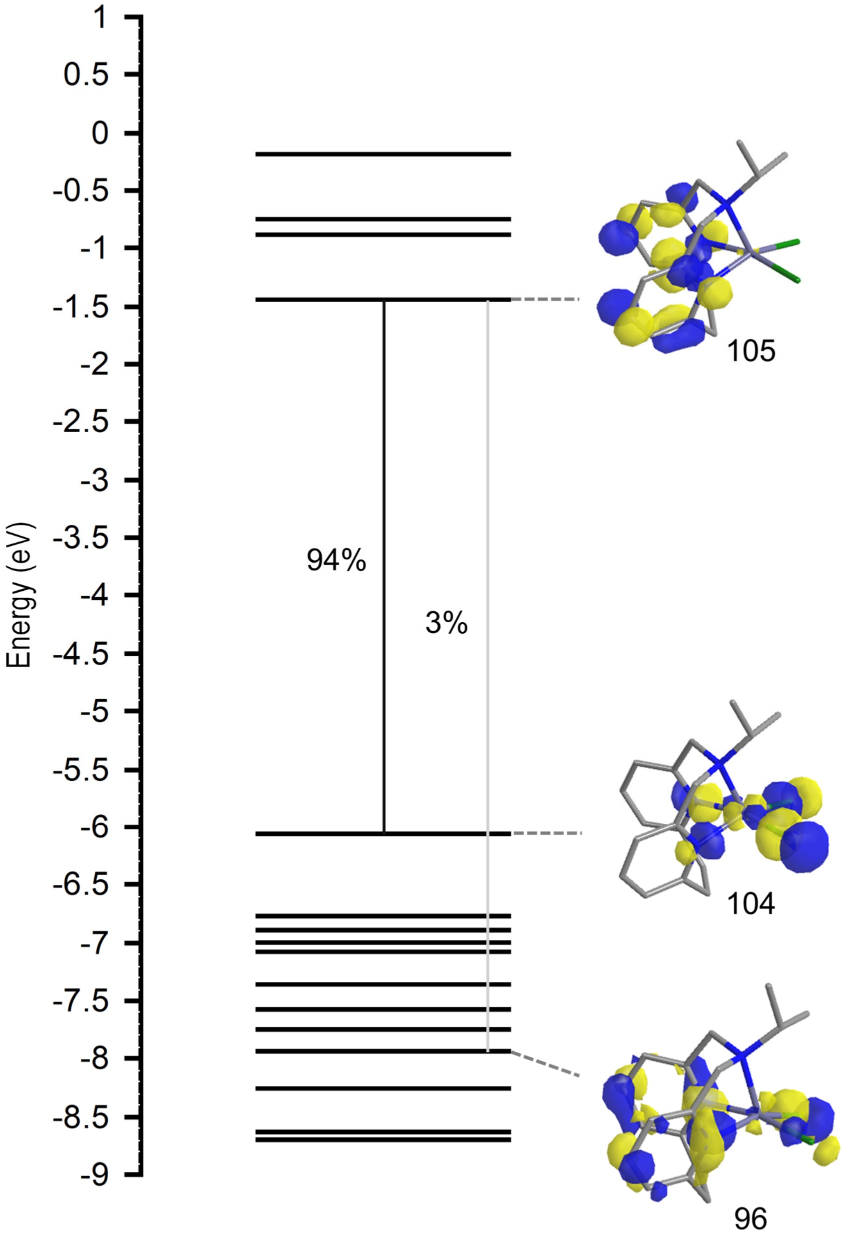

Supplementary Figure 69. Frontier molecular orbital (α-MO) diagram for (^{iPr}N3)Ni^{II}Cl$_2$ (**1b**) corresponding to 369 nm excitation as determined by TD-DFT (M06/tzvp).

Supplementary Table 20. Atomic contributions of selected molecular orbitals for (^{iPr}N3)Ni^{II}Cl$_2$ (**1b**) determined by TD-DFT (M06/tzvp).

MO #	MOs (0.05 isocontour value)	Ni	Cl	N
105 α-LUMO	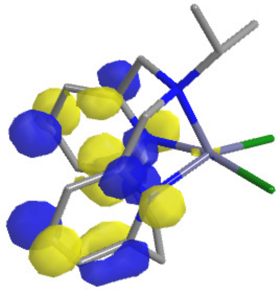	1%	0%	19%
105 α-LUMO	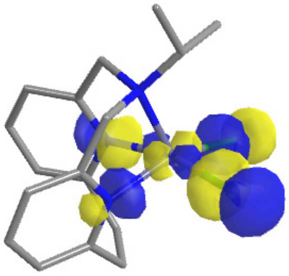	22%	48%	21%
104 α-HOMO	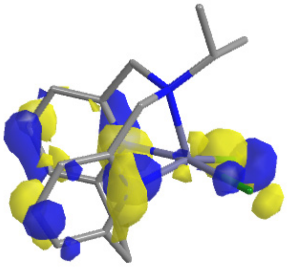	6%	23%	30%

Supplementary Table 21. Selected TD-DFT calculated vertical electronic excitation of (^{iPr}N3)$Ni^{II}Cl_2$ (**1b**) with oscillator strength[a], determined by TD-DFT (M06/tzvp).

Wavelength (nm)	Osc. Strength (*f*)	<S^2> values	Major contributions
**653.01**	0.0006	2.007	β – β amplitudes (percentage contribution)<br>91b → 105b 0.38 (15%)<br>100b → 105b 0.38 (14%)<br>94b → 105b –0.28 (8%)<br>102b → 105b 0.27 (7%)<br>101b → 105b 0.25 (6%)<br>93b → 105b 0.24 (6%)<br>91b → 107b –0.22 (5%)<br>97b → 105b 0.21 (4%)<br>100b → 107b –0.20 (4%)<br>94b → 107b 0.16 (3%)<br>102b → 107b –0.14 (2%)<br>93b → 107b –0.14 (2%)<br>101b → 107b –0.13 (2%)<br>91b → 106b 0.13 (2%)<br>94b → 103b –0.12 (2%)<br>100b → 103b 0.12 (2%)<br>99b → 103b 0.12 (2%)<br>100b → 106b 0.12 (2%)<br>89b → 105b 0.12 (1%)<br>91b → 103b 0.12 (1%)<br>97b → 107b –0.11 (1%)<br>90b → 105b –0.10 (1%)
604.44	0.0009	2.006	β – β amplitudes (percentage contribution)<br>99b → 105b 0.54 (29%)<br>90b → 105b 0.32 (10%)<br>97b → 105b –0.29 (9%)<br>99b → 107b –0.28 (8%)<br>94b → 105b –0.25 (6%)<br>95b → 105b 0.25 (6%)<br>92b → 105b 0.19 (4%)<br>99b → 106b 0.17 (3%)<br>90b → 107b –0.17 (3%)<br>97b → 107b 0.15 (2%)<br>94b → 107b 0.14 (2%)<br>95b → 107b –0.13 (2%)

			102b → 105b –0.12 (1%)<br>89b → 105b –0.11 (1%)<br>90b → 106b 0.11 (1%)<br>101b → 105b –0.10 (1%)<br>92b → 107b –0.10 (1%)
**369.21**	0.0052	2.447	α – α amplitudes (percentage contribution)<br>104a → 105a 0.94 (89%)<br>96a → 105a 0.18 (3%)
306.54	0.0068	2.057	β – β amplitudes (percentage contribution)<br>100b → 103b 0.56 (32%)<br>101b → 103b 0.52 (27%)<br>94b → 103b 0.37 (14%)<br>91b → 103b –0.31 (10%)<br>97b → 103b –0.27 (7%)<br>94b → 104b –0.12 (1%)<br>100b → 104b –0.11 (1%)<br>90b → 103b 0.10 (1%)

[a]Calculated transitions with energies less than 300 nm and oscillator strengths greater than 0.001 are listed.

Supplementary Table 22. TD-DFT calculated transitions for (^{iPr}N3)Ni^{II}Cl$_2$ (**1b**) using the M06/tzvp functional/basis set combination.

Wavelength (nm)	Oscillator strength	Wavelength (nm)	Oscillator strength
**2202.92**	0	238.41	0.0033
**1714.01**	0.0001	237.18	0.0103
**1286.9**	0.0001	235.38	0.0069
**993.65**	0.0002	235.04	0.018
**653.01**	0.0006	233.22	0.0046
**604.44**	0.0009	232.74	0.0204
**369.21**	0.0052	231.66	0.0673
**341.6**	0.0001	231.09	0.0172
**340.08**	0.0004	230.53	0.0091
**335.83**	0	229.81	0.0008
**315.08**	0.0001	228.77	0.0026
**309.41**	0	228.16	0.0057
**306.54**	0.0068	227.25	0.0025
**299.53**	0.0018	226.92	0.0097
**299.25**	0.0027	226.04	0.0024
**297**	0.0001	225.6	0.0044
**296.03**	0.0016	225.19	0.0077
**291.39**	0.0018	223.84	0.0061
**287.58**	0.0151	223.2	0.0047
**284.4**	0.0006	221.96	0.0025
**283.07**	0.002	221.36	0.0002
**282.47**	0.001	219.94	0.0055
**280.38**	0.0008	219.48	0.0464
**277.5**	0.0004	219.18	0.044
**272.63**	0.0009	218.87	0.0224
**272.47**	0	217.81	0.0087
**268.69**	0.0001	216.72	0.0033
**265.25**	0.0011	216.43	0.0016
**264.13**	0.0002	215.94	0.0012
**263.1**	0.0015	215.52	0.0177
**262.55**	0.0183	214.94	0.0009
**261.03**	0.0085	213.74	0.0014
**260.22**	0.0082	212.26	0.0321
**259.61**	0.0023	211.42	0.0397
**259.37**	0.0091	209.73	0.0028
**257.29**	0.0028	208.82	0.0022
**252.4**	0.0039	208.74	0.0035

**251.25**	0.0086	208.16	0.0004
**251.05**	0.0051	207.46	0.0186
**250.61**	0.0142	206.68	0.0068
**248.75**	0.0006	205.73	0.0032
**247.96**	0.0002	205.59	0.0164
**246.39**	0.0373	204.56	0.0027
**244.58**	0.0001	203.81	0.002
**243.58**	0.0288	203.78	0.0079
**243.38**	0.0206	203.37	0.0097
**242.59**	0.0501	202.85	0.0063
**241.75**	0.007	202.06	0.0069
**240.66**	0.0021	201.79	0.0142
**238.61**	0.0012	201.31	0.0016

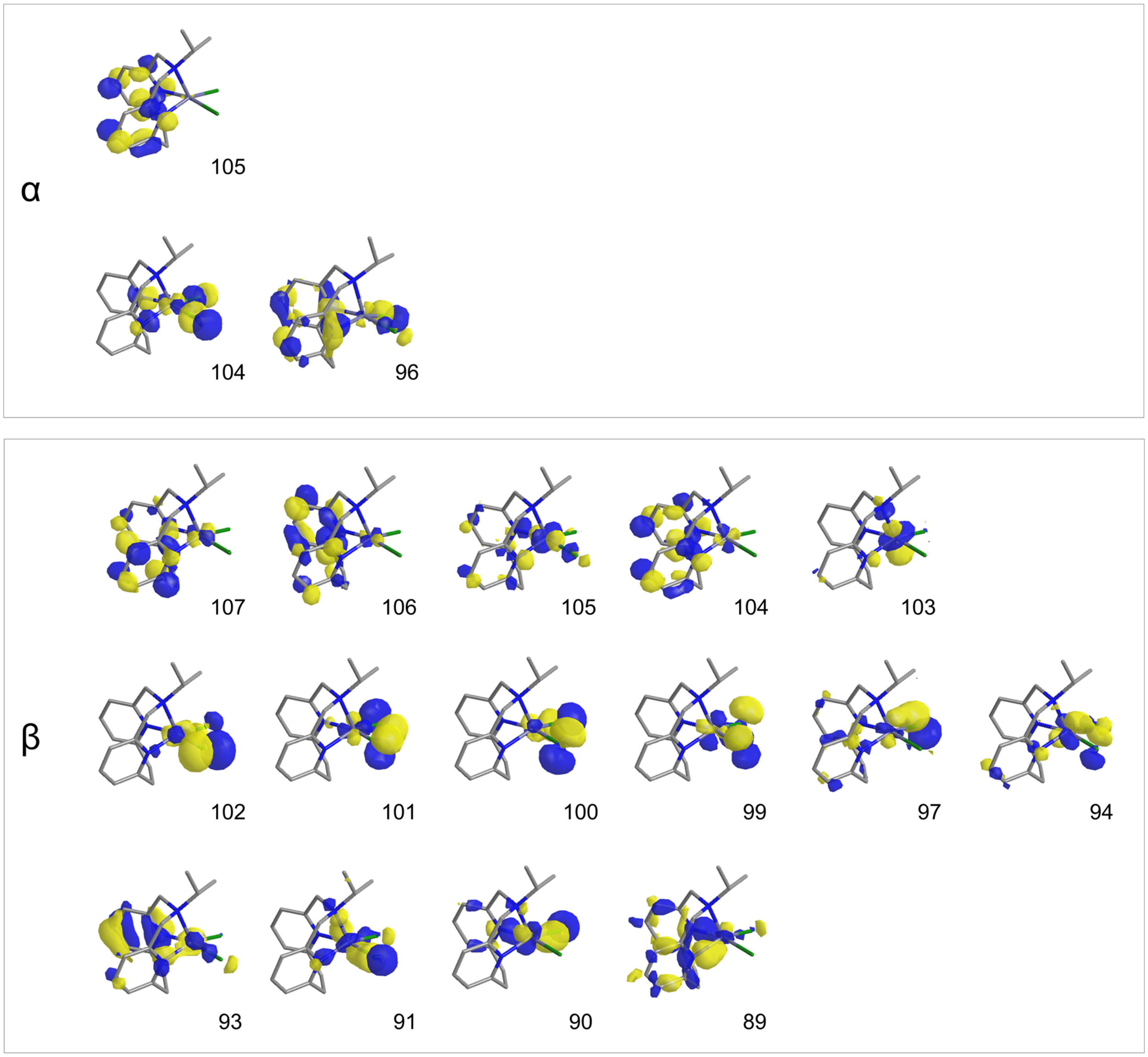

Supplementary Figure 70. Frontier molecular orbitals for (^{iPr}N3)$Ni^{II}Cl_2$ (**1b**), determined by TD-DFT (M06/tzvp).

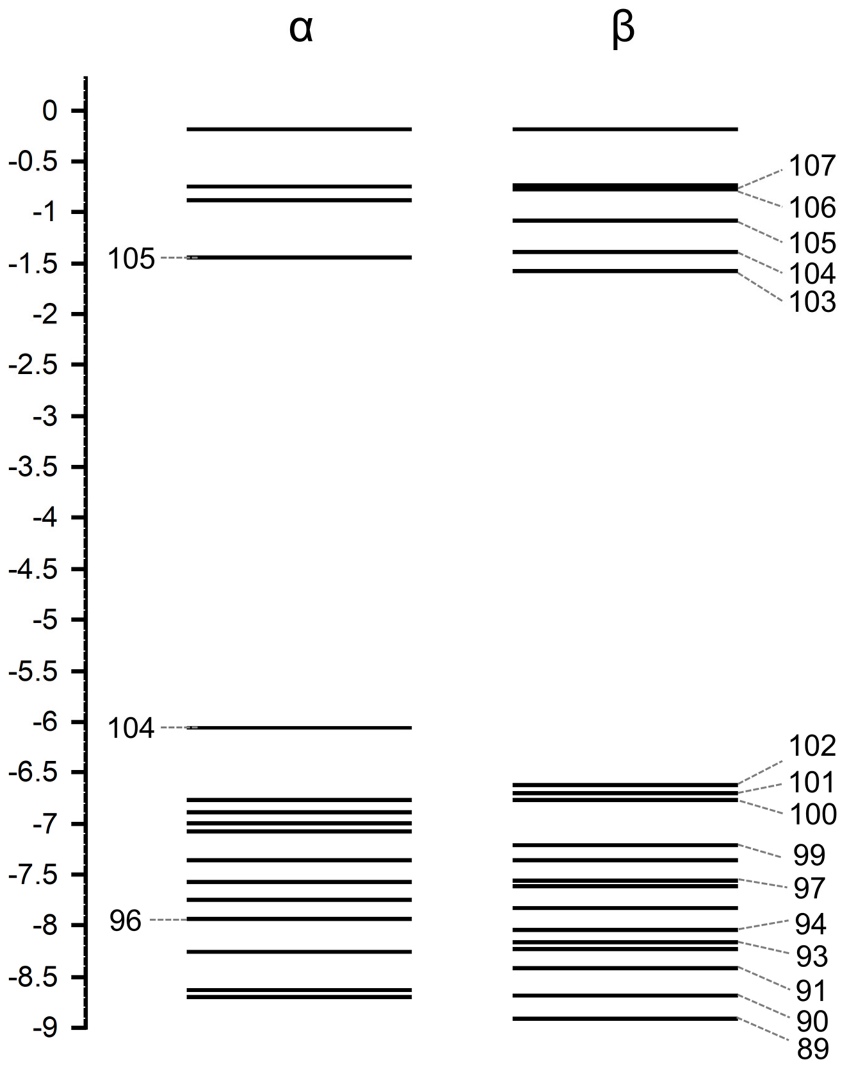

Supplementary Figure 71. Energy levels for molecular orbitals of (^{iPr}N3)$Ni^{II}Cl_2$ (**1b**), determined by TD-DFT (M06/tzvp).

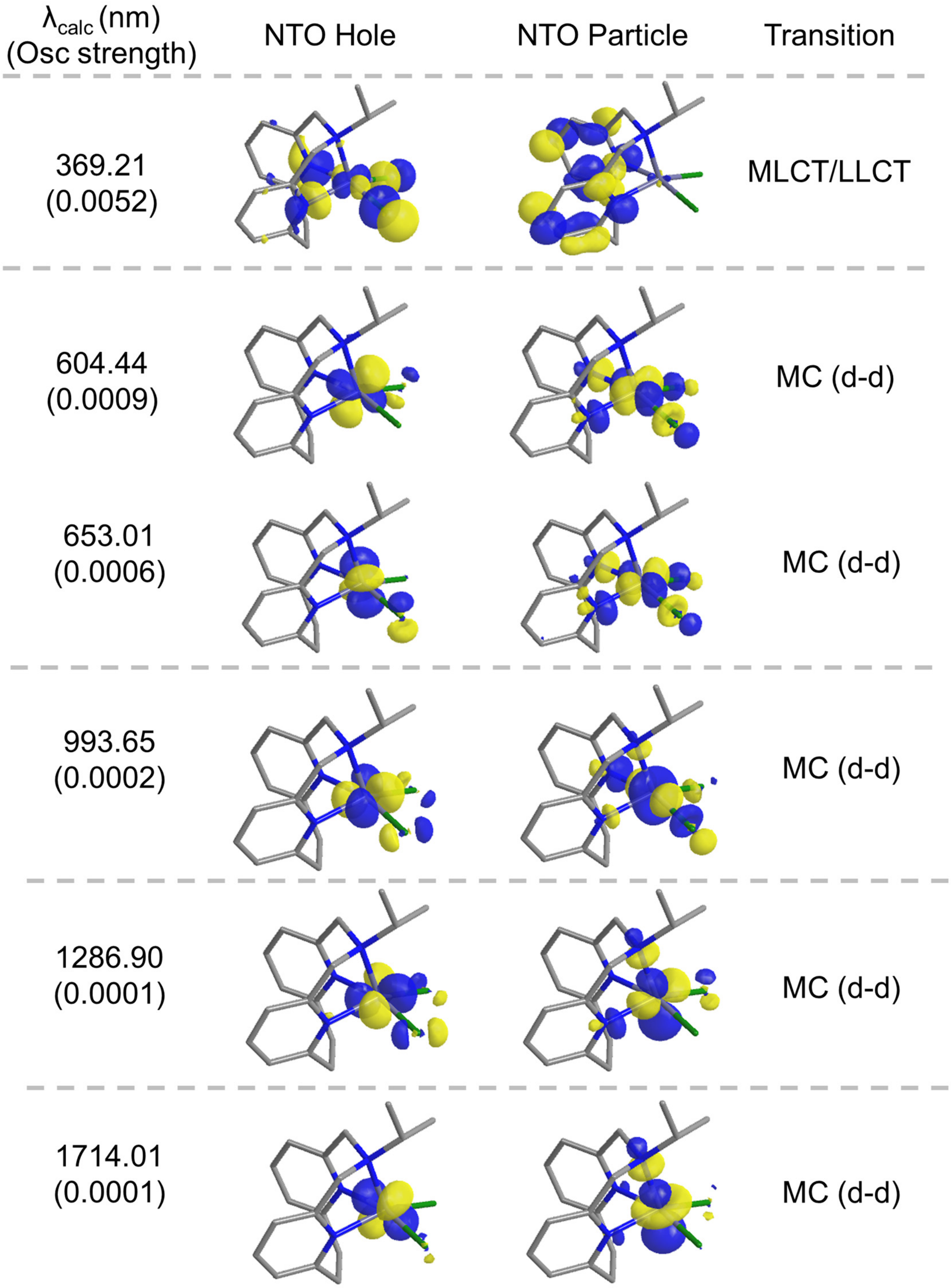

Supplementary Figure 72. Natural transition orbitals (NTOs) of (^{iPr}N3)$Ni^{II}Cl_2$ (**1b**), illustrating the nature of optically active excited states corresponding to the transitions of interest with respect to photoreactivity. Calculations were carried out using the M06/tzvp functional/basis set combination.

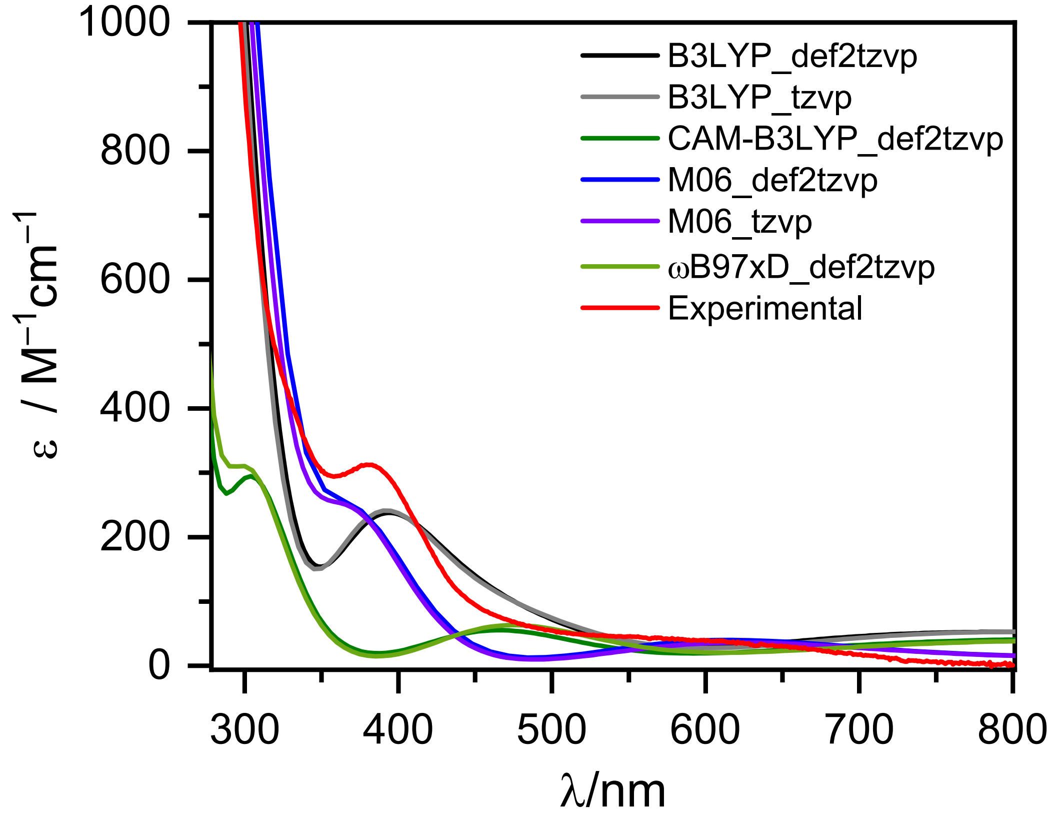

Supplementary Figure 73. Calculated UV-vis spectra for (^{Me}N3)$Ni^{II}Cl_2$ (**1a**) based on TD-DFT calculations employing various functional/basis set combinations. While the predicted transition energies vary slightly depending on the functional/basis set combination, inspection of the NTOs corresponding to the transitions at 370–400 nm and > 450 nm reveal that these are mainly charge transfer and d-d transitions, respectively, for all functional/basis set combinations (Supplementary Figs. 74–78).

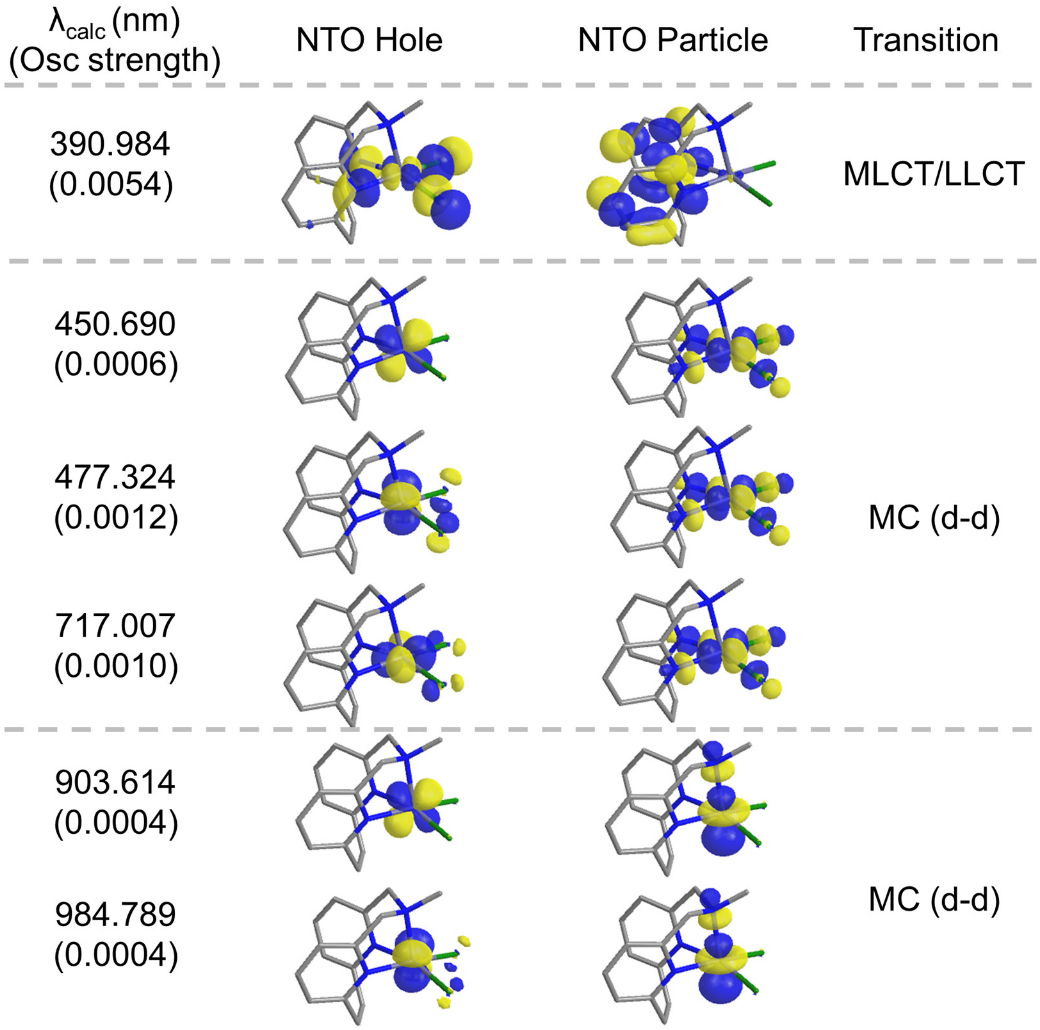

Supplementary Figure 74. Natural transition orbitals (NTOs) of (^{Me}N3)Ni^{II}Cl$_2$ (**1a**). The nature of optically active excited states corresponding to the transitions of interest are shown based on TD-DFT calculations employing the B3LYP/def2tzvp functional/basis set combination.

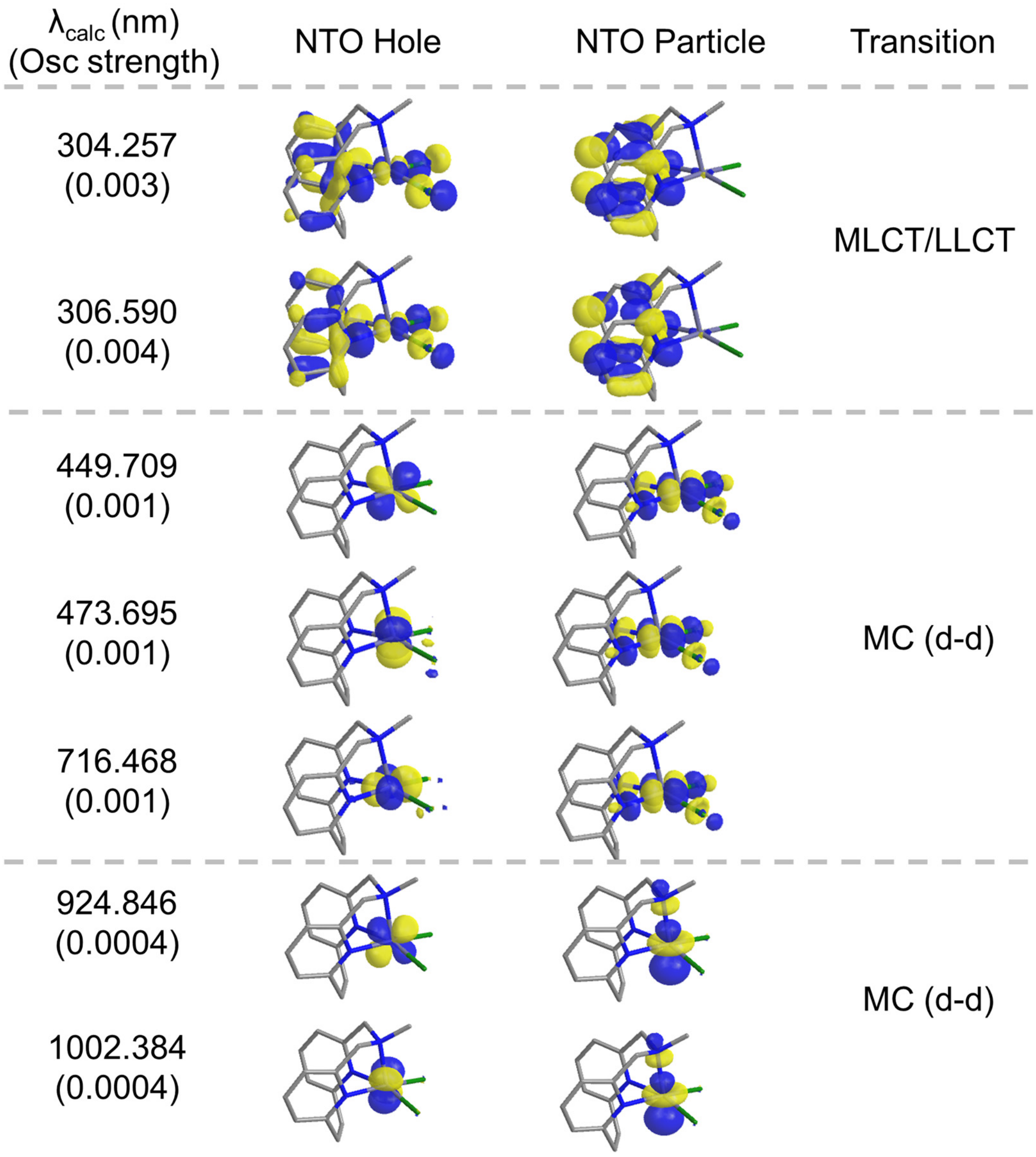

Supplementary Figure 75. Natural transition orbitals (NTOs) of (^{Me}N3)$Ni^{II}Cl_2$ (**1a**). The nature of optically active excited states corresponding to the transitions of interest are shown based on TD-DFT calculations employing the B3LYP/tzvp functional/basis set combination.

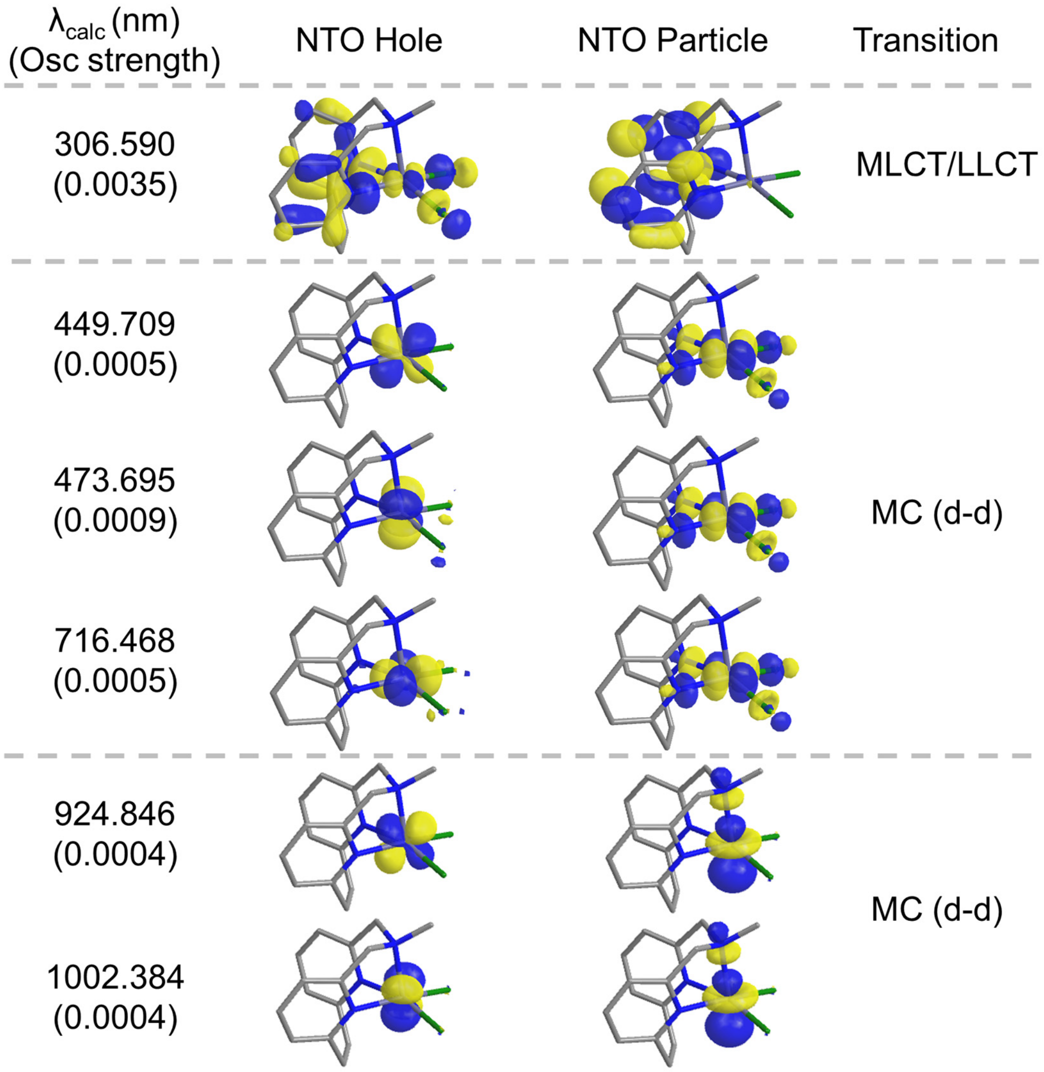

Supplementary Figure 76. Natural transition orbitals (NTOs) of (^{Me}N3)$Ni^{II}Cl_2$ (**1a**). The nature of optically active excited states corresponding to the transitions of interest are shown based on TD-DFT calculations employing the CAM-B3LYP/def2tzvp functional/basis set combination.

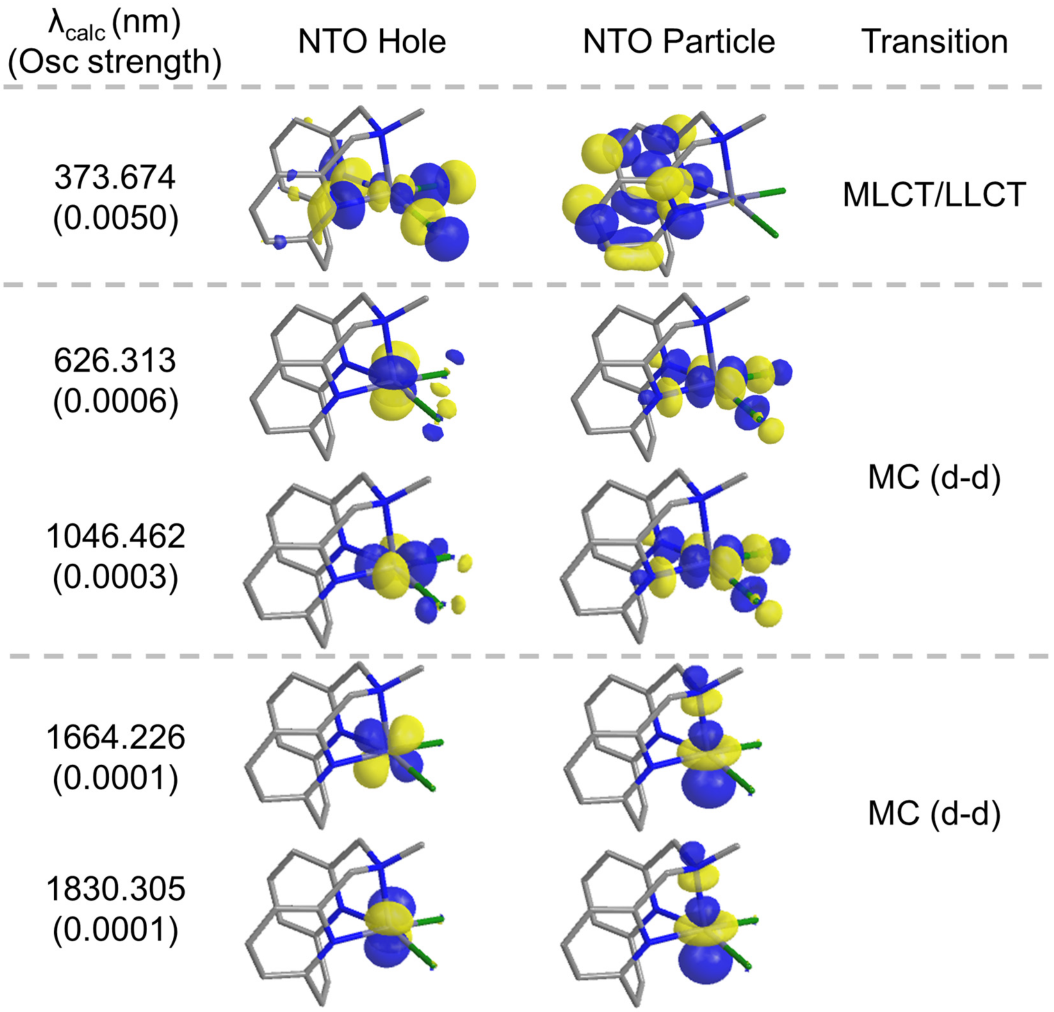

Supplementary Figure 77. Natural transition orbitals (NTOs) of (^{Me}N3)Ni^{II}Cl$_2$ (**1a**). The nature of optically active excited states corresponding to the transitions of interest are shown based on TD-DFT calculations employing the M06/def2tzvp functional/basis set combination.

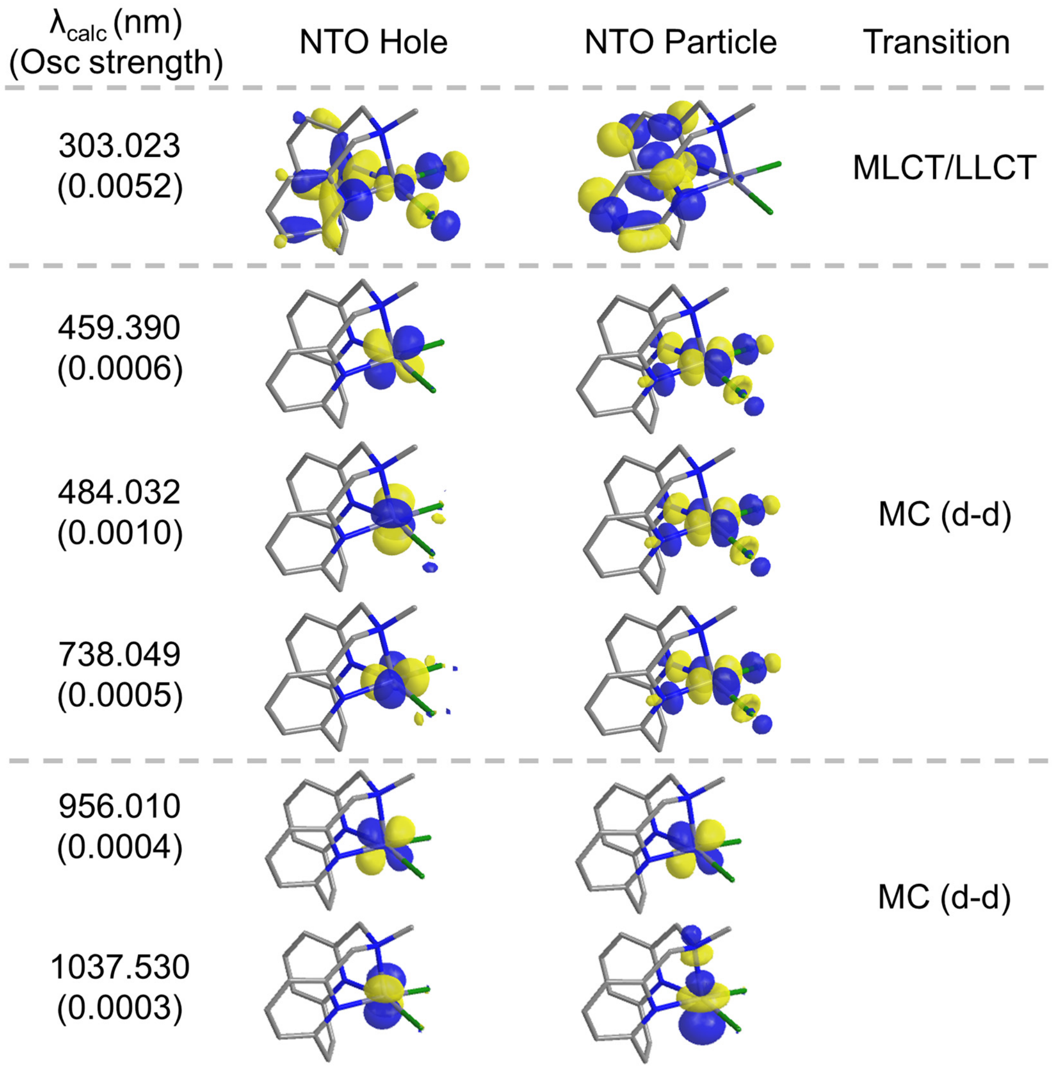

Supplementary Figure 78. Natural transition orbitals (NTOs) of (^{Me}N3)Ni^{II}Cl$_2$ (**1a**). The nature of optically active excited states corresponding to the transitions of interest are shown based on TD-DFT calculations employing the ωB97xD/def2tzvp functional/basis set combination.

2. Supplementary Discussion

2.1 Thermodynamic feasibility examination of the photocatalytic process

Supplementary Table 23. Redox Potentials of Photocatalyst, Nickel complexes and Quinuclidine[a]

Compounds	Reductive Quenching		Oxidative Quenching		Product
	*M/M⁻ (V)	M/M⁻ (V)	M⁺/*M (V)	M⁺/M (V)	yield (%)
$[Ru(bpz)_3](PF_6)_2$	1.45	–0.80	–0.26	1.86	0
$[Ru(phen)_3]Cl_2$	0.82	–1.36	–0.87	1.26	0.9 (trace)
$[Ir(ppy)_2(dtbbpy)]PF_6$	0.66	–1.51	–0.96	1.21	98
fac-$Ir(ppy)_3$	0.31	–2.19	–1.73	0.77	90
$[Ir(dF(CF_3)_2ppy)(dtbbpy)]PF_6$	1.21	–1.37	–0.89	1.69	86
$(^{iPr}N3)NiCl_2$ (**1b**) $Ni^{II/I}$		–0.82[b]			
$(^{iPr}N3)NiCl_2$ (**1b**) $Ni^{III/II}$				1.11	
$(^{iPr}N3)Ni(PhAc)Br$ (**4**) $Ni^{III/II}$				0.06	
$Quinuclidine^{\bullet+}/Q$				1.09	

[a]All redox and excited-state redox potential were corrected from reported experimental values [10,11] using conversion constants for redox potentials[12], and reported *vs* SCE. [b]irreversibility of the peak prevents accurate assignment of the redox potential.

Based on photoredox/Ni dual catalysis with blue LED experiment results, we evaluated thermodynamic feasibility of photocatalysts in the C–O coupling reaction for additional mechanistic insight (Table S8). Regarding photoredox/Ni dual catalysis, two distinct mechanisms can be considered: a reductive quenching or an oxidative quenching mechanism.[10] First of all, the mechanism composed of photocatalyst reductive quenching M/*M/M⁻/M cycle and $Ni^0/Ni^{II}/Ni^{III}/Ni^I/Ni^0$ cycle has been considered. In our results, both of strong oxidant $[Ir(dF(CF_3)_2ppy)(dtbbpy)]PF_6$ ($E(*Ir^{III}/Ir^{II})$ = +1.21 V vs SCE) and weak oxidant *fac*-$Ir(ppy)_3$ ($E(*Ir^{III}/Ir^{II})$ = +0.31 V vs SCE) successfully generated the desire C–O coupled product. In this scenario, the Ni^{II} species involved as a catalytic intermediate would be $(^{R}N3)Ni^{II}Ar(OR)$, as proposed by MacMillan et al.[13] Considering redox potentials of Ni complexes and photocatalysts (Ir and Ru complexes), it implies that the reductive quenching pathway involving $[Ru(phen)_3]Cl_2$

(E(*Ru^{II}/Ru^{I}) = +0.82 V vs SCE) is also thermodynamically feasible, and therefore could mediate the reaction. However, only a trace amount of product was observed with [Ru(phen)$_3$]Cl_2, and the observed production yields contradicts the trend of oxidation strength of excited photocatalysts ([Ir(dF(CF_3)$_2$ppy)(dtbbpy)]PF_6 > [Ru(phen)$_3$]Cl_2 > *fac*-Ir(ppy)$_3$). Therefore, we propose that the reductive quenching cycle might not be operating in our system. The other possibility includes oxidative quenching of photocatalyst *M/M^{+}/M/*M cycle and Ni^{II}/Ni^{I}/Ni^{III}/Ni^{I}/Ni^{II} cycle. In this case, the key step in the catalytic cycle is the single-electron reduction of Ni catalyst by the excited photocatalyst. As seen in our results, all the photocatalysts with excited state reduction potential E(M^{+}/*M) < –0.87 V successfully afforded a significant amount of C–O coupling product. Although our experimentally obtained redox potential of $Ni^{II/I}$ (–0.82 V vs SCE), which is slightly less negative than that of [Ru(phen)$_3$]Cl_2 (E(Ru^{III}/*Ru^{II}) = –0.87 V vs SCE), suggests SET process between Ni and *Ru is thermodynamically feasible, we believe that irreversibility of $Ni^{II/I}$ peak prevents correct assignment of the reduction potential and values are fall within the margin of error. In addition, the possibility of accessing Ni^{I} species via reduction by the highly reducing Ir^{II} species (generated through the reaction between *Ir^{III} and quinuclidine) was considered. Since for quinuclidine E ($Q^{\bullet+}$/Q) =+1.09 V, the reaction between quinuclidine and the excited photocatalyst will only occur when the E(*M/M^{-}) of the photocatalyst is larger than +1.09 V, which is the case for only two of the photocatalysts examined in our study, [Ru(bpz)$_3$](PF_6)$_2$ and [Ir(dF(CF_3)$_2$ppy)(dtbbpy)]PF_6. While [Ir(dF(CF_3)$_2$ppy)(dtbbpy)]PF_6 successfully generated the C–O product, [Ru(bpz)$_3$](PF_6)$_2$ failed to give C–O coupling product. Although the failure of [Ru(bpz)$_3$](PF_6)$_2$ to give C–O product could be due to an insufficient thermodynamic driving force to reduce Ni^{II} to Ni^{I} (E(Ru^{II}/Ru^{I}) = –0.80 V and E(Ni^{II}/Ni^{I}) = 0.82 V are very close in value), we could observe the C–O product formation with *fac*-Ir(ppy)$_3$ and [Ir(ppy)$_2$(dtbbpy)]PF_6, for which *Ir^{III} cannot be quenched by quinuclidine (E(*Ir^{III}/Ir^{II}) = +0.31 V and 0.66 V, respectively). Therefore, we consider that the reductive quenching cycle employing quinuclidine is less likely to be operating during this catalytic reaction. Overall, we postulate that the likely mechanistic pathway may not involve a Ni^{II} to Ni^{III} oxidation by the excited photocatalyst, and instead it would involve a Ni^{II} to Ni^{I} SET reduction process. We note that for some photocatalysts (such as *fac*-Ir(ppy)$_3$), regeneration of photocatalyst (M^{+} to M) can also occur concurrently with quinuclidine oxidation (estimated oxidation potential E_p= +1.09 V vs SCE).[14]

3. Supplementary References

1. Evans, D. F. Determination of the Paramagnetic Susceptibility of Substances in Solution By NMR. *J. Chem. Soc.*, 2003-2005 (1959).
2. Loliger, J. & Scheffold, R. Paramagnetic Moment Measurements by NMR - A micro technique. *J. Chem. Educ.* **49**, 646-647 (1972).
3. Wessel, A. J., Schultz, J. W., Tang, F., Duan, H. & Mirica, L. M. Improved synthesis of symmetrically & asymmetrically N-substituted pyridinophane derivatives. *Org. Biomol. Chem.* **15**, 9923-9931 (2017).
4. Sun, R. *et al.* Elucidation of a Redox-Mediated Reaction Cycle for Nickel-Catalyzed Cross Coupling. *J. Am. Chem. Soc.* **141**, 89-93 (2019).
5. Kuhn, H. J., Braslavsky, S. E. & Schmidt, R. Chemical actinometry (IUPAC Technical Report). *Pure Appl. Chem.* **76**, 2105-2146 (2004).
6. Montalti, M., Credi, A., Prodi, L. & Gandolfi, M. T. *Handbook of photochemistry*. (CRC press, 2006).
7. Hatchard, C. G., Parker, C. A. & Bowen, E. J. A new sensitive chemical actinometer - II. Potassium ferrioxalate as a standard chemical actinometer. *Proc. Royal Soc. London. A. Math. Phys. Sci.* **235**, 518-536 (1956).
8. Yayla, H. G. *et al.* Discovery and mechanistic study of a photocatalytic indoline dehydrogenation for the synthesis of elbasvir. *Chem. Sci.* **7**, 2066-2073 (2016).
9. Cloutier, J. P. & Zargarian, D. Functionalization of the Aryl Moiety in the Pincer Complex (NCN)(NiBr2)-Br-III: Insights on Ni-III-Promoted Carbon-Heteroatom Coupling. *Organometallics* **37**, 1446-1455 (2018).
10. Shon, J.-H. & Teets, T. S. Photocatalysis with Transition Metal Based Photosensitizers. *Comments Inorg. Chem.* **40**, 53-85 (2020).
11. Prier, C. K., Rankic, D. A. & MacMillan, D. W. Visible light photoredox catalysis with transition metal complexes: applications in organic synthesis. *Chem. Rev.* **113**, 5322-5363 (2013).
12. Pavlishchuk, V. V. & Addison, A. W. Conversion constants for redox potentials measured versus different reference electrodes in acetonitrile solutions at 25°C. *Inorg. Chim. Acta* **298**, 97-102 (2000).
13. Terrett, J. A., Cuthbertson, J. D., Shurtleff, V. W. & MacMillan, D. W. C. Switching on elusive organometallic mechanisms with photoredox catalysis. *Nature* **524**, 330-334 (2015).
14. Jeffrey, J. L., Terrett, J. A. & MacMillan, D. W. O-H hydrogen bonding promotes H-atom transfer from alpha C-H bonds for C-alkylation of alcohols. *Science* **349**, 1532-1536 (2015).
15. Bruker. APEXIII. Bruker AXS, Inc. *Madison, Wisconsin, USA* (2018).
16. Krause, L., Herbst-Irmer, R., Sheldrick, G. M. & Stalke, D. Comparison of silver and molybdenum microfocus X-ray sources for single-crystal structure determination. *J. Appl. Crystallogr.* **48**, 3-10 (2015).
17. Sheldrick, G. SHELXT - Integrated space-group and crystal-structure determination. *Acta Cryst. Sect. A* **71**, 3-8 (2015).
18. Spek, A. Structure validation in chemical crystallography. *Acta Cryst. Sect. D* **65**, 148-155 (2009).
19. Frisch, M. J. *et al.* Gaussian 16 Rev. B.01. *Gaussian 16 Rev. B.01*, Gaussian, Inc., Wallingford CT (2016).

20. Zhao, Y. & Truhlar, D. G. The M06 suite of density functionals for main group thermochemistry, thermochemical kinetics, noncovalent interactions, excited states, and transition elements: two new functionals and systematic testing of four M06-class functionals and 12 other functionals. *Theor. Chem. Acc.* **120**, 215-241 (2008).
21. Schafer, A., Horn, H. & Ahlrichs, R. Fully Optimized Contracted Gaussian Basis Sets for Atoms Li to Kr. *J. Chem. Phys.* **97**, 2571-2577 (1992).
22. Schafer, A., Huber, C. & Ahlrichs, R. Fully Optimized Contracted Gaussian Basis Sets of Triple Zeta Valence Quality for Atoms Li to Kr. *J. Chem. Phys.* **100**, 5829-5835 (1994).
23. Shields, B. J., Kudisch, B., Scholes, G. D. & Doyle, A. G. Long-Lived Charge-Transfer States of Nickel(II) Aryl Halide Complexes Facilitate Bimolecular Photoinduced Electron Transfer. *J. Am. Chem. Soc.* **140**, 3035-3039 (2018).
24. Ting, S. I. *et al.* 3d-d Excited States of Ni(II) Complexes Relevant to Photoredox Catalysis: Spectroscopic Identification and Mechanistic Implications. *J. Am. Chem. Soc.* **142**, 5800-5810 (2020).
25. Becke, A. D. Density-functional thermochemistry. III. The role of exact exchange. *J. Chem. Phys.* **98**, 5648-5652 (1993).
26. Lee, C. T., Yang, W. T. & Parr, R. G. Development of the Colle-Salvetti Correlation-Energy Formula Into a Functional of the Electron-Density. *Phys. Rev. B- Cond. Matter* **37**, 785-789 (1988).
27. Yanai, T., Tew, D. P. & Handy, N. C. A new hybrid exchange–correlation functional using the Coulomb-attenuating method (CAM-B3LYP). *Chem. Phys. Lett.* **393**, 51-57 (2004).
28. Chai, J.-D. & Head-Gordon, M. Long-range corrected hybrid density functionals with damped atom–atom dispersion corrections. *Phys. Chem. Chem. Phys.* **10**, 6615-6620 (2008).
29. Weigend, F. & Ahlrichs, R. Balanced basis sets of split valence, triple zeta valence and quadruple zeta valence quality for H to Rn: Design and assessment of accuracy. *Phys. Chem. Chem. Phys.* **7**, 3297-3305 (2005).
30. Weigend, F. Accurate Coulomb-fitting basis sets for H to Rn. *Phys. Chem. Chem. Phys.* **8**, 1057-1065 (2006).
31. Skripnikov, L. Chemissian, version 4.67, 2020 (www.chemissian.com, accessed March 2021).
